# Bni5 regulates and coordinates septin architecture and myosin-II functions at the cell division site

**DOI:** 10.1083/jcb.202311040

**Published:** 2025-11-06

**Authors:** Hiroki Okada, Xi Chen, Joseph Marquardt, Kangji Wang, Erfei Bi

**Affiliations:** 1 Department of Cell and Developmental Biology, Perelman School of Medicine at the University of Pennsylvania, Philadelphia, PA, USA

## Abstract

The spatiotemporal coordination of septins and myosin-II in processes like cytokinesis is not well understood. In *Saccharomyces cerevisiae*, Bni5 links the myosin-II heavy chain Myo1 to the septin hourglass at the bud neck prior to cytokinesis, but the underlying mechanisms and functions remain unclear. Here, we show that Bni5 binds septin filaments, the septin-associated kinase Elm1, and Myo1 via distinct domains. Bni5 regulates the architecture and stability of the septin hourglass until it dissociates from the bud neck at the onset of cytokinesis. This dissociation, facilitated through phosphorylation of Bni5 by Gin4, an Elm1-interacting kinase, enables timely remodeling of the septin hourglass into a double ring. Bni5 also mediates the role of Myo1 in retrograde actin cable flow during polarized growth and ensures maximal accumulation of Myo1 at the bud neck before cytokinesis, reinforcing the actomyosin ring and buffering it against perturbations. These findings establish Bni5 as a key regulator and coordinator of septins and myosin-II at the division site.

## Teaser

Okada et al. show that Bni5 interacts with septin filaments, septin-associated kinase, and myosin-II to regulate septin hourglass architecture and remodeling, retrograde actin flow, and cytokinesis, establishing Bni5 as a key linker and regulator of septins and myosin-II at the division site.

## Introduction

Septins and non-muscle myosin-II (NM-II) play crucial roles in diverse processes, including cytokinesis, cell migration, cell adhesion, and membrane trafficking (Benoit et al., 2023; Dolat et al., 2014; Marquardt et al., 2021; McMurray and Thorner, 2009; Pecci et al., 2018; Shutova and Svitkina, 2018; Vicente-Manzanares et al., 2009). Septins function by assembling into filaments and higher-order structures, such as rings and hourglasses, where they act as scaffolds and/or diffusion barriers (Barral et al., 2000; Dobbelaere and Barral, 2004; Gladfelter et al., 2001; Longtine et al., 1996; Wloka et al., 2011). NM-II forms bipolar filaments that slide actin filaments passing each other to generate contractile forces that dictate cell shape, motility, and division (Pecci et al., 2018; Shutova and Svitkina, 2018; Vicente-Manzanares et al., 2009). However, the molecular coordination between septins and NM-II in these processes remains largely unknown, with the exception of cytokinesis. During cytokinesis in metazoans, the scaffold protein anillin binds directly to both septins and NM-II via distinct domains (D’Avino et al., 2008; Field and Alberts, 1995; Oegema et al., 2000; Piekny and Maddox, 2010; Straight et al., 2005), regulating their recruitment and organization at the division site (D’Avino et al., 2008; Garno et al., 2021; Oegema et al., 2000; Straight et al., 2005). In the absence of anillin, the stability and contractility of the actomyosin ring (AMR) are compromised, resulting in aberrant furrowing, particularly during late stages of cytokinesis (Echard et al., 2004; Field and Alberts, 1995; Field et al., 2005; Maddox et al., 2007; Renshaw et al., 2014; Wang et al., 2023).

In addition to anillin, Bni5 in the budding yeast *Saccharomyces cerevisiae* is the only other protein known to directly bind both septins and myosin-II (Fang et al., 2010; Lee et al., 2002). The five mitotic septins in *S. cerevisiae* form two distinct octamers or heteromeric complexes: Cdc11–Cdc12–Cdc3–Cdc10–Cdc10–Cdc3–Cdc12–Cdc11 and Shs1–Cdc12–Cdc3–Cdc10–Cdc10–Cdc3–Cdc12–Shs1. The Cdc11-capped octamers form paired filaments, while the Shs1-capped octamers stagger into ring structures in vitro (Bertin et al., 2008; Frazier et al., 1998; Garcia et al., 2011). These octamers assemble into a nascent ring at the site of cell polarization in vivo. Upon bud emergence, the nascent ring expands into an hourglass structure at the bud neck, where it remains until cytokinesis. The mitotic exit network (MEN) triggers its remodeling into a double ring that sandwiches the AMR during cytokinesis (Cid et al., 2001; Kim et al., 1991; Lippincott et al., 2001; Ong et al., 2014). Bni5, together with two kinases—Elm1 (an LKB1/PAR-4–related kinase) and Gin4 (a Nim1/PAR-1–related kinase)—localizes to the septin hourglass and dissociates from the bud neck before the hourglass-to-double ring (HDR) transition (Lee et al., 2002; Marquardt et al., 2024; Patasi et al., 2015). Both Elm1 and Gin4 affect septin organization (Bouquin et al., 2000; Longtine et al., 1998a; Marquardt et al., 2024; Marquardt et al., 2020) and reciprocally regulate each other during the cell cycle (Marquardt et al., 2024). However, the molecular mechanism underlying the interaction of Bni5 with septin filaments and its role in septin hourglass assembly and remodeling remains unclear. In addition, the regulation of Bni5 by the Elm1 and Gin4 kinases and the functional consequence of this regulation have yet to be fully explored.

Myo1 is the sole myosin-II heavy chain in budding yeast, required for efficient cytokinesis and cell separation, though not for cell viability (Bi et al., 1998; Lippincott and Li, 1998; Rodriguez and Paterson, 1990; Watts et al., 1987). Myo1 is targeted to the division site through a two-step process: Bni5 mediates its targeting from bud emergence to the onset of cytokinesis, while Iqg1, the essential IQGAP in budding yeast, mediates its targeting from anaphase to the completion of cytokinesis (Fang et al., 2010). These two targeting mechanisms overlap from the onset of anaphase to the onset of telophase or cytokinesis, during which Bni5 gradually dissociates from the bud neck while Iqg1 accumulates there (Fang et al., 2010; Lee et al., 2002; Okada et al., 2021b). Thus, Bni5 serves as the sole linker between Myo1 and the septin hourglass before anaphase. Myo1 promotes retrograde flow of actin cables assembled at the bud cortex during bud growth (Huckaba et al., 2006), a process thought to support asymmetric inheritance of mitochondrial fitness between the mother and bud (Higuchi et al., 2013). However, how Bni5 interacts with Myo1 at the molecular level and whether Bni5 mediates the role of Myo1 in retrograde flow are unclear. It also remains unknown whether Bni5-mediated Myo1 accumulation at the bud neck plays any role in cytokinesis.

In this study, we have defined the timing and mechanisms of Bni5 interactions with septin filaments, the Elm1 kinase, and Myo1 during the cell cycle. We have also established the roles of Bni5 in regulating the architecture and remodeling of the septin hourglass and in mediating Myo1 functions in retrograde flow and cytokinesis.

## Results

### Localization and turnover kinetics of Bni5 in relation to septins and myosin-II at the division site during the cell cycle

To investigate how Bni5 may regulate septin and myosin-II function at the division site, we first analyzed their localization and turnover kinetics at the bud neck during the cell cycle. Previous studies using FRAP revealed the high stability of septins (Caviston et al., 2003; Dobbelaere et al., 2003), contrasting the dynamic behavior of myosin-II (Myo1) and Bni5 at the bud neck before cytokinesis (Schneider et al., 2013; Wloka et al., 2013). However, the dynamic nature of Bni5 cannot easily explain its stable association with septin complexes, as indicated by co-purification studies showing stoichiometric proportions of Bni5 and septins (Mortensen et al., 2002; Renz et al., 2016).

**Figure 1.**
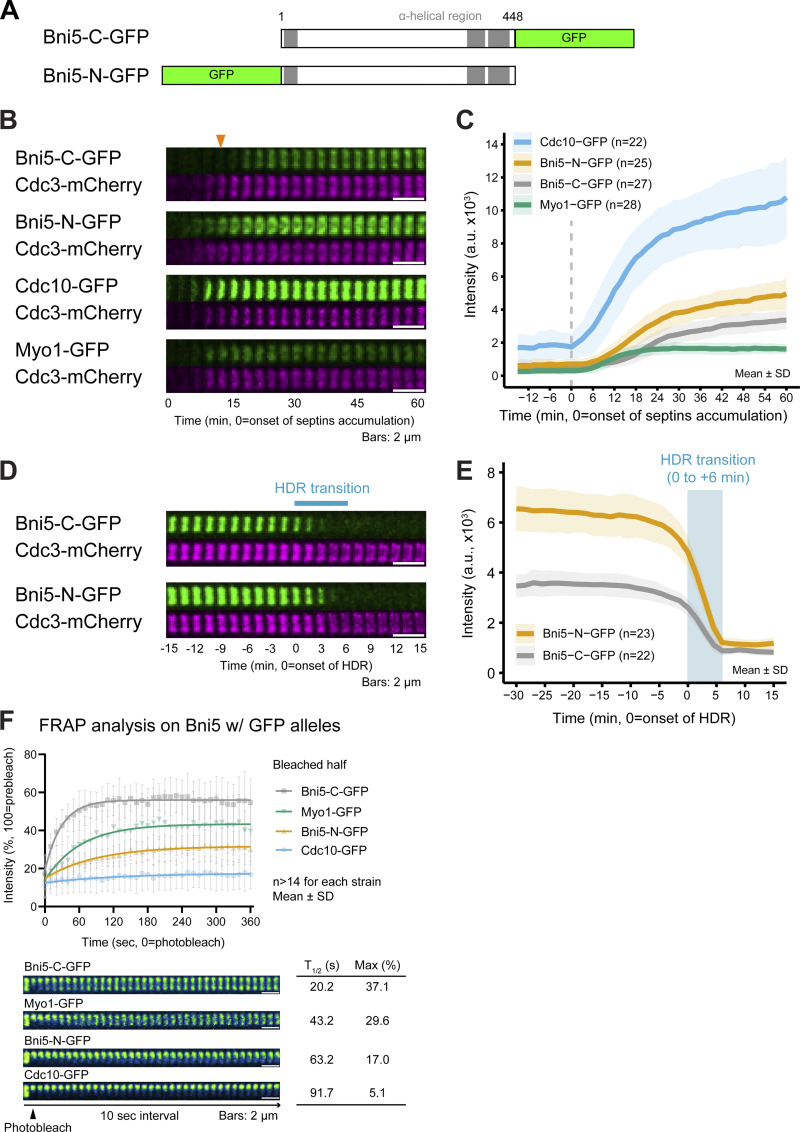
**Distinct impact of N- and C-terminal GFP-tagging of Bni5 on its recruitment timing and turnover kinetics at the division site. (A)** Diagram of GFP-tagged Bni5 constructs. Bni5-C-GFP and Bni5-N-GFP indicate GFP inserted at their C and N terminus, respectively. Numbers indicate aa residues. Gray boxes represent predicted α-helical regions. **(B)** Time-lapse analysis of Bni5-C-GFP and Bni5-N-GFP accumulation at the budding site. Montages of GFP-tagged proteins with respect to Cdc3-mCherry were created from selected frames of time-lapse series taken with 1.5-min intervals. Arrowhead indicates the onset of Bni5-C-GFP accumulation. Strains used: YEF9336 (*BNI5-C-GFP CDC3-mCherry*), YEF10293 (*BNI5-N-GFP CDC3-mCherry*), YEF11750 (*CDC10-GFP CDC3-mCherry*), and YEF6108 (*MYO1-GFP CDC3-mCherry*). **(C)** Recruitment kinetics of GFP-tagged proteins during bud formation, quantified from cells shown in B. *n* denotes the number of cells analyzed per strain. Bold lines and shaded bands represent the mean and SD, respectively. **(D)** Localization dynamics of Bni5-C-GFP and Bni5-N-GFP during cytokinesis. Montages were created as in B. The blue bar indicates the period of the septin HDR transition. Strains used: YEF9336 (*BNI5-C-GFP CDC3-mCherry*) and YEF10293 (*BNI5-N-GFP CDC3-mCherry*). **(E)** Localization kinetics Bni5-C-GFP and Bni5-N-GFP during cytokinesis, quantified from cells shown in D. *n* denotes the number of cells analyzed per strain. Bold lines and shaded bands represent the mean and SD, respectively. The blue box indicates the period of the septin HDR transition. **(F)** FRAP analysis of Bni5-C-GFP and Bni5-N-GFP to assess turnover kinetics at the bud neck. Cells with a medium-sized bud and a short spindle were chosen for FRAP analysis. Top: Lines, symbols, and error bars represent recovery curves for indicated proteins, showing mean values and SD, respectively. *n* denotes the number of cells analyzed per strain. Bottom: Montages show selected frames from the time-lapse series acquired with 10-s intervals. Max and T_1/2_ indicate the estimated maximum amount of recovery and half-time of recovery. Strains used: YEF11624 (*BNI5-C-GFP mScarlet-TUB1*), YEF11546 (*BNI5-N-GFP mScarlet-TUB1*), YEF11753 (*CDC10-GFP mScarlet-TUB1*), and YEF11385 (*MYO1-GFP mScarlet-TUB1*).

**Figure S1.**
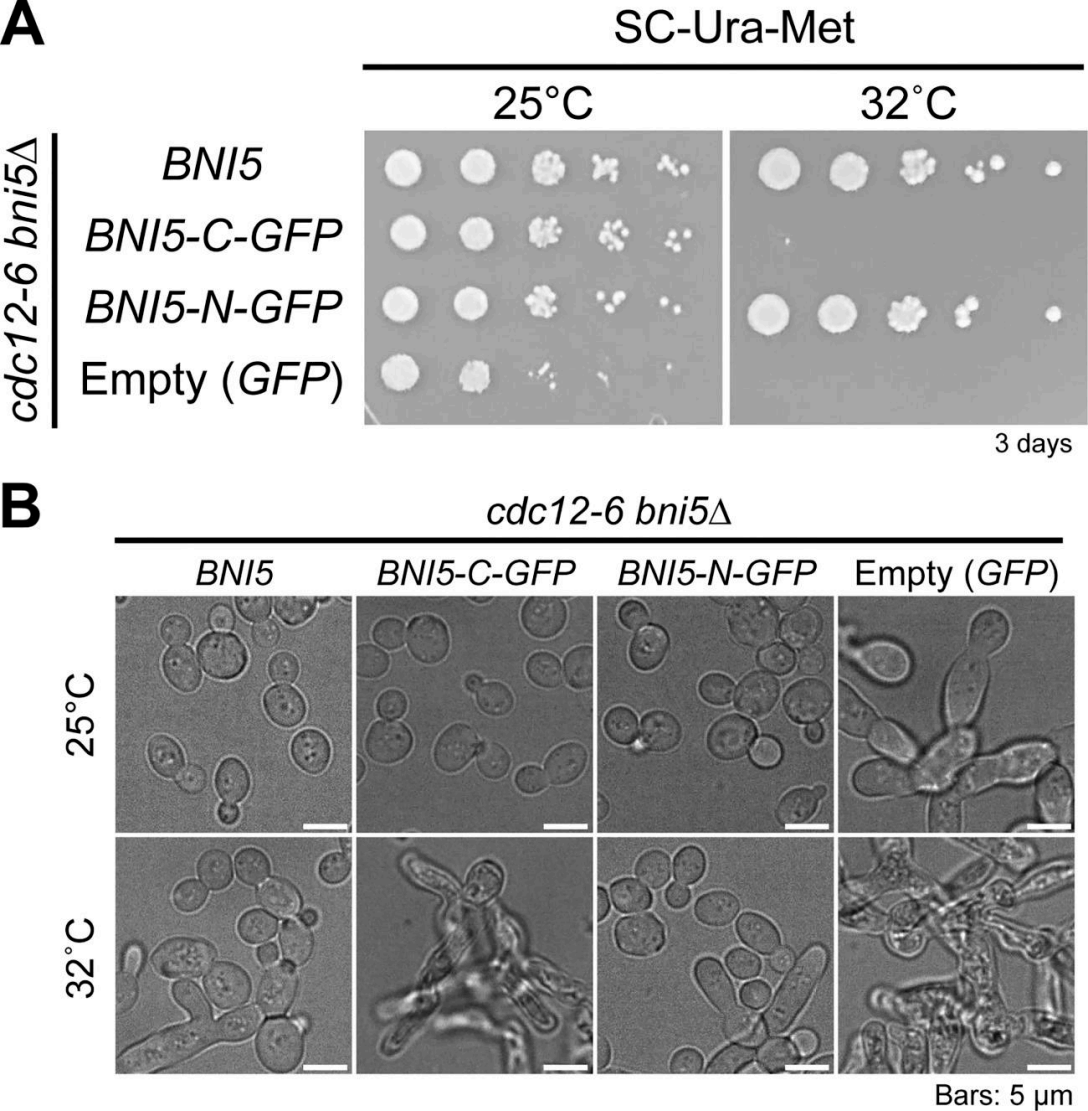
**Cell growth and morphological suppression of the *cdc12-6* septin mutant by overexpression of Bni5-N-GFP, but not Bni5-C-GFP.** Related to Fig. 2. **(A)** Effects of overexpression of different *BNI5* alleles on the temperature sensitivity of *cdc12-6* cells. Cells were grown in methionine-depleted medium to induce *BNI5 *gene expression. Strains used: YEF11362 (*cdc12-6 bni5Δ *[*BNI5*]), YEF11363 (*cdc12-6 bni5Δ *[*BNI5-C-GFP*]), YEF11312 (*cdc12-6 bni5Δ *[*BNI5-N-GFP*]), and YEF11313 (*cdc12-6 bni5Δ *[*GFP*]). **(B)** Representative images of *cdc12-6* cells with induced expression of different *BNI5* alleles. Cells were grown in SC-Ura-Met liquid medium to induce their expression at 25°C or 32°C for 16 h before imaging. Strains used are the same as in A.

All previous analyses involved Bni5-C-GFP (where *GFP* is inserted before the stop codon of *BNI5* at its chromosomal locus) (Fig. 1 A) (Schneider et al., 2013; Wloka et al., 2013), but C-terminal tagging of Bni5 may impair its function (Finnigan et al., 2015). We therefore tested the functionality of both C- and N-terminally tagged versions of *BNI5*, Bni5-C-GFP, and Bni5-N-GFP, respectively, by overexpressing them in the temperature-sensitive septin mutant *cdc12-6*. Overexpression of Bni5-N-GFP or untagged Bni5, but not Bni5-C-GFP, effectively rescued the growth and morphological defects observed in *cdc12-6* at both permissive (25°C) and restrictive (32°C) temperatures (Fig. S1, A and B). This suggests that Bni5-C-GFP may have a defect in its association with septins.

To address this, we generated an N-terminally tagged version of *BNI5* at its chromosomal locus, resulting in the expression of Bni5-N-GFP under the control of its native promoter (Fig. 1 A). Time-lapse imaging showed that Bni5-N-GFP localized to the site of cell polarization (the budding site) simultaneously with the septins (Cdc10 and Cdc3) and Myo1 (Fig. 1, B and C). In contrast, Bni5-C-GFP exhibited a delay of ∼12 min in its localization, appearing around bud emergence (Fig. 1, B and C, arrowhead). This suggests that Bni5-C-GFP fails to associate with septins during the early stages of cell polarization. Furthermore, Bni5-N-GFP accumulated ∼32% more at the bud neck than Bni5-C-GFP (Fig. 1 C, at time point 60 min), highlighting the deficiency of Bni5-C-GFP in interacting with septins. Both Bni5-N-GFP and Bni5-C-GFP began to dissociate from the bud neck 10 min before the onset of the septin HDR transition, initially slowly and then more rapidly, and were completely absent by the end of this transition (Fig. 1, D and E). These results suggest that Bni5 functions in relation to septins and myosin-II from the time of cell polarization to the onset of cytokinesis.

FRAP analysis revealed that Bni5-N-GFP turned over slowly, similar to Cdc10-GFP, while Bni5-C-GFP turned over rapidly, resembling Myo1 before anaphase (Fig. 1 F) (Wloka et al., 2013). The slower turnover of Bni5-N-GFP supports a stable association between endogenous Bni5 and septin complexes, as observed in co-purification studies (Mortensen et al., 2002; Renz et al., 2016). These findings suggest that Bni5 acts as a stable linker, connecting the dynamic myosin-II to the stable septin hourglass at the bud neck before cytokinesis.

### The septin-associated kinase Elm1 is essential for the localization of Bni5 at the bud neck when the Bni5–septin association is disrupted

**Figure 2.**
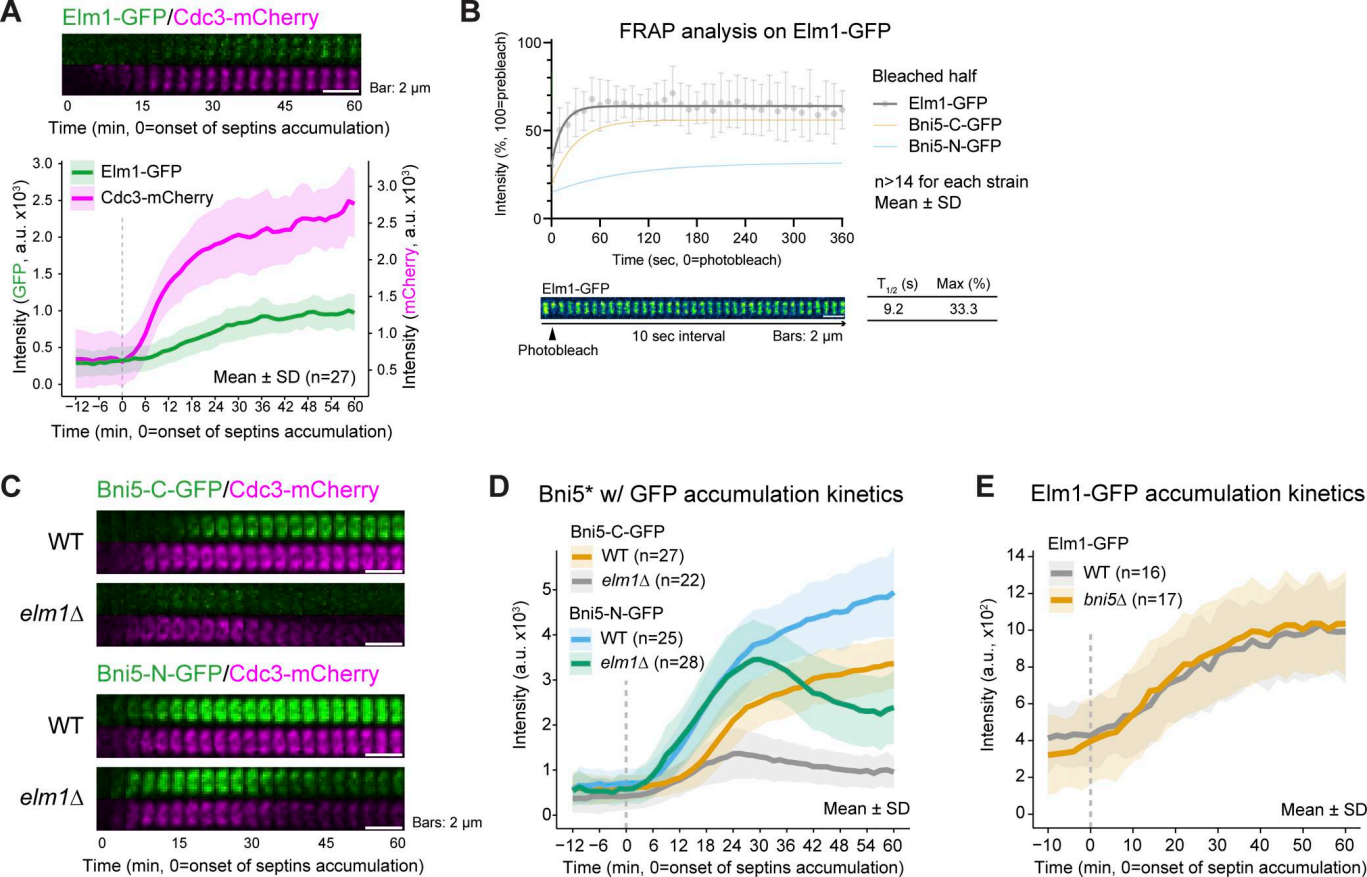
**Localization and turnover kinetics of Elm1 and its role in recruiting Bni5 to the bud neck when Bni5–septin association is disrupted. (A)** Elm1-GFP accumulation at the budding site. Top: Montages of Elm1-GFP with respect to Cdc3-mCherry were created from selected frames of time-lapse series taken with 1.5-min intervals. Bottom: Quantification of Elm1-GFP and Cdc3-mCherry signals. *n* denotes the number of cells analyzed per strain. Bold lines and shaded bands represent the mean and SD, respectively. Strain used: YEF10440 (*ELM1-GFP CDC3-mCherry*). **(B)** FRAP analysis of Elm1-GFP. Top: Lines, symbols, and error bars represent recovery curves, showing mean values and SD, respectively. *n* denotes the number of cells analyzed per strain. The reference plots of Bni5-C-GFP and Bni5-N-GFP were modified from Fig. 1 F. Bottom: Montages were created from time-lapse series taken with 10-s intervals. Max and T_1/2_ indicate the estimated maximum amount of recovery and half-time of recovery. Strain used: YEF8437 (*ELM1-GFP mRuby2-TUB1*). **(C)** Time-lapse analysis of Bni5-C-GFP and Bni5-N-GFP accumulation at the budding site in *elm1Δ* cells. Montages were created as described in A. Strains used: YEF9336 (*BNI5-C-GFP CDC3-mCherry*), YEF9369 (*elm1Δ BNI5-C-GFP CDC3-mCherry*), YEF10293 (*BNI5-N-GFP CDC3-mCherry*), and YEF10315 (*elm1Δ BNI5-N-GFP CDC3-mCherry*). **(D)** Quantification of GFP-tagged protein recruitment to the bud neck from cells shown in C. *n* denotes the number of cells analyzed per strain. Bold lines and shaded bands represent the mean and SD, respectively. Asterisk indicates variants of Bni5. **(E)** Elm1-GFP accumulation at the budding site in *bni5Δ* cells. *n* denotes the number of cells analyzed per strain. Bold lines and shaded bands represent the mean and SD, respectively. Strains used: YEF9305 (*ELM1-GFP CDC3-mCherry*) and YEF9313 (*ELM1-GFP CDC3-mCherry bni5Δ*).

The absence of Bni5-C-GFP association with septins before bud emergence, coupled with their co-localization afterward, suggests that a newly localized factor around bud emergence may tether Bni5-C-GFP to septins at later stages. The septin-associated kinase Elm1 is a strong candidate for this role, as it localized to the bud neck around bud emergence (Fig. 2 A) (Marquardt et al., 2024) and exhibited rapid turnover (Fig. 2 B). Furthermore, Elm1 specifically associates with the septin hourglass from bud emergence to the onset of cytokinesis (Bouquin et al., 2000; Kang et al., 2016; Marquardt et al., 2024; Marquardt et al., 2020; Patasi et al., 2015). All these characteristics closely resemble those of Bni5-C-GFP, leading us to test whether Elm1 is required for the localization of Bni5-C-GFP at the bud neck.

In *elm1Δ* cells, Bni5-C-GFP nearly completely failed to localize to the bud neck (Fig. 2, C and D), suggesting that Elm1 is the primary, if not sole, mediator between Bni5-C-GFP and the septins. In contrast, Bni5-N-GFP localized to the bud neck in *elm1Δ* cells as efficiently as in WT cells during early budding (Fig. 2, C and D). However, it partially delocalized with septins and co-migrated to the bud tip after 28.5 min (Fig. 2, C and D) (Marquardt et al., 2020). In contrast, in the absence of Bni5, the localization of Elm1 to the bud neck remained unaffected (Fig. 2 E). These results indicate that Bni5 typically localizes to the bud neck by associating with septins from cell polarization to the onset of cytokinesis. When this association is disrupted, Elm1 becomes essential for the localization of Bni5 at the bud neck after bud emergence.

### Bni5 interacts with septin filaments and Elm1 via overlapping regions at its C terminus

**Figure 3.**
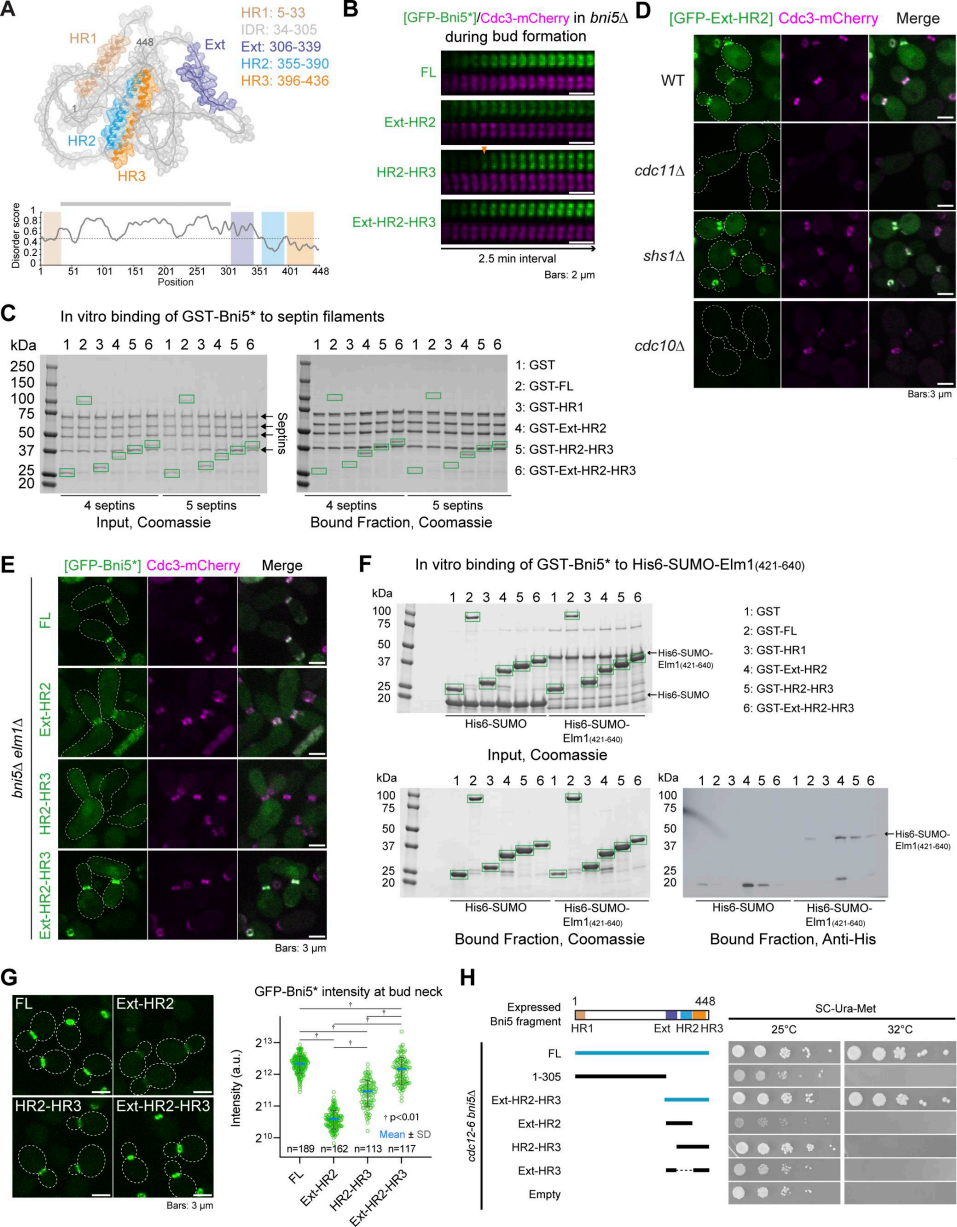
**Bni5 interacts with septin filaments and Elm1 via overlapping regions at its C terminus. (A)** Diagram of Bni5 structural features. Top: AlphaFold-predicted structure of Bni5. The HR1, IDR, Ext, HR2, and HR3 regions are color-coded in brown, gray, purple, blue, and orange, respectively. Bottom: IUPred3 prediction of intrinsic disorder. Scores above the dotted line indicate a higher probability of disorder. Shaded boxes correspond to the same regions as in the structural model. **(B)** Time-lapse analysis of Bni5 fragment co-localization with septins at the budding site. Montages of GFP-tagged Bni5 fragments with respect to Cdc3-mCherry were created from time-lapse series taken with 2.5-min intervals. Arrowhead indicates the onset of GFP-HR2-HR3 accumulation. Asterisk indicates variants of Bni5. Strains used: YEF11053 (*bni5Δ CDC3-mCherry* [*GFP-BNI5(FL)*]), YEF11181 (*bni5Δ CDC3-mCherry* [*GFP-bni5(306-339)*]), YEF11040 (*bni5Δ CDC3-mCherry* [*GFP-bni5(340-448)*]), and YEF11039 (*bni5Δ CDC3-mCherry* [*GFP-bni5(306-448)*]). **(C)** Bni5 binds to septin filaments via Ext-HR2. Left: SDS-PAGE stained with Coomassie blue, showing purified proteins used as input for in vitro–binding assay. Right: In vitro–binding assay results (Coomassie blue–stained gel) for indicated GST-tagged proteins co-sedimented with septin filaments by ultracentrifugation; kDa = kiloDalton. Green boxes represent GST-fusion proteins. Asterisk indicates variants of Bni5. A representative result from three independent experiments is shown. See also Fig. S2, B and C. **(D)** Localization of GFP-Ext-HR2 in WT and septin mutants. Cells in the exponential growth phase were imaged. Strains used: YEF11207 (*BNI5 CDC3-mCherry* [*GFP-BNI5*(306-393)]), YEF11819 (*BNI5 cdc11Δ CDC3-mCherry* [*GFP-BNI5*(306-393)]), YEF11811 (*BNI5 shs1Δ CDC3-mCherry* [*GFP-BNI5*(306-393)]), and YEF11812 (*BNI5 cdc10Δ CDC3-mCherry* [*GFP-BNI5*(306-393)]). See also Fig. S2 A. **(E)** Localization of Bni5 fragments in *bni5Δ elm1Δ* cells. Cells in the exponential growth phase were imaged. Strains used: YEF11190 (*bni5Δ elm1Δ CDC3-mCherry* [*GFP-BNI5(FL)*]), YEF11197 (*bni5Δ elm1Δ CDC3-mCherry* [*GFP-bni5(306-393)*]), YEF11194 (*bni5Δ elm1Δ CDC3-mCherry* [*GFP-bni5(340-448)*]), and YEF11193 (*bni5Δ elm1Δ CDC3-mCherry* [*GFP-bni5(306-448)*]). **(F)** HR2-containing fragments of Bni5 interact with Elm1. Top left: SDS-PAGE stained with Coomassie blue, showing purified proteins used in binding assay. Bottom left: In vitro–binding assay results (Coomassie blue–stained gel) for the indicated GST-tagged proteins bound to glutathione resin and their ability to pull down His-SUMO-Elm1(421-640). Green boxes represent GST and GST-tagged Bni5 fragments shown at top right. Bottom right: Immunoblotted membrane with antibody against His; kDa = kiloDalton. Asterisk indicates variants of Bni5. A representative result from three independent experiments is shown. **(G)** Quantification of Bni5 fragment localization at the bud neck. Left: Representative images of cells in the exponential growth phase. Right: Quantification of GFP signal intensities at the bud neck in cells with a small or medium bud (S/G2 cells). Green circles, blue bars, and gray bars represent individual data points, mean values, and SD, respectively. Asterisk indicates variants of Bni5. P values were determined using a two-sided Mann–Whitney U test. Strains used: YEF11821 [*GFP-BNI5(FL)*], YEF11823 [*GFP-bni5(306-393)*], YEF11824 [*GFP-bni5(340-448)*], and YEF11825 [*GFP-bni5(306-448)*]. **(H)** Dosage suppression of the *cdc12-6* septin mutant by Bni5 fragments. Left: Summary diagram showing suppression (blue) and non-suppression (black) by the indicated *BNI5* alleles. Bold lines and dashed lines represent the presence and absence of Bni5 regions, respectively. Right: Spot assay result after 3 days of incubation at 25°C or 32°C. Strains were generated by introducing the pUG36-Bni5* plasmid series (Table S2) into YEF11247 (*cdc12-6 bni5Δ*). Source data are available for this figure: SourceData F3.

To understand the mechanism and function of Bni5 in coupling septins to myosin-II during the cell cycle, we performed structure-function analyses to define the binding sites of Bni5 for these proteins. AlphaFold predicts that Bni5 contains three α-helical regions (HR1–3), one at the N terminus (HR1) and two near the C terminus (HR2 and HR3) (Fig. 3 A, top) (Jumper et al., 2021; Varadi et al., 2022), separated by an intrinsically disordered region (IDR), as predicted by IUPred3 (Fig. 3 A, bottom) (Erdos et al., 2021). Using this model and coupling genetic, cell biological, and biochemical analyses, we defined the binding sites of Bni5 for septin filaments and Elm1 (described below), as well as Myo1 (described later).

**Figure S2.**
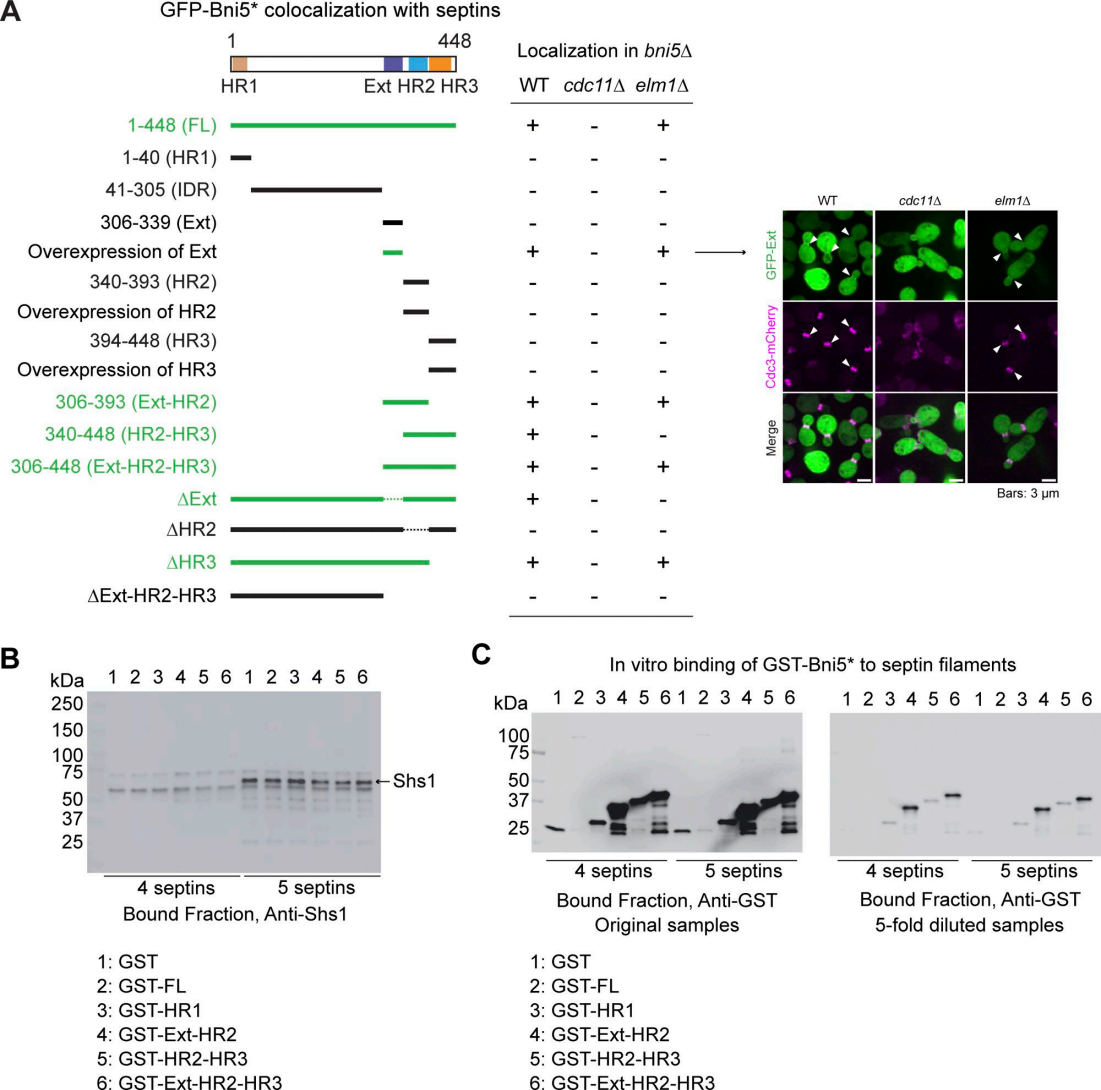
**Identification of septin- and Elm1-dependent localization domains in Bni5 and Western blot confirmation of Bni5 fragment interactions with four- and five-septin complexes.** Related to Fig. 3. **(A)** Colocalization analysis of various Bni5 fragments with septins. Imaging was performed in *bni5Δ*, *bni5Δcdc11Δ, *and* bni5Δelm1Δ* strain backgrounds. Colocalization between GFP-tagged Bni5 fragments and Cdc3-mCherry is indicated in a table as “+” (colocalization) or “−” (no colocalization). Fragments exhibiting colocalization in at least one strain background are highlighted in green. Bold and dashed lines indicate the presence and absence of specific regions in the corresponding Bni5 variants, respectively. For overexpression of Ext, HR2, and HR3 fragments, cells were grown in methionine-depleted SC medium for 5 h; representative images of Ext-overexpressed cells were shown beside the table. Asterisk indicates Bni5 variants. Plasmids from the pUG36-Bni5* series (Table S2) were introduced into the following strains: YEF10994 (*bni5∆ CDC3-mCherry*), YEF12138 (*bni5∆ cdc11Δ CDC3-mCherry*), and YEF11277 (*bni5∆ elm1∆ CDC3-mCherry*). **(B)** Western blot analysis using an anti-Shs1 antibody confirms the presence of Shs1 in the five-septin, but not in the four-septin, complexes used in our in vitro binding experiments. **(C)** Western blots using an anti-GST antibody to demonstrate the ability of different Bni5 fragments to bind septin filaments in vitro. Due to inefficient transfer of large-sized proteins from polyacrylamide gel to PVDF membrane, the GST-FL (Bni5) could be observed only when more samples were loaded for the western blot analysis (left). Source data are available for this figure: SourceData FS2.

Deletion analysis revealed a potential septin-binding site in Bni5 (Fig. S2 A). Similar to full-length (FL) Bni5, the Ext-HR2 fragment of Bni5, which contains HR2 and an extended upstream sequence (Ext) (Fig. 3 A), associated with septins at the bud neck throughout the cell cycle (Fig. 3 B). Importantly, overexpression of Ext alone—not HR2 or HR3—was sufficient to weakly localize to the bud neck in a Cdc11-dependent, but not Elm1-dependent, manner (Fig. S2 A), suggesting that Ext contains a septin-targeting motif. FL Bni5 is known to interact with septin filaments rather than individual septin complexes in vitro (Booth et al., 2016; Patasi et al., 2015; Renz et al., 2013). Similarly, recombinant Ext-HR2 interacted robustly with septin filaments made of Cdc11-capped octamers (four septins) or both Cdc11- and Shs1-capped octamers (five septins) (Fig. 3 C). Notably, adding Shs1 did not enhance this interaction (Fig. 3 C; and Fig. S2, B and C). The in vitro binding of Bni5 to septin filaments is thought to involve interactions with Cdc11 and Shs1, two alternate terminal subunits of septin octamers, but not with the core subunit Cdc10 (Booth et al., 2016; Booth et al., 2015; Finnigan et al., 2015; Lee et al., 2002). However, we observed that the localization of Ext-HR2 to the bud neck depended on Cdc11 and Cdc10, but not Shs1 (Fig. 3 D and Fig. S2 A). This pattern, which seems at odds with the in vitro data, can be explained by the possibility that Ext-HR2 may bind to septin filaments, but not septin complexes, in vivo. Septin filaments have been observed at the bud neck of *shs1Δ* cells (Bertin et al., 2012; Ong et al., 2014), but not in *cdc11Δ* or *cdc10Δ* cells (Frazier et al., 1998). These findings define Ext-HR2 as the septin filament–binding site for Bni5.

The same deletion analysis also identified the potential Elm1-binding site in Bni5 (Fig. S2 A). Like Elm1, the HR2-HR3 fragment of Bni5 localized to the budding site with a delay relative to septin recruitment (Fig. 3 B, arrowhead). This localization was completely abolished in the absence of *ELM1*, in contrast to the septin-binding fragments, Ext-HR2 and Ext-HR2-HR3 (Fig. 3 E). Recombinant HR2-HR3 interacted—albeit weakly—with the C-terminal non-kinase domain of Elm1 (Fig. 3 F, bottom-right image, lane 5), which is known to mediate its bud neck localization (Marquardt et al., 2024; Marquardt et al., 2020; Moore et al., 2010). Surprisingly, Ext-HR2 also showed weak interaction with the same region of Elm1 (Fig. 3 F, bottom-right image, lane 4), suggesting that HR2 is crucial for the interactions of Bni5 with both septin filaments and Elm1. Indeed, FL Bni5 lacking HR2, but not Ext or HR3, completely failed to localize to the bud neck (Fig. S2 A). Thus, HR2-HR3 defines the Elm1-binding site for Bni5, while HR2 is essential for the binding of Bni5 to both septin filaments and Elm1.

While Ext-HR2 and HR2-HR3 localized to the bud neck in a septin filament– and Elm1-dependent manner, respectively, neither could fully account for the localization of FL Bni5 (Fig. 3 G). However, when combined, the Ext-HR2-HR3 fragment localized to the bud neck nearly as efficiently as FL Bni5 (Fig. 3 G). As expected, Ext-HR2-HR3 interacted with both septin filaments and Elm1 in vitro (Fig. 3, C and F). Strikingly, when overexpressed, only Ext-HR2-HR3 suppressed the septin mutant, similar to FL Bni5, whereas Ext-HR2-HR3 lacking any of the three motifs failed to suppress (Fig. 3 H). These results suggest that Ext-HR2-HR3 defines the structural and functional unit of Bni5 responsible for its binding to septin filaments and Elm1, as well as its regulation of septin functions in vivo.

### Bni5 regulates septin hourglass architecture and its timely remodeling into a double ring

Despite known genetic and physical interactions between Bni5 and septins (Booth et al., 2016; Booth et al., 2015; Finnigan et al., 2015; Lee et al., 2002; Patasi et al., 2015; Renz et al., 2013), the precise role of Bni5 in septin hourglass assembly and remodeling remains unclear.

**Figure 4.**
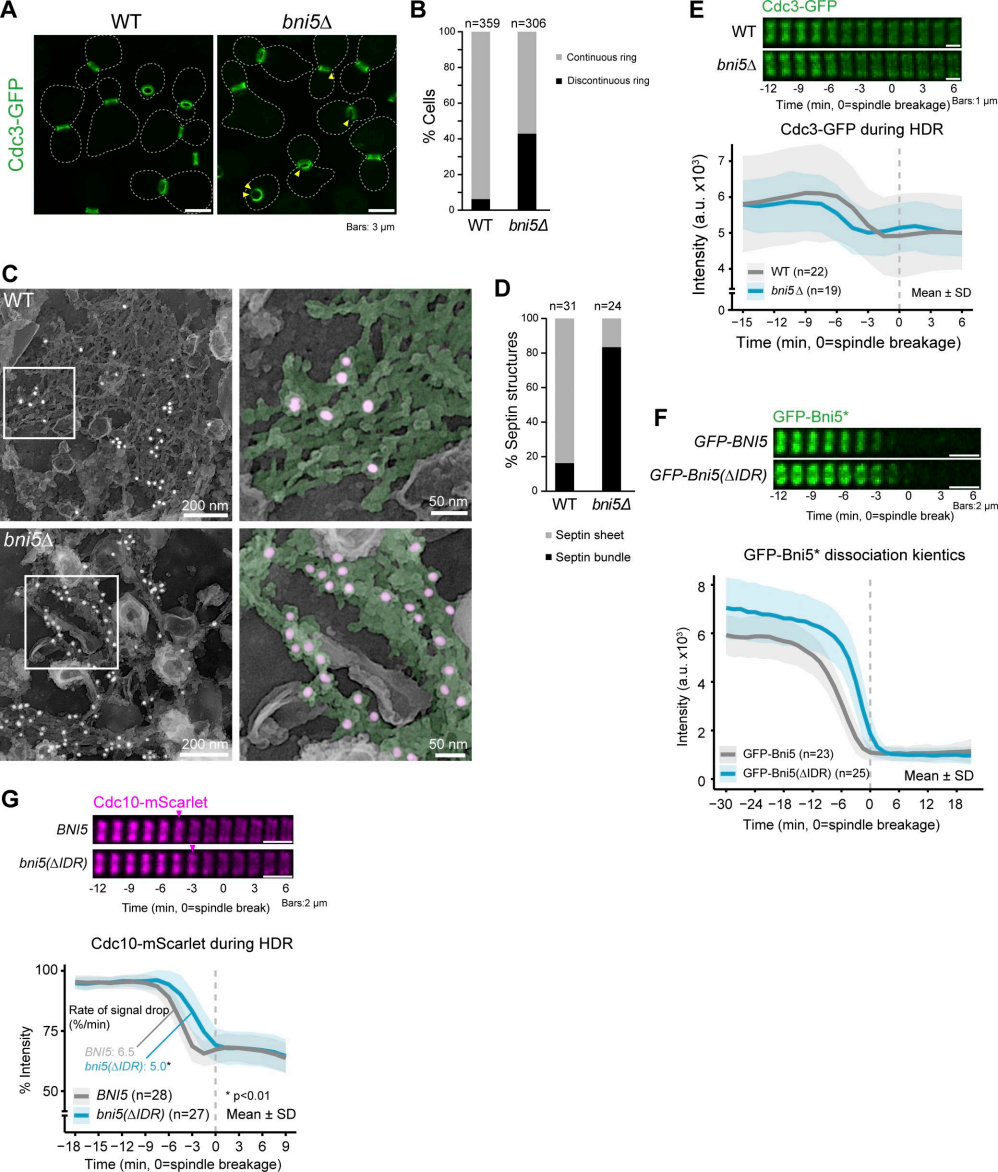
**Bni5 regulates septin hourglass architecture and its timely remodeling into a double ring. (A)** Representative fluorescent images of Cdc3-GFP in WT and *bni5Δ* cells, taken 1.5 h after release from a 2.5-h α-factor arrest. Arrowheads indicate discontinuities in the septin hourglass. Strains used: YEF9333 (*bar1Δ CDC3-GFP*) and YEF9767 (*bni5*Δ *bar1Δ CDC3-GFP*). **(B)** Quantification of septin hourglass morphology. Patterns of septin structures in the WT and *bni5Δ* cells were quantified from imaging data shown in A. *n* denotes the number of cells analyzed per strain. **(C)** Representative PREM images of Cdc3-labeled septin hourglass structures from WT and *bni5Δ* cells after α-factor arrest and release. Septin structures were immunogold-labeled using an 18-nm gold-conjugated secondary antibody and a primary anti-GFP antibody to detect Cdc3-GFP. The inset shows the magnified region of the white box. In the inset, septin filaments and gold particles are pseudo-colored green and pink, respectively. See also Fig. S3 A. **(D)** Quantification of septin structures in WT and *bni5Δ* cells from PREM imaging shown in C. *n* denotes the number of septin structures analyzed per strain. **(E)** Time-lapse analysis of the septin HDR transition in WT and *bni5Δ*. Top: Montages of Cdc3-GFP created from time-lapse series taken with 1.5-min intervals. Bottom: Cdc3-GFP localization at the bud neck during the HDR transition. *n* denotes the number of cells analyzed per strain. Bold lines and shaded bands represent the mean and SD, respectively. Strains used: YEF9180 (*CDC3-GFP mRuby2-TUB1*) and YEF9618 (*bni5*Δ *CDC3-GFP mRuby2-TUB1*). **(F)** Time-lapse analysis of Bni5 lacking its IDR. Montages and localization plots were created as in E. Asterisk indicates Bni5 variants. Strains used: YEF11546 (*mScarlet-TUB1 GFP-BNI5*) and YEF11553 [*bni5Δ mScarlet-TUB1 GFP-bni5(Δ41-305)*]. **(G)** Time-lapse analysis of the septin HDR transition in WT and *bni5(ΔIDR)* cells. Montages of Cdc10-mScarlet and corresponding localization plots were created as in E. P values were determined using a two-sided Mann–Whitney U test. Arrowheads indicate the onset of HDR transition. Strains used: YEF11772 [*CDC10-mScarlet Venus-TUB1 GFP-BNI5(FL)*] and YEF11773 [*CDC10-mScarlet Venus-TUB1 GFP-bni5(Δ41-305)*].

**Figure S3.**
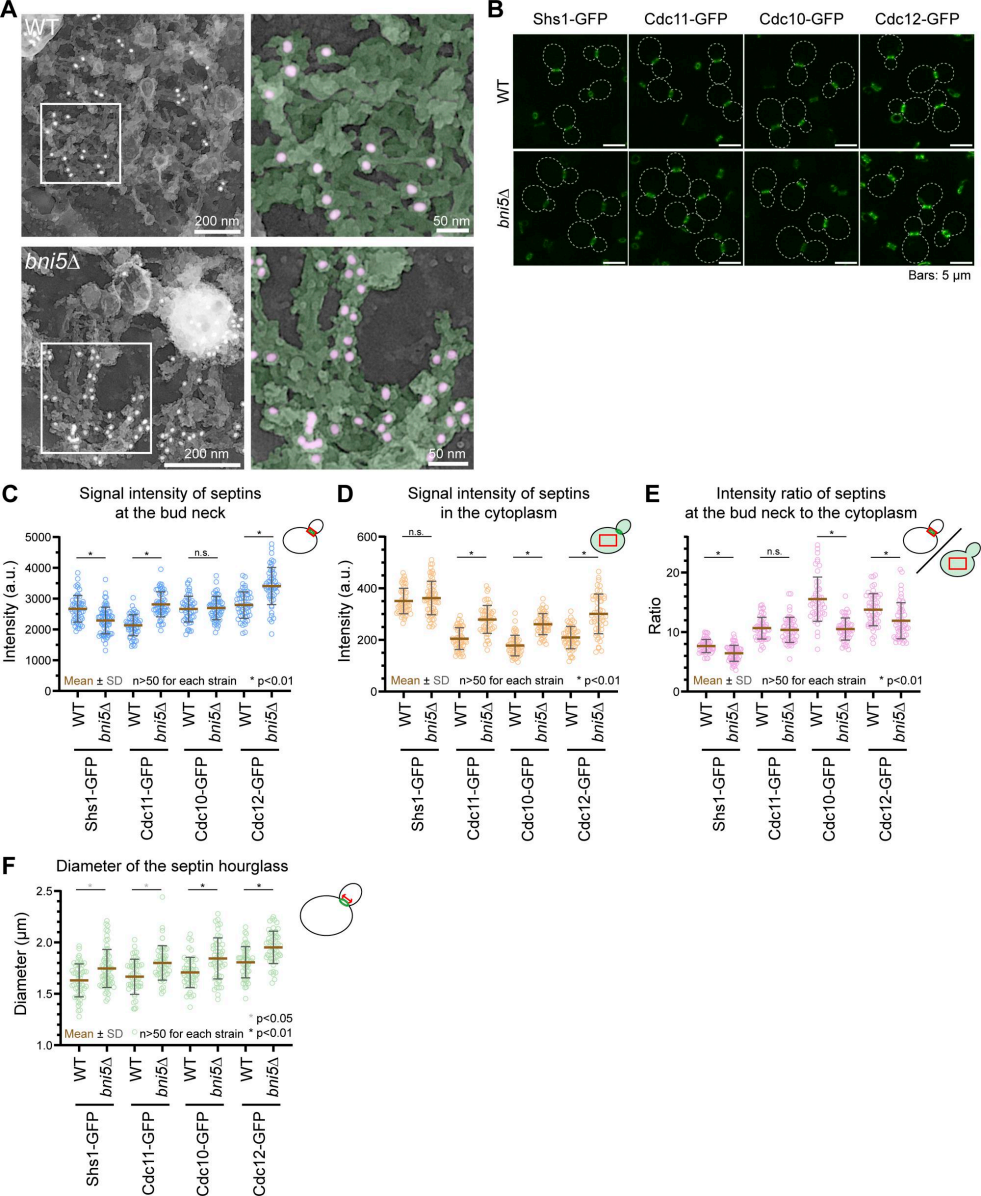
**Impact of *BNI5* deletion on the size, structure, and composition of the septin hourglass.** Related to Fig. 4. **(A)** Additional PREM images of WT and *bni5∆* cells. **(B)** Representative images showing localization of different GFP-tagged septin subunits in WT and *bni5∆* cells. Strains used: YEF11087 (*mRuby2-TUB1 SHS1-GFP*), YEF12141 (*bni5∆ mRuby2-TUB1 SHS1-GFP)*, YEF11754 (*mScarlet-TUB1 CDC11-GFP*), YEF12143 (*bni5∆ mScarlet-TUB1 CDC11-GFP*), YEF11753 (*mScarlet-TUB1 CDC10-GFP*), YEF12142 (*bni5∆ mScarlet-TUB1 CDC10-GFP*), YEF11755 (*mScarlet-TUB1 CDC12-GFP*), and YEF12144 (*bni5∆ mScarlet-TUB1 CDC10-GFP*). **(C–F)** Quantification of fluorescence signal from the indicated GFP-tagged septin subunits: (C) intensity at the bud neck; (D) intensity in the cytoplasm; (E) intensity ratio at the bud neck to the cytoplasm; (F) diameter of the septin hourglass. Strains used are the same as in B. Circles, brown bars, and gray bars represent individual data points, mean values, and SD, respectively. *n* denotes the number of cells analyzed per strain. P values were determined using a two-sided unpaired *t* test.

To investigate this, we first synchronized WT and *bni5Δ* cells expressing Cdc3-GFP at the early hourglass stage (Ong et al., 2014) and then examined septin architecture using instant structured illumination (iSIM) microscopy in live cells. Strikingly, 42.8% of *bni5Δ* cells formed a “discontinuous” ring (Fig. 4 A, arrowhead; and Fig. 4 B), while only 5.6% of WT cells showed this phenotype. These synchronized cells were also processed for the visualization of septin hourglass architecture using immunogold-labeling platinum-replica electron microscopy (PREM) (Ong et al., 2014). As expected (Marquardt et al., 2020; Ong et al., 2014), the hourglass in WT cells consisted primarily of “septin sheets” made of tightly packed septin filaments (Fig. 4, C and D; additional PREM images in Fig. S3 A). In contrast, *bni5Δ* cells exhibited hourglasses with “septin bundles” (Fig. 4, C and D; additional PREM images in Fig. S3 A). The loosened nature of the hourglass structures in mutant cells was further supported by their increased accessibility to immunogold labeling of Cdc3-GFP, compared with WT cells (Fig. 4 C). These results suggest that Bni5 may cross-link septin bundles into sheets, thereby stabilizing hourglass architecture at the bud neck and supporting a previous in vitro observation (Patasi et al., 2015).

To explore how Bni5 might affect hourglass architecture, we monitored the localization efficiency of individual septin subunits (GFP-tagged Shs1, Cdc11, Cdc10, or Cdc12) at the bud neck compared with the cytoplasm. We found that the average intensity of Shs1 at the bud neck decreased in *bni5Δ* cells, while the intensities of Cdc11 and Cdc12 increased, and Cdc10 remained unchanged (Fig. S3, B and C). Conversely, Shs1 intensity in the cytoplasm remained unaffected in *bni5Δ* cells, while all other septins increased (Fig. S3 D). The ratio of average intensity of Shs1 at the bud neck versus cytoplasm decreased, confirming a previous report (Schneider et al., 2013), while the ratios for Cdc10 and Cdc12 also decreased. In contrast, the Cdc11 ratio remained unchanged (Fig. S3 E). These findings suggest that Bni5 deletion reduces Shs1 at the bud neck relative to Cdc11. Given that an increased ratio of Cdc11-capped versus Shs1-capped septin octamers increases the ring size in vitro (Garcia et al., 2011), we measured the diameter of the septin hourglass and found it increased in *bni5Δ* cells, regardless of which septin subunit was used for measurement (Fig. S3 F). This provides the first in vivo evidence that Bni5 may regulate septin hourglass size by controlling the ratio of Shs1-/Cdc11-capped octamers at the bud neck.

The defective septin hourglass in *bni5Δ* cells may affect its subsequent remodeling into a double ring at cytokinesis onset. To test this, we examined the HDR transition using time-lapse microscopy and found it to be prematurely initiated in *bni5Δ* cells compared with WT (Fig. 4 E). Conversely, deleting the IDR in Bni5, which increased Bni5 localization at the bud neck and prolonged its association with the septin hourglass (Fig. 4 F), delayed the HDR transition (Fig. 4 G). Thus, the presence and duration of Bni5 at the bud neck control the precise timing of the HDR transition.

### Gin4-mediated phosphorylation of Bni5 promotes its dissociation from the septin hourglass

Elm1 interacts with both Bni5 and Gin4 and activates the kinase activity of Gin4 during mitosis (Asano et al., 2006; Marquardt et al., 2024; Patasi et al., 2015). Additionally, all three proteins are associated with the septin hourglass from bud emergence to the onset of cytokinesis. These observations suggest that Elm1 and/or Gin4 may phosphorylate Bni5 to regulate its interaction with septin filaments, thereby controlling its association with the septin hourglass.

To test whether Bni5 is a substrate of Elm1 or Gin4 in vivo, we conducted stable isotope labeling by aa in cell culture (SILAC) coupled with mass spectrometry on WT versus *elm1Δ* or *gin4Δ* strains to identify global kinase substrates (Baro et al., 2018; Mascaraque et al., 2013). This analysis revealed four definitive Elm1-dependent phosphorylation sites in Bni5 (**S70**, **S270**, **S346**, and **S349**) and three potential sites (T257, **S273**, and **S274**), inferred from phospho-peptides. In addition, eight definitive Gin4-dependent sites (S13, **S70**, **S270**, **S273**, **T274**, **S346**, **S349**, and S350) were identified, with six sites (bolded) shared between both kinases. These results support the hypothesis that Elm1 and Gin4 phosphorylate Bni5 in the same pathway.

**Figure S4.**
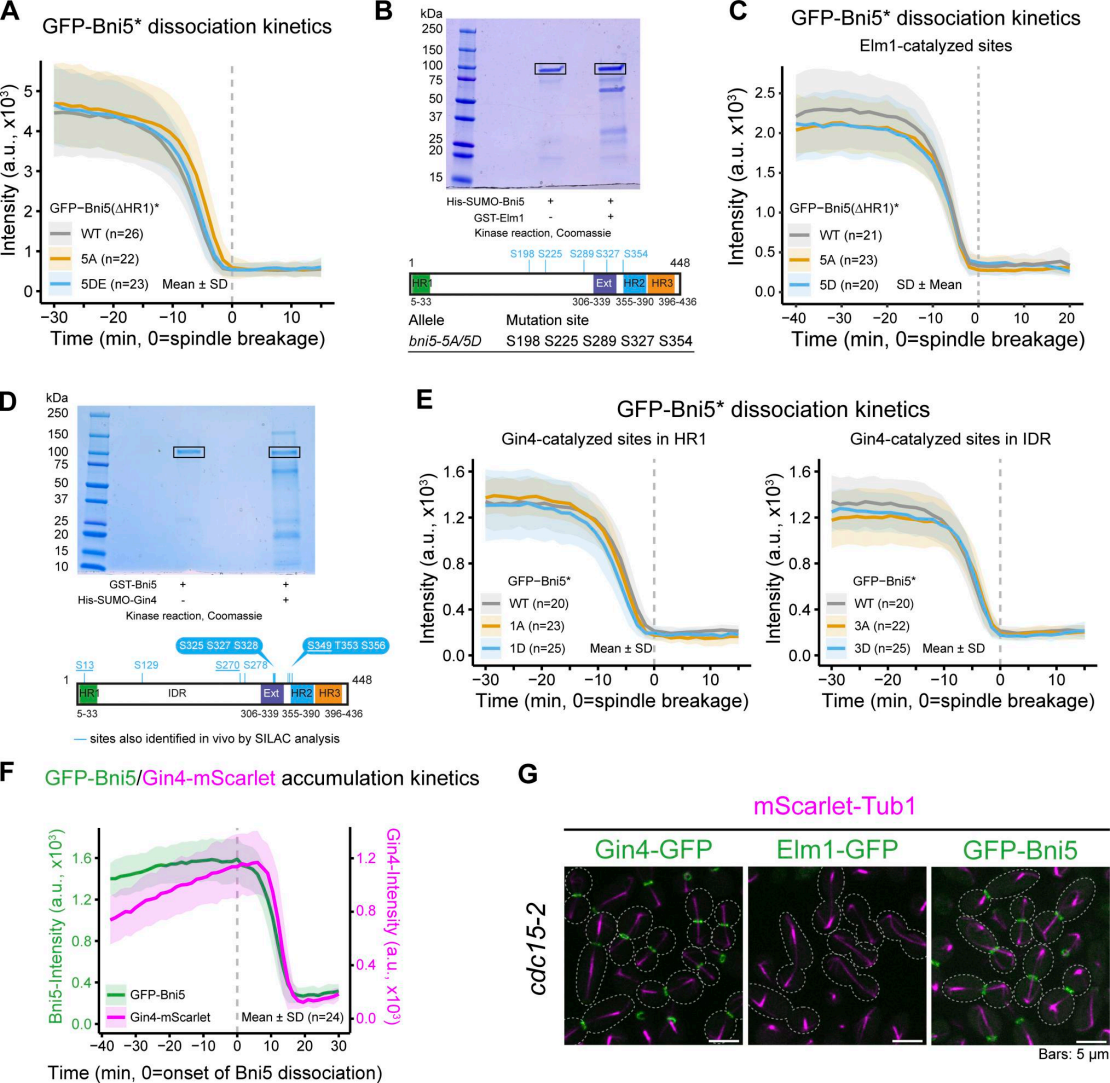
**Identification and functional analyses of both in vivo and in vitro Elm1- and Gin4-dependent phospho-sites in Bni5, and analysis of the differential effects of mitotic exit on the bud neck localization of Gin4, Elm1, and Bni5.** Related to Fig. 5. **(A)** Time-lapse imaging analysis of Elm1- and Gin4-dependent phosphorylation site mutants. *n* denotes the number of cells analyzed per strain. Bold lines and shaded bands represent the mean and SD, respectively. Asterisk indicates Bni5 variants. Strains used: YEF11600 [*mScarlet-TUB1 GFP-bni5(41-448)*], YEF12109 [*mScarlet-TUB1 GFP-bni5(41-448)-5A*], and YEF12112 [*mScarlet-TUB1 GFP-bni5(41-448)-5DE*]. **(B)** Top: In vitro kinase assay. Bni5 was incubated with or without GST-Elm1 in the presence of ATP, followed by SDS-PAGE and Coomassie Blue staining. Boxed regions were excised for mass spectrometry analysis. Bottom: Schematic of Bni5 protein showing domain boundaries and identified phosphorylation sites (blue vertical lines) determined by mass spectrometry. These sites were mutated to generate phospho-deficient (*bni5-5A*) and phospho-mimic (*bni5-5D*) alleles. **(C)** Time-lapse imaging analysis of Elm1-catalyzed phosphorylation site mutants. *n* denotes the number of cells analyzed per strain. Bold lines and shaded bands represent the mean and SD, respectively. Asterisk indicates Bni5 variants. Strains used: YEF11600 [*mScarlet-TUB1 GFP-bni5(41-448)*], YEF12240 [*mScarlet-TUB1 GFP-bni5(41-448)-5A*], and YEF12241 [*mScarlet-TUB1 GFP-bni5(41-448)-5D*]. **(D)** Top: In vitro kinase assay. Bni5 was incubated with or without 6xHis-SUMO-Gin4 in the presence of ATP, followed by SDS-PAGE and Coomassie Blue staining. Boxed regions were excised for mass spectrometry. Bottom: Schematic of Bni5 as described above (B). **(E)** Time-lapse imaging analysis of Gin4-catalyzed phosphorylation site mutants. *n* denotes the number of cells analyzed per strain. Bold lines and shaded bands represent the mean and SD, respectively. Asterisk indicates Bni5 variants. Strains used: YEF11546 [*mScarlet-TUB1 GFP-BNI5*], YEF12440 [*mScarlet-TUB1 GFP-bni5-1A*], YEF12441 [*mScarlet-TUB1 GFP-bni5-1D*], YEF12442 [*mScarlet-TUB1 GFP-bni5-3A*], and YEF12441 [*mScarlet-TUB1 GFP-bni5-3D*]. **(F)** Time-lapse imaging of GFP-Bni5 and Gin4-mScarlet during cytokinesis. *n* denotes the number of cells analyzed per strain. Bold lines and shaded bands represent the mean and SD, respectively. The strain used: YEF12427 (*bni5∆ GFP-BNI5 GIN4-mScarlet*). **(G)** Representative images of GFP-Bni5, Gin4-GFP, and Elm1-GFP in *cdc15-2* cells at the restrictive temperature. Cells were grown to exponential growth phase at 25°C in YM-1 medium and shifted to 37°C for 2.5 h before imaging. Strains used: YEF12227 [*cdc15-2mScarlet-TUB1 GFP-BNI5(FL)*], YEF12402 (*cdc15-2mScarlet-TUB1 GIN4-GFP*), and YEF12412 (*cdc15-2 mScarlet-TUB1ELM1-GFP*).

Interestingly, four of the Elm1- and/or Gin4-dependent sites (S270, T274, S346, and S350) were previously studied without identifying the responsible kinase(s) (Nam et al., 2007a; Nam et al., 2007b). Using C-terminally tagged Bni5, those studies showed that a phospho-deficient version (Bni5-4A-GFP, where all four residues were changed to alanine) caused premature dissociation of Bni5 from the bud neck, whereas a phospho-mimic version was not analyzed (Nam et al., 2007b). Since C-terminally tagged Bni5-GFP is known to be defective in septin binding, we reanalyzed these four sites along with S349 using functional, N-terminally tagged GFP-Bni5. Both the phospho-deficient (Bni5*-5A) and phospho-mimic (Bni5*-5DE) variants localized to the bud neck as efficiently as WT Bni5 lacking HR1 (to eliminate potential complications from Myo1 binding; see next section). Bni5* denotes the HR1-less version. However, GFP-Bni5*-5A exhibited a slight delay in dissociation from the bud neck (Fig. S4 A), contrasting previous results using Bni5-4A-GFP (Nam et al., 2007b).

While SILAC analysis provides a broad view of phospho-proteome landscapes, it does not establish direct kinase-substrate relationships and may miss key phosphorylation sites due to protein abundance or transient phosphorylation. To determine whether Bni5 is a direct substrate of Elm1 and Gin4, we performed in vitro kinase assays using GST-Elm1 and 6xHis-SUMO-Bni5 or 6xHis-SUMO-Gin4 and GST-Bni5, followed by liquid chromatography–mass spectrometry/mass spectrometry (LCMS/MS). We identified five Elm1-phosphorylated sites in Bni5 (S198, S225, S289, S327, and S354), none of which matched the Elm1-dependent sites identified in vivo (Fig. S4 B). Furthermore, neither the phospho-deficient (Bni5*-5A) nor the phospho-mimic (Bni5*-5D) versions showed obvious defects in localization (Fig. S4 C), consistent with in vitro findings that Elm1-mediated phosphorylation does not affect the binding of Bni5 to septin filaments (Patasi et al., 2015).

**Figure 5.**
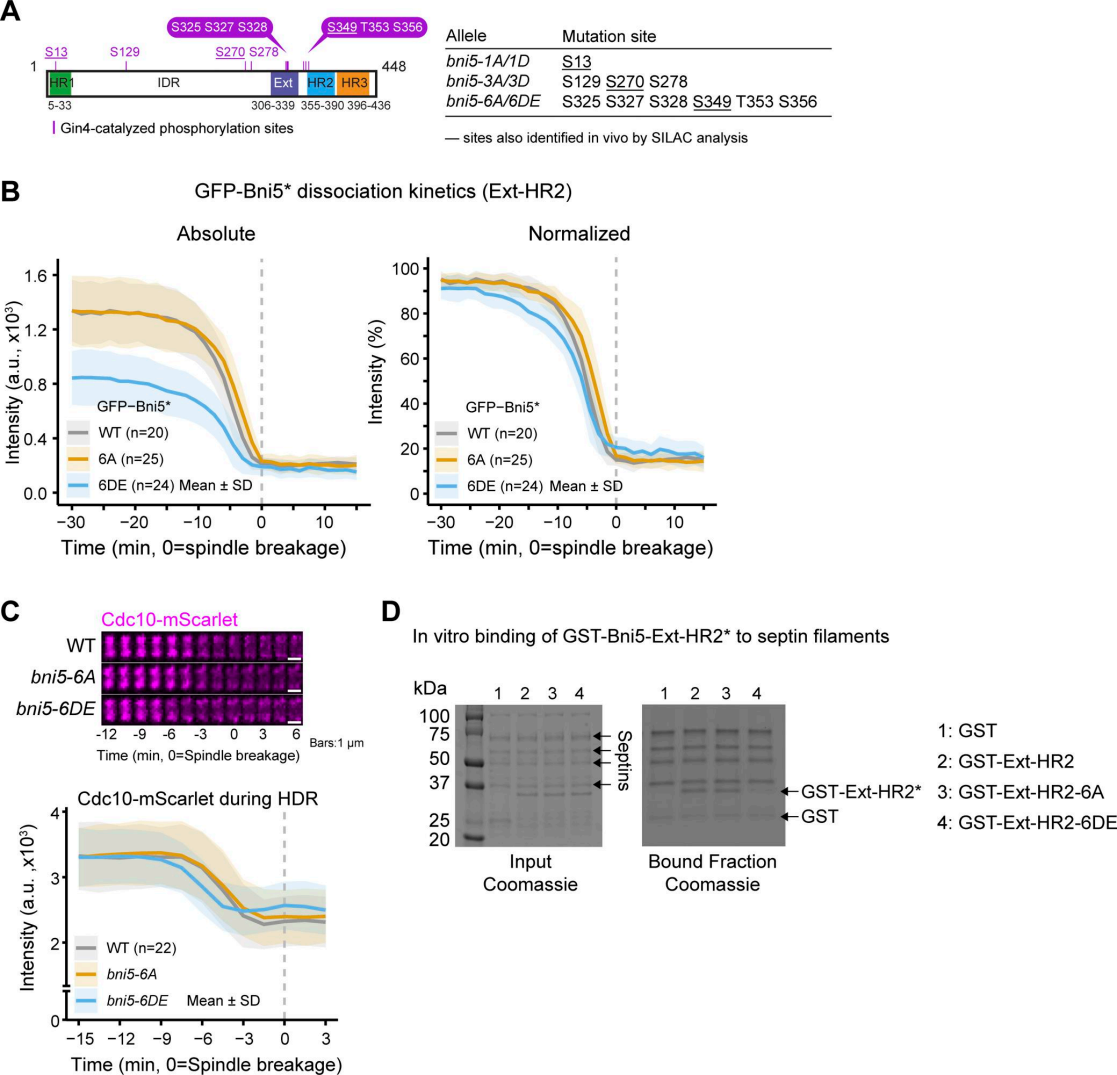
**Gin4-catalyzed phosphorylation of Bni5 promotes its dissociation from the septin hourglass. (A)** Left: Diagram showing Gin4-catalyzed phosphorylation sites in Bni5 identified by in vitro kinase assay. Right: Summary of phospho-deficient and -mimic *bni5* alleles and their respective mutation sites. **(B)** Time-lapse analysis of GFP-tagged Bni5 phospho-mutants at the bud neck. Left: Dissociation kinetics of GFP-Bni5 and its phospho-mutants were quantified over time. Absolute values (left) and normalized data (right) are shown. *n* denotes the number of cells analyzed per strain. Bold lines and shaded bands represent the mean and SD, respectively. Asterisk indicates variants of Bni5. Strains used: YEF11546 [*mScarlet-TUB1 GFP-BNI5(FL)*], YEF12444 (*mScarlet-TUB1 GFP-bni5-6A*), and YEF12445 (*mScarlet-TUB1 GFP-bni5-6DE*). **(C)** Time-lapse analysis of the septin HDR transition in WT and Bni5 phospho-mutants. Top: Montages of Cdc10-mScarlet created from time-lapse series taken with 1.5-min intervals. Bottom: The dissociation kinetics of Cdc10-mScarlet during the HDR transition in indicated strains are shown. *n* denotes the number of cells analyzed per strain. Bold lines and shaded bands represent the mean and SD, respectively. Strains used: YEF11772 (*CDC10-mScarlet Venus-TUB1 GFP-BNI5(FL)-6A*), YEF12456 (*CDC10-mScarlet Venus-TUB1 GFP-BNI5(FL)-6A*), and YEF12457 (*CDC10-mScarlet Venus-TUB1 GFP-BNI5(FL)-6DE*). **(D)** In vitro binding of Bni5-Ext-HR2 variants to septin filaments. Left: SDS-PAGE stained with Coomassie blue, showing purified proteins used as input. Input septin complexes contained five septins: Cdc10, His6-Cdc12, Cdc3, Cdc11, and Shs1. Right: Results of binding assays, showing Coomassie blue stained of GST-tagged proteins co-sedimented with septin filaments by ultracentrifugation. Asterisk indicates variants of Bni5-Ext-HR2. kDa = kiloDalton. A representative result from three independent experiments is shown. Source data are available for this figure: SourceData F5.

In the in vitro kinase assays, we also identified 10 Gin4-phosphorylated sites in Bni5 (**S13**, S129, **S270**, S278, S325, S327, S328, **S349**, T353, and S356), with 3 (bolded) matching the Gin4-dependent sites identified in vivo (Fig. 5 A and Fig. S4 D). These sites were grouped based on their locations: S13 in HR1 (Myo1-binding site); S129, S270, and S278 in IDR; and S325, S327, S328, S349, T353, and S356 in Ext-HR2. Phospho-deficient (Bni5-1A and Bni5-3A) or phospho-mimic (Bni5-1D and Bni5-3D) mutations in HR1 or IDR had no obvious effect on the accumulation kinetics of Bni5 at the bud neck (Fig. S4 E). However, phospho-mimic mutations in the septin-binding region (Bni5-6DE) caused a 37% reduction in signal at 30 min before spindle breakage and led to earlier dissociation from the bud neck compared with WT, while phospho-deficient mutations (Bni5-6A) showed no significant defects (Fig. 5 B). These results suggest that Gin4 regulates Bni5 dissociation from the bud neck by phosphorylating the septin-binding site.

Both Elm1 and Gin4 dissociate from the bud neck just before or during the MEN-triggered HDR transition, with Elm1 leaving slightly earlier than Gin4 (Marquardt et al., 2024). We observed that the maximal fluorescence intensity of Gin4 coincided with the onset of rapid Bni5 dissociation (Fig. S4 F), suggesting that Gin4-mediated phosphorylation promotes Bni5 release at the HDR transition. Supporting this model, the onset of the HDR transition, marked by a drop in Cdc10-mScarlet signal, occurred ∼1.5 min earlier in *bni5-6DE* mutant cells than in WT (Fig. 5 C), mirroring the timing observed in *bni5Δ* cells (Fig. 4 E). Furthermore, the phospho-mimic Ext-HR2 fragment (Bni5-Ext-HR2-6DE), but not the phospho-deficient counterpart (Bni5-Ext-HR2-6A), showed markedly reduced binding to septin filaments (Fig. 5 D), providing additional evidence for a phosphorylation-dependent mechanism regulating Bni5 dissociation.

In the temperature-sensitive MEN mutant, *cdc15-2*, at the nonpermissive temperature, we observed that Gin4, but not Elm1, remained at the bud neck (Fig. S4 G). Similarly, Bni5 also failed to dissociate from the bud neck in the MEN mutant (Fig. S4 G), confirming a previous report (Lee et al., 2002). Taken together, our results suggest that Bni5 dissociation from the bud neck is largely dictated by the MEN through a mechanism that remains to be determined, while Gin4 facilitates this process by phosphorylating Bni5 at its septin filament-binding site.

### Bni5 interacts with the tail of myosin-II via a helical region at its N terminus

**Figure 6.**
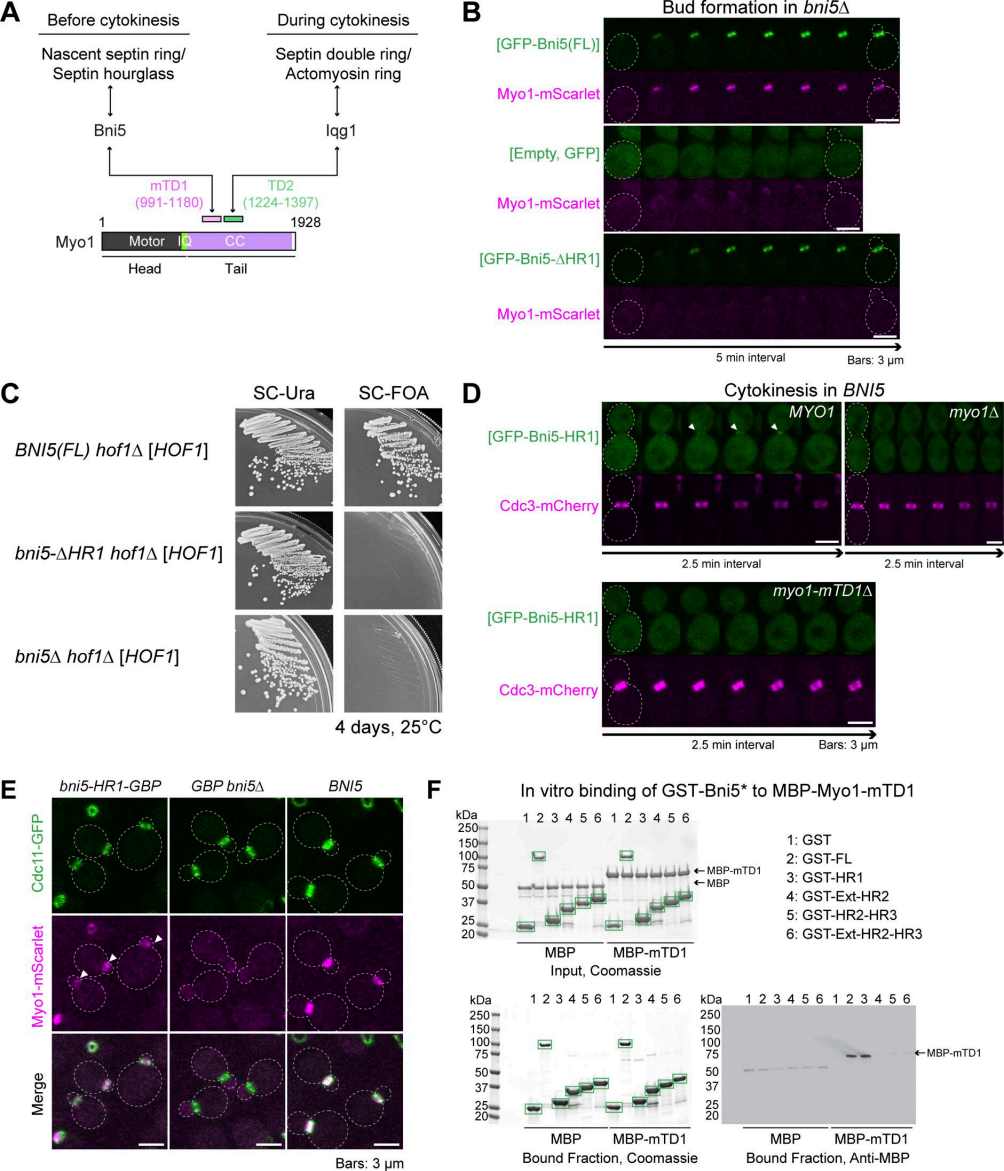
**Bni5 recruits Myo1 to the septin hourglass via HR1. (A)** Diagram of putative and demonstrated interactions among Bni5, Myo1, and Iqg1. The mTD1 and targeting domain 2 (TD2) in the tail of Myo1 mediate its direct or indirect interactions with Bni5 and Iqg1, respectively (Fang et al., 2010). Motor, IQ, and CC refer to the motor domain, IQ motifs, and coiled-coil domain, respectively. **(B)** Myo1 accumulation at the budding site depends on the HR1 domain of Bni5. Montages were created from selected frames of time-lapse series taken with 2.5-min intervals. Strains used: YEF11029 (*bni5Δ MYO1-mScarlet* [*GFP-BNI5(FL)*]), YEF11033 (*bni5Δ MYO1-mScarlet* [*GFP*]), and YEF11027 (*bni5Δ MYO1-mScarlet* [*GFP-bni5(41-448)*]). **(C)** The HR1-coding sequence of *BNI5* is essential for suppression of the *bni5Δ hof1Δ* synthetic lethality. Growth on 5-FOA plates tested the ability of GFP-Bni5-ΔHR1 to support viability in the absence of *HOF1*. Strains used: YEF12280 (*hof1Δ GFP-BNI5(FL)* [*HOF1-GFP URA3 CEN*]), YEF12267 (*hof1Δ GFP-bni5(41-448)* [*HOF1-GFP URA3 CEN*]), and YEF12330 (*hof1Δ bni5Δ* [*HOF1-GFP URA3 CEN*]). **(D)** Localization of HR1 at the bud neck during cytokinesis depends on Myo1 and its mTD1. Montages of GFP-Bni5-HR1 in WT, *myo1Δ*, and *myo1-mTD1Δ* backgrounds were created from time-lapse series taken with 2.5-min intervals. Strains used: YEF11071 (*CDC3-mCherry* [*GFP-bni5(1–40)*]), YEF11114 (*myo1Δ CDC3-mCherry* [*GFP-bni5(1–40)*]), and YEF11757 (*myo1-mTD1Δ CDC3-mCherry* [*GFP-bni5(1–40)*]). **(E)** Artificial tethering of HR1 to the septin hourglass restores Myo1 localization at the bud neck in the absence of FL Bni5. Arrowheads indicate the Myo1-mScarlet signal at the bud neck mediated by HR1-GBP and Cdc11-GFP interaction. Strains used: YEF11212 (*CDC11-GFP MYO1-mScarlet bni5(1–40)-GBP*) (*bni5(1–40)-GBP* replaced the endogenous *BNI5 ORF*), YEF11211 (*CDC11-GFP MYO1-mScarlet bni5Δ::GBP*) (*GBP* replaced the endogenous *BNI5 ORF*), and YEF11088 (*CDC11-GFP MYO1-mScarlet*). **(F)** The HR1 domain of Bni5 interacts directly with the mTD1 of Myo1 in vitro. Top left: SDS-PAGE stained with Coomassie blue depicting amounts of each indicated purified protein used as the input for the in vitro–binding assay. Bottom left: In vitro–binding assay results (Coomassie blue–stained gel) for the indicated GST-tagged proteins bound to glutathione resin and their ability to pull down MBP-mTD1. Green boxes represent GST and GST-tagged Bni5 fragments described top right. Right: Immunoblotted membrane with antibody against MBP; kDa = kiloDalton. This experiment was repeated three times, and a representative immunoblot is shown. Source data are available for this figure: SourceData F6.

Bni5 mediates the association of Myo1 with the nascent septin ring and septin hourglass before cytokinesis, while Iqg1, the sole IQGAP in *S. cerevisiae* essential for AMR assembly (Boyne et al., 2000; Epp and Chant, 1997; Fang et al., 2010; Lippincott and Li, 1998; Wang et al., 2023), maintains Myo1 and drives AMR constriction between the septin double ring during cytokinesis (Fig. 6 A) (Fang et al., 2010). We have previously shown that Bni5- and Iqg1-mediated Myo1 localization occurs through targeting domains, minimal targeting domain 1 (mTD1) and TD2, in the Myo1 tail (Fig. 6 A) (Fang et al., 2010). However, the region of Bni5 responsible for its interaction with Myo1 remains unknown.

Using the same strategy employed to identify binding sites for septin filaments and Elm1, we identified the Myo1-binding site in Bni5. As expected, GFP-Bni5 and Myo1-mScarlet co-localized as a ring at the bud neck during bud formation, whereas in *bni5Δ* cells, the neck localization of Myo1-mScarlet was abolished (Fig. 6 B) (Fang et al., 2010). Strikingly, in *bni5Δ* cells expressing GFP-Bni5-ΔHR1, Myo1-mScarlet failed to localize to the bud neck, similar to the *bni5Δ* phenotype, despite the presence of the mutant Bni5 at the bud neck (Fig. 6 B). Furthermore, *bni5-ΔHR1* failed to rescue the synthetical lethality observed between *bni5Δ* and the deletion of *HOF1*, an F-BAR protein known to interact with septins and play a role in cytokinesis (Fig. 6 C) (Lee et al., 2002; Meitinger et al., 2013; Oh et al., 2013). These results indicate that HR1 is essential for Bni5-mediated Myo1 localization and the shared role of Bni5 and Hof1 in Myo1- and/or septin-mediated functions.

Reciprocally, we found that GFP-HR1 localized to the bud neck during cytokinesis, and this localization depended on Myo1, specifically on the mTD1 domain in the Myo1 tail (Fig. 6 D). Strikingly, forced tethering of HR1 to septins (Cdc11-GFP) using the GFP/GBP (GFP-binding protein) system (Rothbauer et al., 2008) led to the association of Myo1-mScarlet with septins (Fig. 6 E). Finally, recombinant Bni5-HR1 and Myo1-mTD1 were shown to interact directly in vitro (Fig. 6 F). Together, these data demonstrate that Bni5 recruits Myo1 to the bud neck by interacting with mTD1 in the Myo1 tail via its HR1 domain.

### Bni5 mediates the role of myosin-II in retrograde actin cable flow before cytokinesis

**Figure 7.**
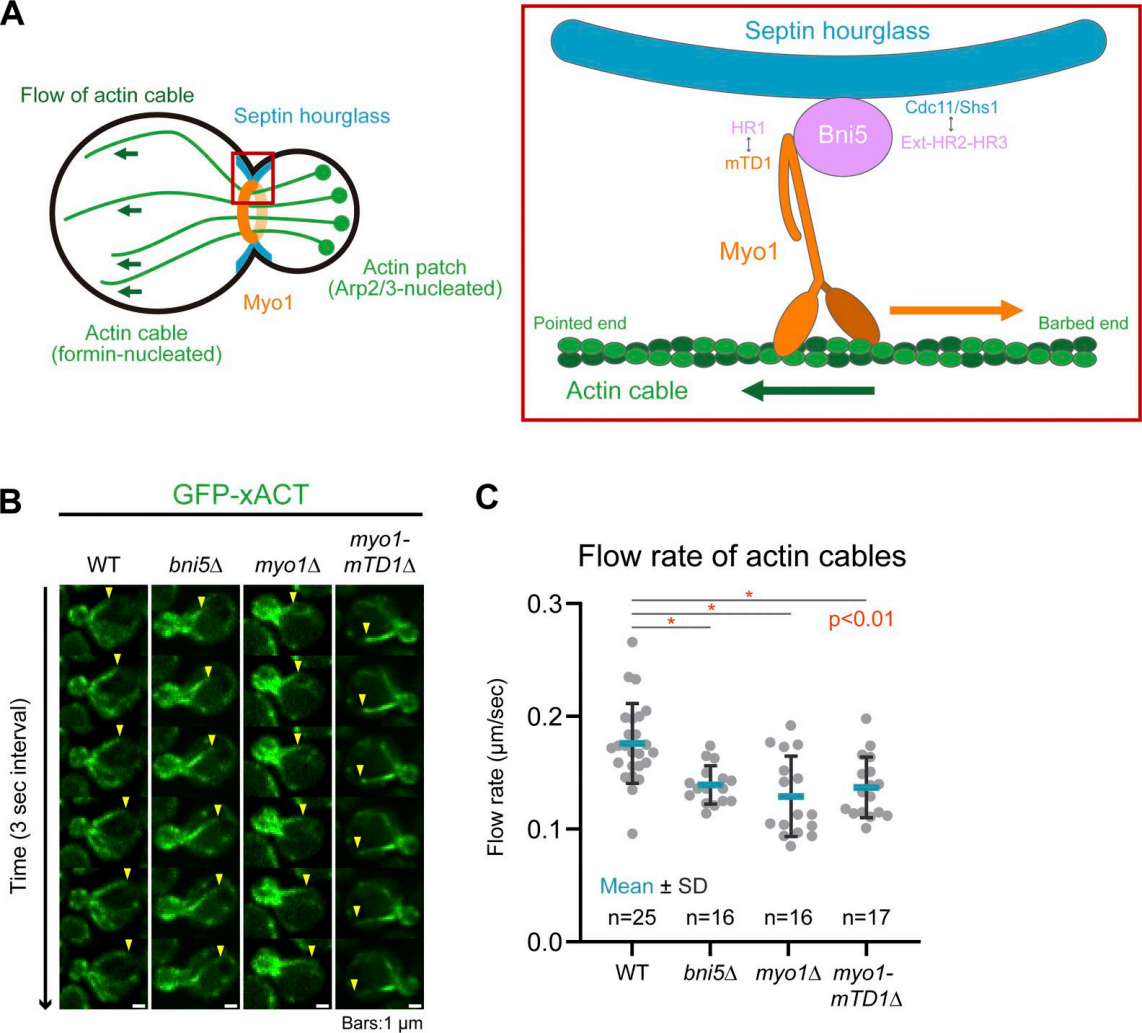
**Bni5-mediated localization of Myo1 at the division site before cytokinesis facilitates retrograde actin cable flow. (A)** Diagram of Myo1-facilitated retrograde flow of actin cables before cytokinesis. See details in the text. **(B)** Time-lapse analysis of retrograde actin cable flow in *bni5Δ* and *myo1* mutants. Montages showing dynamics of GFP-xACT–labeled actin cables were created from selected frames of a time-lapse series taken with 1-s intervals. Arrowheads indicate the distal ends of elongating actin cables. The *myo1Δ* strain (YEF10170) was grown on a 5-FOA to eliminate the *MYO1* cover plasmid prior to imaging. Strains used: YEF10201 (*GFP-xACT*), YEF10173 (*bni5Δ GFP-xACT*), YEF10170 (*myo1Δ GFP-xACT*), and YEF11489 (*myo1-mTD1Δ GFP-xACT*). **(C)** Quantification of actin cable flow rate from the time-lapse data shown in B. Gray circles, blue bars, and black bars represent individual data points, mean values, and SD, respectively. P values were determined using a two-sided Mann–Whitney U test.

Polarized actin cables and actin patches are nucleated by formins and the Arp2/3 complex at the bud cortex to mediate exocytosis and endocytosis, respectively, during bud growth (Bi and Park, 2012; Moseley and Goode, 2006; Pruyne et al., 2002; Sagot et al., 2002; Winter et al., 1999a; Winter et al., 1999b) (Fig. 7 A). The actin cables also undergo retrograde flow, facilitated by Myo1, which requires both its motor activity and its localization at the bud neck (Huckaba et al., 2006) (Fig. 7 A). This retrograde flow has been implicated in the asymmetric segregation of mitochondrial fitness between the mother and daughter/bud compartments during bud growth (Higuchi et al., 2013). While Bni5 is essential for Myo1 localization at the bud neck before anaphase (Fang et al., 2010), its role in retrograde flow has not yet been experimentally demonstrated.

To investigate this, we used GFP-xACT, a recently developed actin cable probe derived from budding yeast Ecm25 (Duan et al., 2021), to visualize and track actin cable movement through time-lapse analysis (Fig. 7 B). As expected, *MYO1* deletion reduced the retrograde flow rate by 27% (Fig. 7 C). Deletion of *BNI5* similarly reduced the flow rate by 21%. Strikingly, deletion of the Bni5-binding site in Myo1 (i.e., *myo1*-*mTD1Δ*) caused a near-identical (22%) decrease, comparable with the *BNI5* deletion (Fig. 7, B and C). These results suggest that Bni5 interacts with the mTD1 of Myo1 to facilitate retrograde actin cable flow before cytokinesis.

### Bni5 enhances the robustness of cytokinesis by increasing myosin-II at the division site

**Figure 8.**
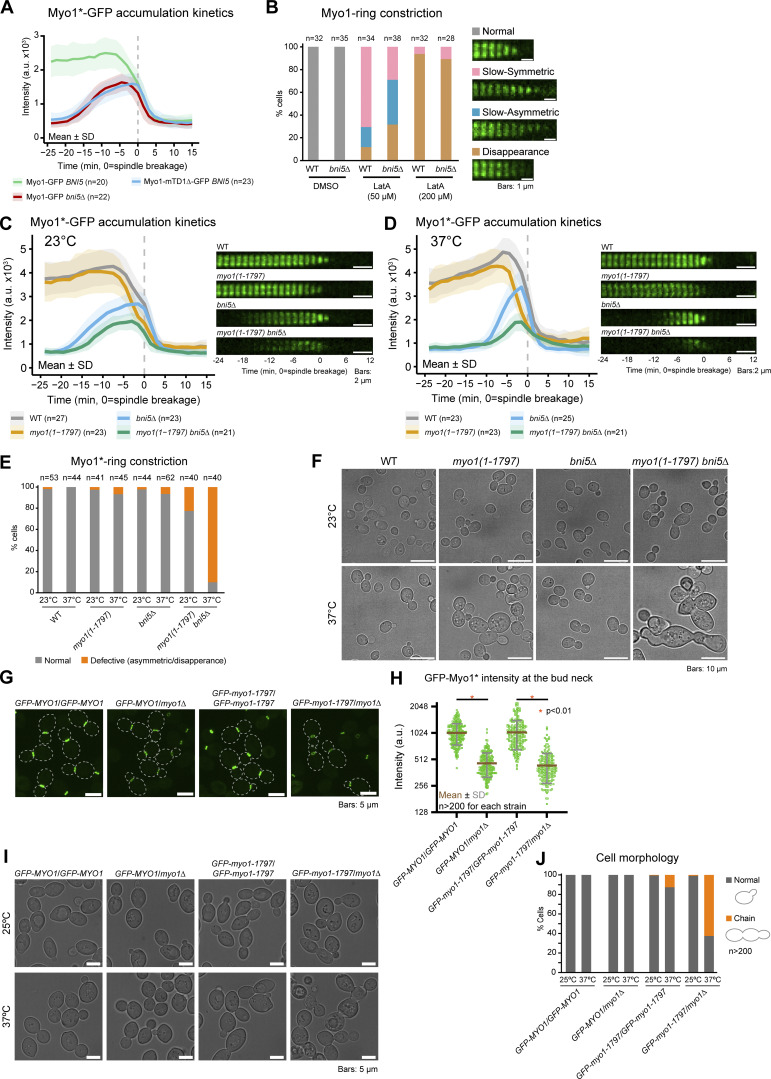
**Bni5-mediated recruitment of Myo1 to the division site before cytokinesis endows the AMR robustness against perturbation during cytokinesis. (A)** The Bni5–Myo1 interaction is essential for high levels of Myo1 accumulation at the bud neck prior to cytokinesis. *n* denotes the number of cells analyzed per strain. Bold lines and shaded bands represent the mean and SD, respectively. Asterisk indicates variants of Myo1. Strains used: YEF11441 (*MYO1-GFP mScarlet-TUB1*), YEF11442 (*myo1-mTD1Δ-GFP mScarlet-TUB1*), and YEF11443 (*bni5Δ MYO1-GFP mScarlet-TUB1*). **(B)** Disruption of F-actin compromises AMR constriction in *bni5Δ* cells. AMR (Myo1-GFP) constriction in the presence of moderate (50 µM) or high (200 µM) concentrations of LatA was scored based on the indicated categories from time-lapse series taken with 2-min intervals. *n* denotes the number of cells analyzed per strain. Strains used: YEF11385 (*MYO1-GFP mScarlet-TUB1*) and YEF11443. **(C and D)** Synthetic effects of *bni5Δ* and Myo1 tail truncation on Myo1 accumulation at the bud neck at 23°C and 37°C. Left: Accumulation kinetics of FL and tail-truncated Myo1 at the bud neck in WT and *bni5Δ* background were quantified from time-lapse series taken at 23°C (C) or 37°C (D) with 1.5-min intervals. *n* denotes the number of cells analyzed per strain. Bold lines and shaded bands represent the mean and SD, respectively. Right: Montages of indicated proteins at the bud neck were created from selected frames. Strains used: YEF11529 (*GFP-MYO1 mScarlet-TUB1*), YEF11524 [*GFP-myo1(1-1797) mScarlet-TUB1*], YEF11601 (*bni5Δ GFP-MYO1 mScarlet-TUB1*), and YEF11530 [*bni5Δ GFP-myo1(1-1797) mScarlet-TUB1)*]. **(E)** Quantification of AMR constriction phenotype in the indicated strains, based on imaging data from C and D. *n* denotes the number of cells analyzed per strain. Asterisk indicates variants of Myo1. **(F)** Cell morphology defects in *bni5Δ* and *myo1* tail-truncation mutants. Cells were cultured in YM-1 medium at 25°C or 37°C for 24 h before imaging. Strains used are the same as in C and D. **(G)** Representative images of GFP-Myo1 and GFP-Myo1-1797 in homozygous and hemizygous diploid strains. Strains used: YEF6611 (*GFP-MYO1/GFP-MYO1*), YEF12297 (*GFP-MYO1/myo1Δ*), YEF6608 [*GFP-myo1(1-1797)/GFP-myo1(1-1797)*], and YEF12293 [*GFP-myo1(1-1797)/myo1Δ*]. **(H)** Quantification of GFP-Myo1 and GFP-Myo1-1797 fluorescence intensities from images in G. Green squares, brown bars, and gray bars represent individual data points, mean values, and SD, respectively. Black asterisk indicates variants of Myo1, and red asterisks indicate P < 0.01. P values were determined using a two-sided Mann–Whitney U test. **(I and J)** Cell morphology of the GFP-Myo1 and GFP-Myo1-1797 homozygous and hemizygous strains. **(I)** Representative images of cells of indicated strains were acquired as F. **(J)** Quantification of cell morphology. *n* denotes the number of cells analyzed per strain. Strains used are the same as in G.

**Figure S5.**
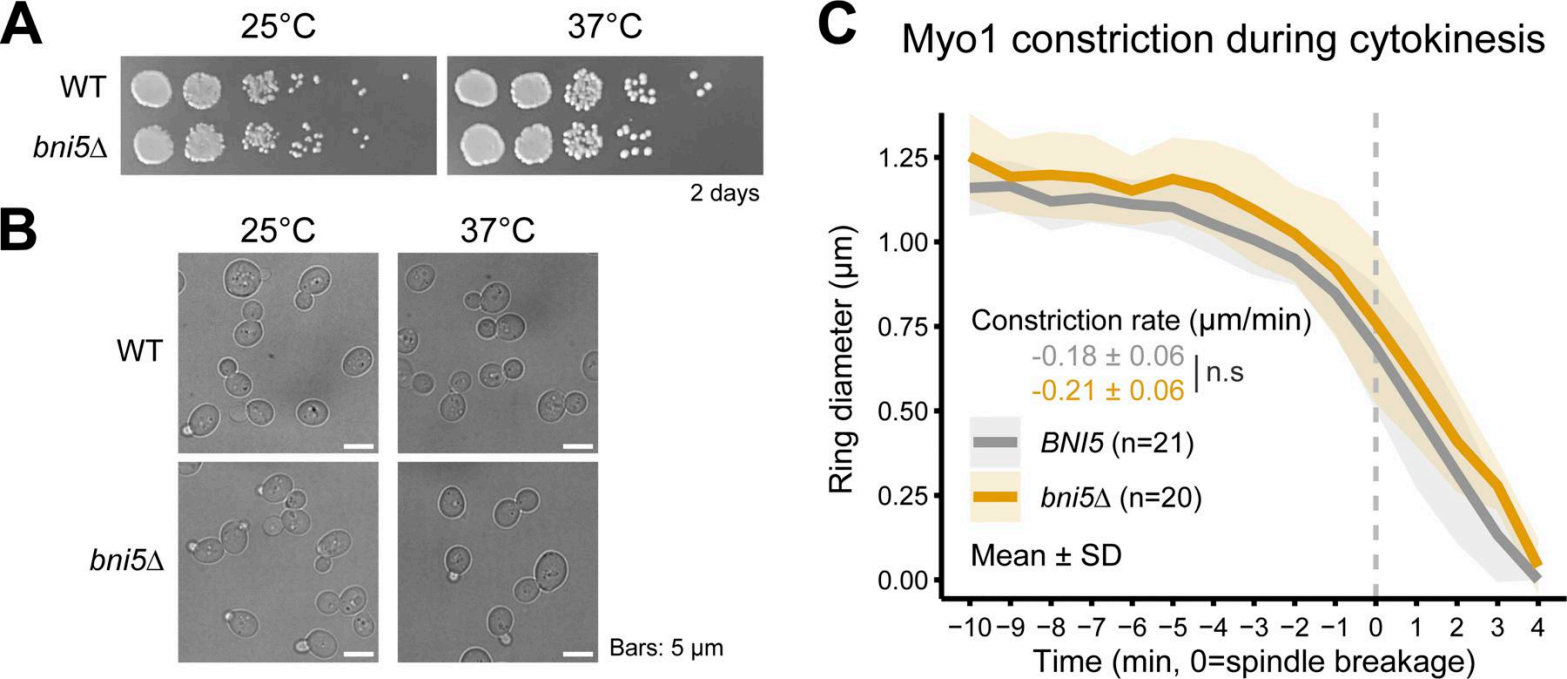
**Effect of *BNI5* deletion on cell growth, morphology, and Myo1 constriction at 25°C and 37°C.** Related to Fig. 8. **(A)** Growth of *bni5Δ* cells on YPD plates. Spot assay results were documented after 2 days of incubation at 25°C or 37°C. Strains used: YEF473A (WT) and YEF9654 (*bni5Δ*). **(B)** Cell morphology of *bni5Δ* cells. Cells were cultured in YM-1 at 25°C or 37°C for 24 h prior to imaging. Strains used are the same as in A. **(C)** Constriction of Myo1 ring during cytokinesis in WT and *bni5Δ* cells. The temporal changes in the diameter of the Myo1 ring were quantified from time-lapse series taken with 1-min intervals. *n* denotes the number of cells analyzed per strain. Bold lines and shaded bands represent the mean and SD, respectively. P values were determined using a two-sided unpaired *t* test. Strains used: YEF11385 (*MYO1-GFP mScarlet-TUB1*) and YEF11443 (*bni5Δ MYO1-GFP mScarlet-TUB1*).

Bni5 and Iqg1 mediate Myo1 localization at the bud neck before and during cytokinesis (Fang et al., 2010). We found that, in the absence of Bni5, the peak intensity of Myo1 at the bud neck was reduced by 35% (2,496.5 ± 466.0 versus 1,635.1 ± 262.2, P < 0.01, Fig. 8 A). A similar reduction was also observed for Myo1 lacking the Bni5-binding site (Myo1-mTD1Δ) (Fig. 8 A). These results indicate that Bni5 is required to maintain a higher level of Myo1 at the bud neck at the onset of cytokinesis. Surprisingly, we did not observe any growth or morphological defects in *bni5Δ* cells at either 25°C or 37°C (Fig. S5, A and B), nor did we find any significant difference in Myo1 constriction during cytokinesis (Fig. S5 C). This contrasts, for reasons unknown, with a previous report showing that deletion of *BNI5* causes a partially penetrant defect in cytokinesis and cell morphology at high temperatures (Lee et al., 2002).

We hypothesize that the Bni5-mediated increase in Myo1 at the bud neck may confer robustness to cytokinesis, buffering it against chemical and genetic perturbations. To test this, we treated cells with latrunculin A (LatA) (Ayscough et al., 1997). A high dose of LatA (200 µM) abolished actin filaments in both WT and *bni5Δ* cells, causing Myo1 disappearance without constriction during cytokinesis (Fig. 8 B) (Bi et al., 1998; Okada et al., 2021b). A lower dosage (50 µM) caused a mild phenotype in most of WT cells (71% slow symmetric constriction, Fig. 8 B). The remaining cells exhibited more severe phenotypes, such as slow asymmetric constriction (18%) or disappearance without constriction (12%, Fig. 8 B). In comparison, *bni5Δ* cells showed a more severe response to the same dosage (39% slow asymmetric and 32% disappearance, Fig. 8 B). These results suggest that, in the absence of Bni5, the AMR is more susceptible to F-actin perturbation.

We also examined the role of Bni5 in cytokinesis when Myo1 was compromised. Previous work showed that Myo1 lacking a small tail region (aa 1798–1928) became more mobile at the bud neck during cytokinesis, as revealed by FRAP (Wloka et al., 2013). Time-lapse analysis of cells at 23°C showed that single mutants of *myo1-1797* and *bni5Δ* lost peak Myo1 levels by 5% and 37%, respectively, compared with WT, whereas the double mutant (*myo1-1797 bni5Δ*) lost Myo1 levels by 54% (Fig. 8 C). A similar, though more profound, effect was observed under temperature stress at 37°C. The single mutants, *myo1-1797* and *bni5Δ*, and the double mutant, *myo1-1797 bni5Δ*, lost peak Myo1 levels by 13%, 31%, and 61%, respectively (Fig. 8 D), with 90% of the double mutant cells exhibiting severe phenotypes (asymmetric constriction or disappearance of the ring, Fig. 8 E). These cells also showed marked defects in cytokinesis (Fig. 8 F). These results suggest that, in the absence of Bni5, cytokinesis with genetically perturbed Myo1 is more susceptible to temperature stress.

Since Bni5 regulates not only Myo1 accumulation but also septin hourglass architecture and remodeling, both of which could, in principle, affect cytokinesis, we sought to determine whether the Bni5-mediated increase of Myo1 at the bud neck is responsible for the cytokinesis defects observed above. To test this, we reduced Myo1 levels by modulating gene copy number, generating homozygotes and hemizygotes expressing either *GFP-MYO1* or *GFP-myo1-1797*. As expected, GFP-Myo1 and GFP-Myo1-1797 levels at the bud neck were reduced by 55.0% and 58.1%, respectively, in hemizygotes compared with homozygotes (Fig. 8, G and H). Similar to *myo1-1797 bni5Δ* cells, the *GFP-myo1-1797* hemizygotes exhibited pronounced defects in cytokinesis at 37°C, but not at 25°C. These findings suggest that Myo1 levels at bud neck become critical for cytokinesis under temperature stress when Myo1 is genetically perturbed.

Taken together, these data suggest that Bni5-mediated increase of Myo1 at the bud neck enhances the robustness of cytokinesis, buffering against chemical and genetic perturbations, particularly under stress conditions.

## Discussion

Our study defines the precise timing and molecular mechanisms by which Bni5 couples myosin-II to septins during the cell cycle. We also uncover the roles of Bni5 in promoting the assembly of septin hourglasses, their subsequent remodeling into double rings, and Myo1-mediated retrograde actin cable flow and cytokinesis robustness. These findings establish Bni5 as one of the best-defined linkers between septins and myosin-II, both at the molecular and functional levels.

### Mechanisms of Bni5 interactions with septin filaments and Elm1 and their implications in septin hourglass assembly and remodeling

**Figure 9.**
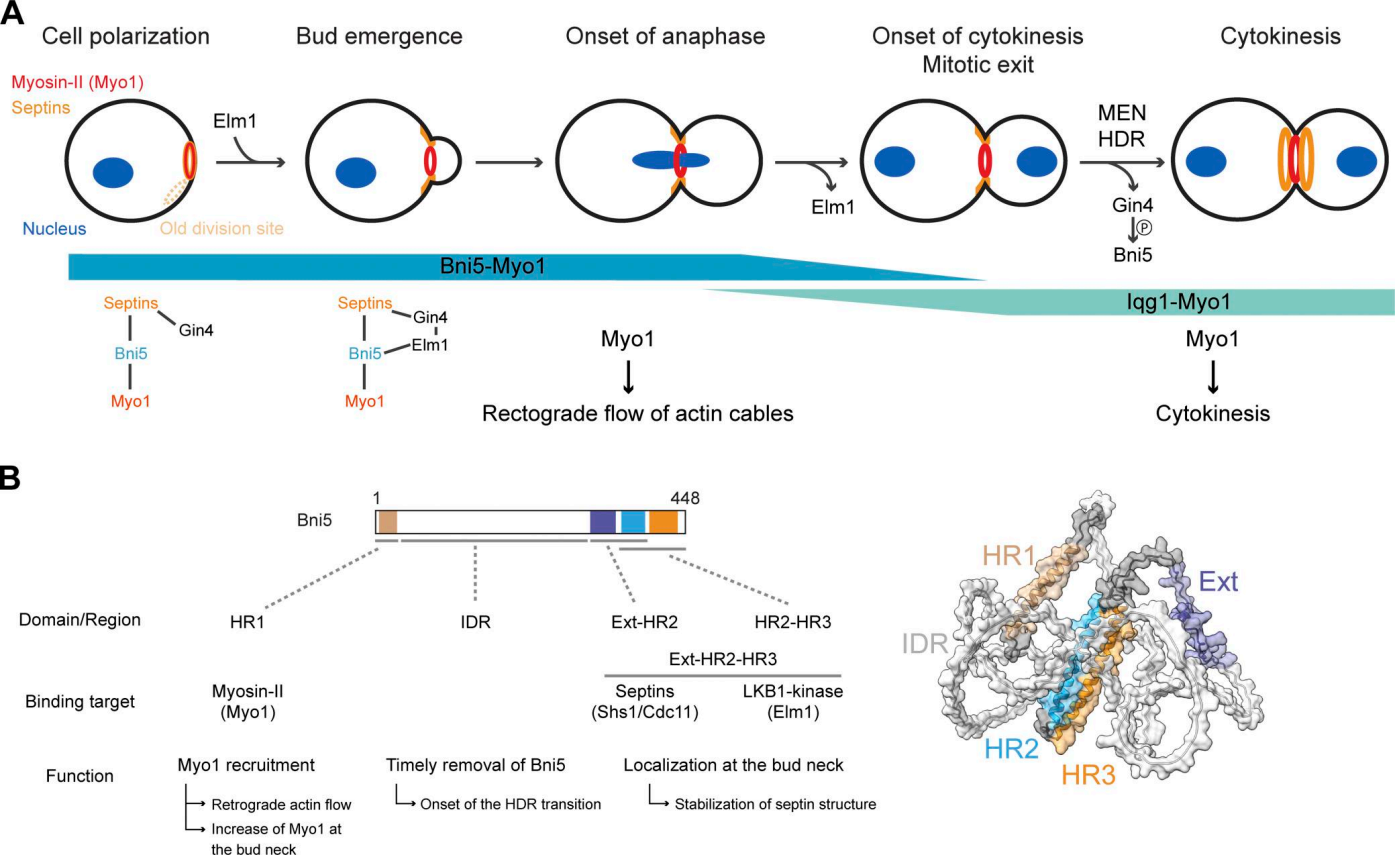
**Bni5 coordinates cell cycle–specific regulation and function of Myo1 and septins through distinct domains and binding partners. (A)** Diagram of the biphasic recruitment of Myo1 to the bud neck and its distinct roles during the cell cycle. **(B)** Diagram of the distinct domains of Bni5, their binding partners, and associated functions. See text for details.

We found that Bni5 links Myo1 to septins from cell polarization to the onset of cytokinesis (Fig. 9 A). This coupling is facilitated by the septin-associated kinase Elm1, which tethers Bni5 to the septin hourglass from bud emergence to the onset of cytokinesis (Fig. 9 A). This tethering mechanism becomes essential when the direct Bni5–septin interaction is disrupted. Gin4, another septin-associated kinase, and Elm1 exert mutual control during the cell cycle (Marquardt et al., 2024). In late G1, Gin4 associates with the nascent septin ring at the site of cell polarization, alongside Bni5 and Myo1 (Fig. 9 A) (Marquardt et al., 2024). Approximately 12 min later, Elm1 joins this complex at bud emergence, with its localization depending on Gin4 (Fig. 9 A) (Marquardt et al., 2024). During mitosis, Elm1 activates Gin4 and regulates its dissociation from the bud neck (Asano et al., 2006; Marquardt et al., 2024; Mortensen et al., 2002). Shortly before or during the MEN-triggered HDR transition at the onset of cytokinesis, all three proteins, Elm1, Gin4, and Bni5, dissociate from the bud neck, with Elm1 leaving earlier than Gin4 and Bni5 (Fig. 9 A) (Bouquin et al., 2000; Caydasi et al., 2010; Fang et al., 2010; Lee et al., 2002; Lippincott et al., 2001; Longtine et al., 1998a; Marquardt et al., 2024; Moore et al., 2010; Patasi et al., 2015; Schneider et al., 2013). Consistent with this timing, the dissociation of Gin4 and Bni5, but not Elm1, depends on MEN activation. These findings provide new insights into the molecular coordination between Bni5, septins, Myo1, and the Gin4 and Elm1 kinases, establishing a clearer framework for understanding the functions and mechanisms of Bni5 in cell division.

We also found that Bni5 interacts with septin filaments and Elm1 through overlapping regions, Ext-HR2 and HR2-HR3, at its C terminus. Importantly, only the fragment containing all three motifs (Ext-HR2-HR3) fully accounts for Bni5 localization at the bud neck and can suppress the growth defect of a septin mutant, similar to the FL protein. Thus, the Ext-HR2-HR3 fragment acts as a structural and functional unit mediating the role of Bni5 in septin interaction and function.

Using synchronized cells in combination with super-resolution iSIM or immunogold-labeling PREM, we found that Bni5 promotes septin hourglass assembly, at least in part, by bundling septin filaments into a tight array, supporting previous in vitro observations (Patasi et al., 2015). We also observed that deletion of *BNI5* leads to a decrease in the ratio of Shs1/Cdc11 at the bud neck and an enlargement of the septin hourglass. This provides in vivo evidence supporting the in vitro observation that decreasing the ratio of Shs1-capped/Cdc11-capped octamers in a polymerization reaction results in larger septin rings (Garcia et al., 2011). Given the role of Bni5 in the septin hourglass assembly and stability (Schneider et al., 2013), it is not surprising that, in the absence of Bni5, the HDR transition occurs prematurely. This is likely due to the destabilization of the hourglass, which is part of the HDR transition process (Chen et al., 2020; DeMay et al., 2011; Ong et al., 2014). Conversely, deletion of the IDR in Bni5 prolongs its association with the septin hourglass, delaying the HDR transition. Thus, timely dissociation of Bni5 from the septin hourglass acts as a “permissive signal” that allows the HDR transition to proceed on schedule. Our analysis further suggests that the timing and association of Bni5 with the septin hourglass are mediated by the IDR and the Ext-HR2-HR3 regions, respectively.

Additionally, our SILAC analyses indicate that Bni5 is phosphorylated in vivo in an Elm1- and Gin4-dependent manner, with several phosphorylated residues shared by both kinases. We further demonstrate that Bni5 is a direct substrate of Elm1 and Gin4 in vitro; however, only the phosphorylation sites catalyzed by Gin4 overlap with the Elm1- and Gin4-dependent sites identified in vivo. These findings are consistent with previous observations that Elm1 functions upstream of Gin4 during mitosis (Asano et al., 2006; Marquardt et al., 2024). We also found that a phospho-mimic, but not a phospho-deficient, version of the six Gin4-phosphorylated sites within the Ext-HR2 region reduced Bni5’s interaction with septin filaments, resulting in its earlier dissociation from the bud neck and a premature HDR transition. Together, these results suggest that Elm1 and Gin4 act in a kinase cascade to promote Bni5 dissociation from the septin hourglass by phosphorylating its septin filament–binding site, thereby facilitating the remodeling of the hourglass into a double ring.

### Bni5-mediated anchoring mechanism for myosin-II and its implication in retrograde actin cable flow

Bni5 and Iqg1 are known to mediate Myo1 localization at the bud neck before and during cytokinesis, with overlapping activity from the onset of anaphase to the onset of cytokinesis (Fig. 9 A) (Fang et al., 2010). This overlapping period coincides with the formation of the actin ring in a Myo1- and Iqg1-dependent manner (Bi et al., 1998; Epp and Chant, 1997; Lippincott and Li, 1998), leading to the assembly of an AMR that constricts after the HDR transition (Fig. 9 A). However, the function of Myo1 at the bud neck before anaphase remains unknown.

Myo1 facilitates retrograde actin cable flow during bud growth, a process that depends on its localization at the bud neck and its motor activity (Huckaba et al., 2006). This retrograde flow is thought to contribute to the asymmetric inheritance of mitochondrial fitness (Higuchi et al., 2013). Given that Bni5 serves as the sole linker between Myo1 and the septin hourglass before anaphase (Fang et al., 2010) and directly interacts with the mTD1 region in the Myo1 tail via its HR1 domain (Fig. 9 B), Bni5 likely mediates this role. Consistent with this, we found that deletion of Bni5 reduces retrograde actin flow to the same extent as deletion of the mTD1 region in the Myo1 tail, suggesting that Bni5 mediates the role of Myo1 in this process by binding to the mTD1 region.

### Bni5-mediated increase of myosin-II at the division site and its implication in cytokinesis robustness

We found that deletion of Bni5 reduces the peak level of Myo1 at the bud neck by 35% at the onset of cytokinesis. However, this decrease alone does not lead to clear defects in cytokinesis. In contrast, *bni5Δ* cells treated with a low dose of LatA, which partially depolymerizes actin filaments, exhibit significant defects in AMR constriction. Additionally, *bni5Δ* cells with a C-terminally truncated *myo1* allele show clear defects in both AMR constriction and cytokinesis, particularly at 37°C. Furthermore, hemizygotes carrying the C-terminally truncated *myo1* allele (but not the WT allele) display clear defects in cytokinesis at 37°C. Collectively, these findings suggest that Bni5-mediated Myo1 accumulation at the division site is essential for cytokinesis robustness, buffering it against chemical, genetic, and temperature perturbations.

The need to maintain precise myosin-II levels is observed across diverse systems. In fission yeast, increased expression of Myo2 enhances AMR assembly (Stark et al., 2010). Similarly, in mice, NM-IIB becomes essential for blood vessel formation when a single copy of the gene encoding NM-IIA is ablated in endothelial cells (Ma et al., 2020). These examples underscore the critical role of tightly regulating myosin-II levels in various cellular processes, including cytokinesis.

### Bni5 and anillin: Septin–myosin-II linkers in budding yeast and animal cells

The evolution of molecular machinery from a common ancestor has led to both conserved and specialized functions in different lineages. This principle is well illustrated by Bni5. Our findings indicate that Bni5 links Myo1 to the septin hourglass, promoting retrograde actin cable flow before anaphase (Fig. 9 A). This function likely reflects a specific adaptation within *Saccharomycetaceae* species, including *S. cerevisiae*, where polarized actin cables and their retrograde flow are critical for polarized cell growth and asymmetric inheritance of organelles (Bi and Park, 2012; Bretscher, 2003; Higuchi et al., 2013; Huckaba et al., 2006). Bni5 is not just a passive linker—it actively promotes septin hourglass assembly, stability, and remodeling before cytokinesis (Fig. 9 A). It also facilitates AMR assembly on the septin hourglass from the onset of anaphase to cytokinesis (Fig. 9 A). This role is supported by our observation that Bni5 increases Myo1 accumulation at the bud neck, which fine-tunes actin ring assembly (Fang et al., 2010), as Myo1 is essential for this process (Bi et al., 1998). While *bni5Δ* cells show normal Myo1 constriction under our standard imaging condition (cells immobilized on a glass-bottom chamber dish with liquid medium), they exhibit asymmetric (26.7%) or partial (40.0%) Myo1 constriction under more stressful imaging conditions (cells immobilized on a thin agarose pad with growth medium) (Fang et al., 2010). These findings suggest that Bni5 couples Myo1 to the septin hourglass, coordinating their localization, assembly, and remodeling to influence both retrograde flow and cytokinesis.

Despite lacking sequence similarity, anillins in metazoans share molecular and functional features with Bni5. Like Bni5, anillin binds both myosin-II and septin filaments via distinct domains in its N and C termini, respectively (Oegema et al., 2000; Piekny and Maddox, 2010; Straight et al., 2005). Anillin also regulates cytokinesis by coordinating septin recruitment; organization; and the assembly, stability, and contractility of the AMR, which includes myosin-II and F-actin at the division site (D’Avino et al., 2008; Field et al., 2005; Garno et al., 2021; Henson et al., 2024). However, anillin has evolved additional domains that bind to F-actin and RhoA, allowing it to regulate more complex cellular signals and functions, reflecting the greater functional demands of animal cell division (Carim and Hickson, 2023; Field and Alberts, 1995; Kinoshita et al., 2002; Piekny and Glotzer, 2008; Piekny and Maddox, 2010).

## Materials and methods

### Yeast media and culture conditions

Standard culture media and genetic techniques were used (Guthrie and Fink, 1991). Yeast strains were routinely grown at 25°C in synthetic complete (SC) minimal medium lacking specific aa(s) and/or uracil or in rich medium (YM-1) (Lillie and Pringle, 1980). For overexpression of *BNI5* alleles, methionine-depleted SC medium was used. A 20 mM stock solution of LatA (in DMSO, FUJIFILM Wako Pure Chemical) was diluted into media at the indicated final concentrations.

### Constructions of strains

New strains were constructed either by integrating a plasmid carrying a modified gene at a genomic locus or by transferring a deletion or tagged allele of a gene from a plasmid or from one strain to another via PCR amplification and yeast transformation (Lee et al., 2013; Longtine et al., 1998b) (see footnotes in Table S1).

### Primers and plasmids

All plasmids and PCR primers used in this study are listed in Tables S2 and S3, respectively. All PCR primers and synthesized DNA were purchased from Integrated DNA Technologies. All new constructs were validated by sequencing performed at the DNA Sequencing Facility, University of Pennsylvania, Philadelphia, PA, USA. Plasmids pAG25 (Goldstein and McCusker, 1999), pET-His6-Sumo-Bni5, pFA6a-link-ymScarlet-I-CaURA, pFA6a-link-yomApple-GBP-CaURA (Marquardt et al., 2020), pET-His6-Sumo-Gin4 and pGEX-4T1-Elm1^WT^ (Marquardt et al., 2024), YIp128-CDC3-GFP (Gao et al., 2007), pFA6a-link-yoEGFP-SpHIS5 and pFA6a-link-yoTagRFP-T-CaURA3 (Lee et al., 2013), pMAL-MYO1-mTD1 (Fang et al., 2010), pRG205MX and pRG206MX (Gnügge et al., 2016), proHIS3-ymScarlet-I-TUB1-tTUB1-HPH (Ghanegolmohammadi et al., 2021), pRS316-N-MYO1-GFP (Caviston et al., 2003), and pUG36-GFP-Ecm25-xACT(536–588 aa) (Duan et al., 2021) were described previously.

The following plasmids were kindly provided by the indicated colleagues: bWL715 and bWL722 (Markus et al., 2015) (Wei-Lih Lee, Dartmouth College, Hanover, NH, USA); pCOLA-Duet-[His less]-Shs1 (Garcia et al., 2011), pMVB128, and pMVB133 (Versele et al., 2004) (Jeremy Thorner, University of California, Berkeley, CA, USA); pFA6a-URA3-KanMX6 (Onishi et al., 2013) (John Pringle, Stanford University, Stanford, CA, USA); pUG36 (Johannes H. Hegemann, Heinrich Heine University Düsseldorf, Düsseldorf, Germany); and YCp50-MYO1 (Susan Brown, University of Michigan, Ann Arbor, MI, USA).

The following plasmids were constructed in this study: To generate pET-His6-Sumo-Elm1_FL_, pET-His6-Sumo-Elm1_1-420_, and pET-His6-Sumo-Elm1_421-640_, a DNA fragment containing *ELM1(FL)*, *elm1(1–420)*, and *elm1(421–640)*, respectively, were amplified by PCR using plasmid pUG36-ELM1(FL), pUG36-ELM1(1–420), or pUG36-ELM1(421–640) (lab stocks) as templates, with the primer pairs Elm1-FL-F/Elm1-FL-R, Elm1-N-F/Elm1-N-R, or Elm1-C-F/Elm1-C-R, respectively. The PCR products were subcloned into BamHI- and SspI-digested pET-His6-Sumo-TEV-LIC (#29659; Addgene) using In-Fusion HD cloning kit (Takara).

To construct pFA6a-link-GBP-CaURA3, a ∼0.4-kb DNA fragment containing *GBP-6xHis* was amplified by PCR from pRS315-INN1-C2-mApple-GBP-6xHis (lab stock) using primers GBP-F and GBP-R. The fragment was used to replace the ∼0.7-kb PacI-AscI region of pFA6a-link-yoTagRFP-T-CaURA3 using the In-Fusion cloning kit (Takara).

To construct pGEX-4T-1-BNI5(1–448), pGEX-4T-1-BNI5(1–40), pGEX-4T-1-BNI5(306–393), pGEX-4T-1-BNI5(c), and pGEX-4T-1-BNI5(306–448), BamHI-XhoI fragments of the corresponding *BNI5* coding regions were excised from pUG36-based plasmids series (Table S2) and ligated into the BamHI-XhoI–digested pGEX-4T-1-SSO1 (lab stock) using T4 DNA ligase (New England Biolabs). To construct pGEX-4T-1-BNI5(306–393)-6A and pGEX-4T-1-BNI5(306–393)-6DE, ∼0.3-kb *bni5* fragments were amplified by PCR using pRG205MX-proBNI5-yEGFP-BNI5(FL)-6A and pRG205MX-proBNI5-yEGFP-BNI5(FL)-6DE as templates, with primers P1532 and P1535. PCR products were digested with HindIII and BamHI and subcloned into HindIII-BamHI–digested pGEX-4T-1-BNI5(340–448) using T4 DNA ligase.

To construct pRG205MX-proBNI5-yEGFP, a DNA fragment containing the 800-bp promoter region of *BNI5, yEGFP*, and a linker sequence (SRTSGSPGL) was amplified by PCR using genomic DNA from strain YEF10276 (Marquardt et al., 2020) and primers P1581 and P1582. The product was subcloned into SacI- and XbaI-digested pRG205MX using in-fusion kit.

To generate pRG205MX-proBNI5-yEGFP-BNI5 fusion constructs expressing FL or truncated *BNI5* (1–40, 306–339, 306–448, 340–448, 41–448, and Δ41-305), DNA fragments were excised from the corresponding pUG36 plasmids (Table S2) using EagI and BamHI and subcloned into EagI-BamHI–digested pRG205MX-proBNI5-yEGFP using T4 DNA ligase. A ∼3.3-kb SacI-PvuII fragment containing *proBNI5-yEGFP-BNI5(FL)* from pRG205MX-proBNI5-yEGFP-BNI5 was ligated into the SacI-PvuII–digested pRG206MX to construct pRG206MX-proBNI5-yEGFP-BNI5(FL).

To generate pRG205MX-proBNI5-yEGFP-BNI5 fusion constructs containing phosphorylation-site mutations, one- or two-step site-directed mutagenesis and in-fusion cloning were performed. The 5A/5DE mutations (S270, T274, S346, S349, and S350) were introduced in two sequential PCR steps. In the first step, pRG205MX-proBNI5-yEGFP-BNI5(41–448) was used as the template for amplification using primer pairs P1755/P1756 and P1753/P1754 to introduce mutations at two residues at S270 and T274. Following plasmid purification and sequence verification, a second round of mutagenesis was performed using primer pairs P1795/P1796 and P1793/P1794 to introduce mutations at S346, S349, and S350. The 5A/5D mutations (S198, S225, S289, S327, and S354) were introduced by in-fusion cloning of synthesized DNA fragments containing the respective mutations (purchased from IDT) into pRG205MX-proBNI5-yEGFP-BNI5(41–448). The 1A/1D (S13), 3A/3D (S129, S270, and S278), and 6A/6DE (S327, S328, S349, T353, and S356) mutations were introduced to pRG205MX-proBNI5-yEGFP-BNI5(FL) via site-directed mutagenesis or in-fusion cloning. For the 1A/1D constructs, primer pairs P1869/P1870 and P1904/P1905 were used for site-directed mutagenesis. For 3A/3D, PCR fragments amplified from pRG205MX-proBNI5-yEGFP-BNI5(FL) using primer pairs P1974/P1975 or P1978/P1979 were subcloned via in-fusion into PCR-amplified vector backbones generated from the same template using primer pairs P1976/P1977 or P1980/P1981, respectively. For 6A/6DE, PCR fragments amplified using primer pairs P1982/P1983 or P1986/P1987 were subcloned via in-fusion into PCR-amplified vector backbones generated from the same template using primer pairs P1984/P1985 or P1988/P1989, respectively.

To construct YIp128-proACT1-GFP-ECM25-(536–588 AA)-tADH1, a ∼0.2-kb fragment encoding *ecm25(536–588)* was amplified by PCR from pUG36-GFP-Ecm25-xACT(536–588 aa) using primers P254 and P549. A ∼5.8-kb vector backbone containing the *ACT1* promoter was generated by inverse PCR from YIp128-proACT1-PKC1C1-GFP-tADH1 (lab stock) using primers P255 and P550. The two fragments were assembled using the in-fusion cloning kit.

The pUG36-BNI5* plasmid series, expressing GFP-tagged *BNI5* genes (e.g., pUG36-BNI5[FL]), were constructed by gap repair cloning (Oldenburg et al., 1997) or restriction enzyme–based cloning as indicated in the footnotes of Table S2.

### Live-cell imaging and data analysis

Quantitative time-lapse imaging analysis was performed as described previously with slight modifications (Okada et al., 2021a). For time-lapse microscopy, cells were cultured to exponential phase at 25°C in SC medium, briefly sonicated at 15% power for 5 s to declump (model Q55, Qsonica), concentrated by centrifugation, and spotted onto a concanavalin-A–coated glass-bottom chamber dish. Once cells were attached to the bottom, excess cells were removed by discarding the supernatant, and 1 ml fresh SC medium was added to the dish. For samples imaged at 37°C, cells were pre-cultured at 37°C for 1 h prior to harvesting for time-lapse analysis. Imaging was performed at room temperature (23°C) or 37°C, controlled by the OKO temperature control system (Okolab). Images were acquired by spinning-disk confocal microscopy using a Nikon microscope (model Eclipse Ti2-U; Nikon) equipped with a 100×/1.49 NA oil objective (model CFI Apo TIRF 100×; Nikon) and a confocal scanner unit (model CSU-X1; Yokogawa). An EMCCD camera (model Evolve 512 Delta; Photometrics) was used for capture. Solid-state lasers for excitation (488 nm for GFP and 561 nm for RFP) were housed in a launch (model ILE-400; Spectral Applied Research). The imaging system was controlled by MetaMorph version 7.10.4.431 (Molecular Devices). All time-lapse images, except for actin cables (GFP-xACT), were taken every 1, 1.5, or 2 min with 11 z-stacks with a step-size of 0.8 μm. Images of GFP-xACT were taken every 1 s at a single fixed focal plane. A sum or max projection was created with NIH ImageJ (1.53 t). The quantification of fluorescence intensities was performed as described previously (Okada et al., 2021a). In brief, the fluorescent intensity at the division site was calculated by subtracting the background intensity from that of the division site using sum projection images. For selected proteins, the data were normalized to the peak intensity (100%) of the GFP signal. To calculate the constriction rate, we manually measured the myosin ring diameter during constriction from max projection images acquired through time-lapse imaging. Then, we calculated the slope of the diameter curve from three to four time points, including midpoint of constriction. To determine the rate of actin cable flow, we manually marked an endpoint of an extending actin cable and measured the distance between endpoints in each consecutive time frame. Data analyses were performed with Microsoft Excel, GraphPad Prism 9.4.1, and R (ver. 3.0.1).

### FRAP

FRAP analysis was performed as described previously (Okada et al., 2019). Imaging samples were prepared as described above (see Live-cell imaging and data analysis). The imaging system used consists of a spinning-disk confocal scanner unit (model CSU-X1, Yokogawa) and an Olympus microscope (model IX81; Olympus) equipped with a 100×/1.40 NA oil objective (model UPlanSApo 100×/1.40 Oil; Olympus) and an EMCCD camera (model iXon X3 897, Andor Technology). MetaMorph ver. 7.8.10.0 (Molecular Devices) was used for hardware control and image acquisition. Diode lasers (488 nm for GFP and 561 nm for RFP) controlled via laser merge module (model LMM5, Spectral Applied Research) were used for excitation. Images were taken at room temperature with 11 z-stacks with a step size of 0.7 µm. To induce photobleaching, a diode-pumped 405 nm laser (model DL405-050-O CrystaLaser) was applied to a defined subcellular region. Maximum projections were created and analyzed with NIH ImageJ. In ImageJ, a polygon was drawn encircling the bleached area to calculate the integrated density within the area over time. Data of recovery curve were analyzed with GraphPad Prism 9. The estimated maximum amount of recovery (max) and half-time of recovery (t_1/2_) were determined using the one phase-association function in the GraphPad.

### Yeast growth assay

A spot assay was performed to examine cell growth under different conditions. Cells were cultured in SC or YPD medium at 25°C for 18 h and then diluted in fresh SC or YPD medium to an OD_600_ of 0.1. The cell suspension was subjected to 10-fold serial dilutions and inoculated as 5-μl spots onto SC or YPD plates. For overexpression of *BNI5* alleles, the cell suspension was spotted onto methionine-depleted SC plates. After incubation at 25°C or 37°C for 2 or 3 days, growth was recorded.

### Purification and in vitro binding of recombinant proteins

For testing Bni5 interactions with Elm1 and Myo1, Rosetta DE3 cells transformed with pGEX-4T-1 (GST alone), pGEX-4T-1-Bni5(1–448) (GST-Bni5-FL), pGEX-4T-1-Bni5(1–40) (GST-HR1), pGEX-4T-1-Bni5(306–393) (GST-Ext-HR2), pGEX-4T-1-Bni5(340–448) (GST-HR2-HR3), pGEX-4T-1-Bni5(306–448) (GST-Ext-HR2-HR3), pET His6 Sumo TEV LIC (6xHis-SUMO alone), pET-His6-Sumo-Elm1_1-420_, pET-His6-Sumo-Elm1_421-640_, pMAL-c2 (MBP only), or pMAL-MYO1-mTD1 were grown to an OD_600_ 0.6-1.0 before being induced for 3 h with 0.3 mM IPTG (Lab Scientific) at 25°C. Cells were then lysed by sonication (six times for 15 s each at 15% power, model Q55; Qsonica) in one of the following buffers, each containing the Protease Inhibitors Cocktail tablets (Roche) and 1 mg/ml lysozyme (L6876; Sigma-Aldrich): the GST lysis buffer (50 mM Tris-HCl, pH 7.5, 300 mM NaCl, 1.25 mM EGTA, 1 mM DTT, and 0.1 % NP-40), the 6xHis-SUMO lysis buffer (50 mM Tris-HCl, pH 7.5, 300 mM NaCl, 1.25 mM EGTA, 1 mM DTT, 0.1% NP-40, and 15 mM imidazole), or the MBP lysis buffer (20 mM Tris-HCl, pH 7.5, 200 mM NaCl, 1.25 mM EGTA, 1 mM DTT, and 0.1% NP-40). The resultant lysates were then centrifuged at 24,000 × *g* for 30 min at 4°C. The supernatants were then incubated with either Glutathione Sepharose 4B (GE Healthcare), Complete His-Tag Purification Resin (Roche), or Amylose Resin (New England Biolabs), which had been prewashed with respective lysis buffer, for 1 h at 4°C. The beads were then washed five times with the respective lysis buffer. GST-tagged proteins were kept with Glutathione Sepharose 4B and resuspended in Bead storage buffer (50 mM Tris-HCl, pH 7.5, 5 mM MgCl_2_, and 25% glycerol), while 6xHis-SUMO–tagged proteins and MBP-tagged proteins were eluted by the His elution buffer (50 mM Tris-HCl, pH 7.5, 300 mM NaCl, 1.25 mM EGTA, 1 mM DTT, 0.1% NP-40, and 300 mM imidazole) or the MBP elution buffer (20 mM Tris-HCl, pH 7.5, 200 mM NaCl, 1.25 mM EGTA, 1 mM DTT, 0.1% NP-40, and 10 mM Maltose), respectively. Protein concentrations were determined by standard curve intensity measurements from Coomassie blue–stained bovine serum albumin (A7617; Sigma-Aldrich) of known concentrations.

For the in vitro–binding assay, 3.5 μg of GST, GST-Bni5-FL, GST-Bni5-HR1, GST-Bni5-Ext-HR2, GST-Bni5-HR2-HR3, or GST-Bni5-Ext-HR2-HR3 was incubated with 3.5 μg of either His6-Sumo, His6-Sumo-Elm1-C, His6-Sumo-Elm1-N, MBP, or MBP-Myo1-mTD1 for 1 h with rotation in binding buffer (20 mM MOPS, pH 7.0, 1 mM EGTA, 150 mM NaCl, 1 mM DTT, and 0.1% NP-40). After centrifugation, the beads were then washed five times with fresh binding buffer before being extracted with 30 μl of 2× Laemmli Buffer (Bio-Rad Laboratories). 10 μl of the samples were separated via SDS-PAGE and stained with SimplyBlue SafeStain, while 1 μl of the samples were separated via SDS-PAGE and then transferred to a PVDF membrane before immunoblotting with the anti-6xHis (1:5,000 dilution, ab18184; abcam) antibody or MBP (1:10,000, M1321; Sigma-Aldrich) primary antibody, respectively, and HRP-labeled secondary antibody and ECL reagents from the Pierce Fast Western kit (Thermo Fisher Scientific).

For testing Bni5 interaction with septin filaments, plasmids pMVB128 (Cdc10, His6-Cdc12) and pMVB133 (Cdc3, Cdc11) with or without pCOLA-Duet-[His less]-Shs1 were co-transformed into *Escherichia coli* strain BL21(DE3) to generate 4-septin or 5-septin complexes, respectively. The transformed cells were grown to an OD_600_ 0.6–1.0 before being induced for 4 h with 1.0 mM IPTG (Lab Scientific) at 37°C, harvested by centrifugation, washed once with PBS, and stored at −80°C. Frozen cells were lysed in Renz buffer (300 mM NaCl, 2 mM MgCl_2_, 15 mM imidazole, 12% [vol/vol] glycerol, 50 mM Tris-HCl, pH 7.5, Protease Inhibitors Cocktail tablets, and 1 mg/ml lysozyme) by sonication (10 times for 20 s each at 25% power, model Q55; Qsonica). The resultant lysates were then centrifuged at 24,000 × *g* for 30 min at 4°C. The supernatants were then incubated with the Complete His-Tag Purification Resin (Roche) that had been prewashed with Renz buffer for 1 h at 4°C. The beads were then washed five times with Renz buffer. Protein complexes were then eluted with the elution buffer (300 mM NaCl, 2 mM MgCl_2_, 315 mM imidazole, 12% [vol/vol] glycerol, and 50 mM Tris-HCl, pH 7.5). It is worth noting that GST-tagged proteins underwent the same protein purification process as described above and were eluted with the GST elution buffer (50 mM Tris-HCl, pH 7.5, 300 mM NaCl, 1.25 mM EGTA, 1 mM DTT, 0.1% NP-40, and 10 mM glutathione) for binding assay with septin filaments. Protein concentrations were determined by standard curve intensity measurements from Coomassie blue–stained bovine serum albumin of known concentrations.

Septin filament formation was performed using an established protocol (Renz et al., 2013) with some modifications: purified septin complexes in the high-salt buffer (300 mM NaCl) were diluted to the low-salt buffer (50 mM NaCl) by adding the dilution buffer (2 mM MgCl_2_, 15 mM imidazole, 12% [vol/vol] glycerol, and 50 mM Tris-HCl, pH 7.5) and then kept at 4°C for 16 h to allow filament assembly. Septin complexes and filaments were then concentrated using Centrifugal Filter (Amicon Ultra-15; Sigma-Aldrich, 10 kDa molecular weight cutoff).

For the in vitro–binding assay, 1.5 μg of GST, GST-Bni5-FL, GST-Bni5-HR1, GST-Bni5-Ext-HR2, GST-Bni5-HR2-HR3, or GST-Bni5-Ext-HR2-HR3 was incubated with 1.5 μg of the concentrated 4-septin (Cdc11-Cdc12[His6]-Cdc3-Cdc10-Cdc10-Cdc3-Cdc12[His6]-Cdc11) or 5-septin (a mixture of Cdc11-Cdc12[His6]-Cdc3-Cdc10-Cdc10-Cdc3-Cdc12[His6]-Cdc11 and Shs1-Cdc12[His6]-Cdc3-Cdc10-Cdc10-Cdc3-Cdc12[His6]-Shs1) complexes/filaments in the binding buffer (50 mM NaCl, 2 mM MgCl_2_, 15 mM imidazole, 12% [vol/vol] glycerol, and 50 mM Tris-HCl, pH 7.5) with rotation for 1 h at room temperature. The reactions were subjected to centrifugation at 100,000 × *g* for 1 h at 4°C to pellet the septin filaments and the associated proteins. The pellets were washed once with fresh binding buffer before being extracted with 35 μl of 2× Laemmli Buffer. 10 μl of the samples were separated via SDS-PAGE and stained with SimplyBlue SafeStain, while 1 μl or 0.2 μl of the samples were separated via SDS-PAGE and then transferred to a PVDF membrane before immunoblotting with the anti-GST (1:3,000 dilution, ab92; abcam) or anti-SHS1 (1:5,000, a gift from Doug Kellogg, University of California, Santa Cruz, CA, USA) primary antibody and visualized as described above.

### Mass spectrometry sample preparation, instrumentation, and analysis

Sample preparation for mass spectrometry analysis of proteins isolated from yeast cultures labeled with stable isotopes was performed as previously described, with modifications (Baro et al., 2018). Yeast strains YEF9590 (*arg4Δ*), YEF9695 (*arg4Δ elm1Δ*), and YEF10671 (*arg4Δ gin4Δ*) were cultured in 100 ml SC medium containing ^13^C_6_-lysine and ^13^C_6_-arginine (heavy) or unlabeled lysine and arginine (light) until the exponential phase at 25°C (∼10 generations). Protein extracts were obtained by cell lysis using bead beating in 600 μl MS buffer (4 M urea, 50% trifluoroethanol, and 50 nM ammonium bicarbonate). Cell lysates were mixed in a 1:1 ratio based on protein concentration as determined by the Bradford assay.

Protein samples were reduced with dithiothreitol, alkylated with iodoacetamide, and digested with trypsin. Digested peptides were cleaned using Sep-Pak C18 columns (Waters). Approximately four-fifths of the sample peptides were subjected to two sequential TiO_2_ purifications (GL Sciences) to enrich phosphorylated peptides. For each sample, the enriched peptides were pooled and analyzed in a single extended 2.5-h LC-MS/MS run on a Q Exactive HF mass spectrometer (Thermo Fisher Scientific). The remaining one-fifth of the peptides (not phospho-enriched) was subjected to global proteome analysis using a single separate extended 2.5-h LC-MS/MS run on the same instrument. Both datasets were analyzed independently.

The protein samples for mass spectrometry analysis of recombinant-purified proteins were prepared as described previously, with slight modification (Marquardt et al., 2024). GST-Elm1 and 6xHis-SUMO-Bni5, or GST-Bni5 and 6xHis-SUMO-Gin4, were purified as described above (see Protein purification and in vitro–binding assays). 1 μg of 6xHis-SUMO-Bni5 or GST-Bni5 was incubated with either buffer (no kinase sample) or 1 μg GST-Elm1 or 6xHis-SUMO-Gin4, in the presence of 2 mM ATP, for 30 min at 30°C with shaking. Samples were separated by SDS-PAGE and stained with SimplyBlue SafeStain. The gel bands corresponding to the predicted size of 6xHis-SUMO-Bni5 were excised, reduced with Tris (2-carboxyethyl) phosphine hydrochloride, alkylated with iodoacetamide, and digested with trypsin. Tryptic digests were analyzed using a standard 90-min LC gradient on the Thermo Q Exactive Plus mass spectrometer (Thermo Fisher Scientific).

Mass spectrometry data were searched with full tryptic specificity against the Swiss-Prot *S. cerevisiae* database (07/26/2021) and a common contaminant database using MaxQuant 1.6.3.3. Variable modifications included: acetylation (+42.01056) at the protein N terminus, oxidation (+15.99491) on methionine, deamidation (+0.98402) on asparagine, and phosphorylation (+79.9663) on serine, threonine, and tyrosine. Proteins and peptides were filtered to remove common contaminants and incorrectly assigned modifications. Protein quantification was performed using Razor + unique peptides. Razor peptides are shared (nonunique) peptides assigned to the protein group with the most other peptides (Occam’s razor principle). Protein and peptide abundances were quantified based on intensity (sum of the peptide MS peak areas). Phosphorylated residues, the primary modifications of interest, were identified, filtered, and compared between the no-kinase control and kinase-treated samples (Elm1 or Gin4).

### PREM imaging, sample preparation, and analysis

PREM was performed as described previously (Ong et al., 2014). Briefly, strains in the *bar1Δ* background were cultured in 50 ml YM-1 medium at 25°C with shaking until reaching mid-log phase. To arrest cells in G1 phase, α-factor pheromone (Zymo Research) was added at 50 ng/ml, and cultures were incubated for 3 h at 25°C. Cells were then washed and released into α-factor–free YM-1 medium and incubated for an additional 1.5 h at 25°C to enrich for small- and medium-budded cells, in which septins are organized into an hourglass structure.

Following harvest, cells were subjected to spheroplasting using spheroplasting buffer (10 mM PIPES and 1.2 M sorbitol, pH 6.5) containing 0.3 mg/ml zymolyase-100T (AMS Biotechnology). Spheroplasts were mounted onto poly-L-lysine–coated coverslips. To unroof the cells, two coverslips with spheroplasts facing each other were gently pressed together and immediately fixed in 2% glutaraldehyde in 1× KHMgE buffer (70 mM KCl, 20 mM HEPES, 5 mM MgCl_2_, and 3 mM EGTA) for 20 min at room temperature.

Coverslips containing fixed, unroofed spheroplasts were quenched sequentially in 2 mg/ml and 5 mg/ml NaBH_4_ in PBS for 10 min each, followed by blocking in 1% glycine for 10 min. After washing three times in PBS, samples were blocked in PBS containing 5% donkey serum for 30 min. Coverslips were incubated with a goat polyclonal anti-GFP antibody (1:50, ab5450; Abcam in PBS with 5% donkey serum) at room temperature for 1.5 h. After five washes with PBS and a 10-min block in immunogold-labeling buffer (20 mM Tris-HCl, pH 8.0, 0.5 M NaCl, and 0.05% Tween 20) containing 0.5% donkey serum, the coverslips were incubated overnight at room temperature with 18-nm colloidal gold-conjugated donkey anti-goat IgG (H+L) (705-215-147; Jackson ImmunoResearch, 1:5 dilution) in immunogold-labeling buffer supplemented with 5% donkey serum. After five washes in immunogold-labeling buffer containing 0.05% donkey serum, the samples were post-fixed in 2% glutaraldehyde in 0.1 M sodium cacodylate buffer (pH 7.3).

Coverslips were then processed for PREM as previously described (Ong et al., 2014; Svitkina, 2009). Samples were treated with 0.1% tannic acid in water for 20 min, rinsed three times with water, and stained with 0.2% uranyl acetate in water for 20 min. Dehydration was performed through a graded ethanol series (10%, 20%, 40%, 60%, 80%, and 100%), followed by critical point drying using a Samdri PVT-3D (Tousimis Research Corporation) critical point dryer. Samples were rotary shadowed with platinum at a 45° angle to form a ∼2-nm layer, followed by carbon coating at a 90° angle to deposit a 3.5–4 nm layer.

After detaching the glass coverslips with hydrofluoric acid, the coated replicas were mounted on EM grids and imaged using a JEM 1011 transmission electron microscope (JEOL USA) operated at 100 kV. Images were acquired with an ORIUS 832.10W charge-coupled device camera (Gatan) and presented in inverted contrast. Structures of interest were color-labeled using Adobe Photoshop. To assess septin morphology, structures on the cortical surface that were primarily labeled with immunogold particles to detect Cdc3-GFP were selected. These structures were categorized into two morphological classes: septin sheets, defined as tightly packed arrays of filaments forming a sheet-like structure; and septin bundles, defined as groups of filaments that are connected but spaced apart, forming a network-structure.

### Quantification and statistical analysis

For the statistical analyses of intensities at subcellular region (related to Fig. 3 G, Fig. 8 H, and Fig. S3, C–E), diameter of septin hourglass (Fig. S3 F), and rates of signal drop (Fig. 4 G), retrograde flow of actin cables (Fig. 7 C), and Myo1 constriction (Fig. S5 C), a two-sided Mann–Whitney U test was performed. “*n*” refers to the number of cells analyzed unless indicated otherwise.

### Online supplemental material

Fig. S1 shows cell growth and morphological suppression of the *cdc12-6* septin mutant by overexpression of Bni5-N-GFP, but not Bni5-C-GFP. Fig. S2 shows identification of septin- and Elm1-dependent localization domains in Bni5; western blot verification of Shs1 presence in the five-septin complex; and western blot confirmation of Bni5 fragment interactions with four- and five-septin complexes. Fig. S3 shows impact of *BNI5* deletion on the architecture, size, and composition of the septin hourglass. Fig. S4 shows in vitro kinase assays and identification of Elm1- and Gin4-dependent phosphorylation sites in Bni5; functional analyses of the in vivo and in vitro phosphorylation sites in Bni5; dissociation kinetics of Bni5 in relation to Gin4 at the bud neck prior to cytokinesis; and differential effects of mitotic exit on the bud neck localization of Gin4, Elm1, and Bni5. Fig. S5 shows effect of *BNI5* deletion on cell growth, morphology, and Myo1 constriction at 25°C and 37°C. Table S1 shows yeast strains used in this study. Table S2 shows plasmids used in this study. Table S3 shows oligonucleotides used in this study.

## Supplementary Material

Table S1 shows strains used in this study.

Table S2 shows plasmids used in this study.

Table S3 shows oligonucleotides used in this study.

SourceData F3 is the source file for Fig. 3.

SourceData F5 is the source file for Fig. 5.

SourceData F6 is the source file for Fig. 6.

SourceData FS2 is the source file for Fig. S2.

## Data Availability

The data supporting the findings of this study are included in the paper and its supplemental information and will be available from the primary corresponding author (Erfei Bi via email: ebi@pennmedicine.upenn.edu) upon request.
