# Supplementary material for: Bni5 regulates and coordinates septin architecture and myosin-II functions at the cell division site
Source: J Cell Biol. 2025 Nov 6;224(12):e202311040. doi: 10.1083/jcb.202311040 (PMC12591035; doi:10.1083/jcb.202311040)
Supplement: Table S1 — shows strains used in this study. [file jcb_202311040_tables1.docx]

| **Strain** | **Genotype** | **Source** |
| --- | --- | --- |
| YEF473 | *MAT*a/α *trp1-Δ63/trp1-Δ63 leu2-Δ1/leu2-Δ1 ura3-52/ura3-52 his3-Δ200/his3-Δ200 lys2-801/lys2-801* | (Bi and Pringle, 1996) |
| YEF473A | *MAT*a *trp1-Δ63 leu2-Δ1 ura3-52 his3-Δ200 lys2-801* | (Bi and Pringle, 1996) |
| YEF473B | *MAT*α *trp1-Δ63 leu2-Δ1 ura3-52 his3-Δ200 lys2-801* | (Bi and Pringle, 1996) |
| YEF743 (M-17) | *MAT*a *cdc12-6 leu2 ura3* | (Caviston et al., 2003) |
| Masa1243 | As YEF473A except *myo1Δ::URA3-KanMX6* [CEN *HIS3 MYO1-GFP*] | (Fang et al., 2010) |
| XDY 258 | As YEF473A except *myo1-mTD1-GFP bni5Δ::His3MX6* | (Fang et al., 2010) |
| YEF2057 | As YEF473B except *myo1Δ::HIS3* [CEN *URA3 MYO1*] | Lab stock |
| YEF4857 | As YEF473A except *CDC11-mCherry-His3MX6* | Lab stock |
| YEF5797 | As YEF473A except *cdc10Δ::KanMX6 CDC3::CDC3-mCherry-LEU2* | (Wloka et al., 2011) |
| YEF5799 | As YEF473A except *shs1Δ::KanMX6 CDC3::CDC3-mCherry-LEU2* | Lab stock |
| YEF5804 | As YEF473A except *CDC3::CDC3-mCherry-LEU2* | (Fang et al., 2010) |
| YEF6108 | As YEF473A except *MYO1-GFP-KanMX CDC3::CDC3-mCherry-LEU2* | Lab stock |
| YEF6349 | As YEF473A except *myo1Δ::His3MX6 CDC3::CDC3-mCherry-LEU2* | (Wloka et al., 2013) |
| YEF6608 | As YEF473 except *GFP-myo1(1-1797)-KanMX/GFP-myo1(1-1797)-KanMX* | Lab stock |
| YEF6611 | As YEF473 except *GFP-MYO1-KanMX/GFP-MYO1-KanMX* | Lab stock |
| YEF7066 | As YEF473A except *cdc11Δ::TRP1 CDC3::CDC3-mCherry-LEU2* | Lab stock |
| YEF8356 | As YEF473A except *cdc15-2-LEU2* | (Chen et al., 2020) |
| YEF8390 | As YEF473A except *TUB1::HPH-proHIS3-mRuby2-TUB1* | (Okada et al., 2021b) |
| YEF8391 | As YEF473A except *TUB1::HPH-proHIS3-mRuby2-TUB1 MLC2-mApple-URA3MX* | (Okada et al., 2021b) |
| YEF8437 | As YEF473A except *ELM1-GFP-His3MX6 TUB1::HPH-proHIS3-mRuby2-TUB1* | This study^a^ |
| YEF8937 | As YEF473A except [CEN *URA3 MYO1*] | This study^b^ |
| YEF8950 | As YEF473A except *myo1Δ::NatMX6* [CEN *URA3 MYO1*] | This study^c^ |
| YEF9180 | As YEF473A except *CDC3::CDC3-GFP-LEU2 TUB1::HPH-proHIS3-mRuby2-TUB1* | (Marquardt et al., 2024) |
| YEF9305 | As YEF473A except *CDC3::CDC3-mCherry-LEU2 ELM1-GFP-URA3MX* | (Marquardt et al., 2020) |
| YEF9313 | As YEF473A except *CDC3::CDC3-mCherry-LEU2 ELM1-GFP-URA3MX bni5Δ::His3MX6* | This study^d^ |
| YEF9333 | As YEF473A except *bar1Δ::HIS3MX6 CDC3::CDC3-GFP-LEU2* | (Marquardt et al., 2020) |
| YEF9336 | As YEF473A except *CDC3::CDC3-mCherry-LEU2 BNI5-GFP-His3MX6* | This study^e^ |
| YEF9369 | As YEF473A except *elm1Δ::KanMX6 CDC3::CDC3-mCherry-LEU2 BNI5-GFP-His3MX6* | This study^f^ |
| YEF9380 | As YEF473A except *cdc15-2-LEU2 bni5Δ::His3MX6* | This study^d^ |
| YEF9473 | As YEF473A except *GFP-MYO1* | This study^g^ |
| YEF9590 | As YEF473A except *arg4Δ::TRP1* | This study^h^ |
| YEF9618 | As YEF473A except *CDC3::CDC3-GFP-LEU2 TUB1::HPH-proHIS3-mRuby2-TUB1 bni5Δ::His3MX6* | This study^d^ |
| YEF9654 | As YEF473A except *bni5Δ::NatMX6* | This study^i^ |
| YEF9695 | As YEF473A except *arg4Δ::TRP1 elm1Δ::KanMX6* | This study^f^ |
| YEF9767 | As YEF473A except *bni5Δ::His3MX6 bar1Δ::HIS3MX6 CDC3::CDC3-GFP-LEU2* | (Marquardt et al., 2020) |
| YEF10170 | As YEF473A except *myo1Δ::NatMX6 leu2::proACT1-GFP-ECM25-(536-588)-LEU2* [CEN *URA3 MYO1*] | This study^j^ |
| YEF10173 | As YEF473A except *bni5Δ::NatMX6 leu2::proACT1-GFP-ECM25-(536-588)-LEU2* | This study^j^ |
| YEF10201 | As YEF473A except *leu2::proACT1-GFP-ECM25-(536-588)-LEU2* | This study^j^ |
| YEF10276 | As YEF473A except *GFP-BNI5* | (Marquardt et al., 2020) |
| YEF10293 | As YEF473A except *GFP-BNI5 CDC3::CDC3-mCherry-LEU2* | This study^k^ |
| YEF10296 | As YEF473A except *CDC11-GFP-His3MX6* | This study^l^ |
| YEF10315 | As YEF473A except *elm1Δ::KanMX6 GFP-BNI5 CDC3::CDC3-mCherry-LEU2* | This study^f^ |
| YEF10440 | As YEF473A except *ELM1-GFP-His3MX6 CDC3::CDC3-mCherry-LEU2* | (Marquardt et al., 2024) |
| YEF10667 | As YEF473A except *TUB1::HPH-proHIS3-mScarlet-I-TUB1* | This study^m^ |
| YEF10671 | As YEF473A except *arg4Δ::TRP1 gin4Δ::URA3-KanMX6* | This study^n^ |
| YEF10885 | As YEF473A except *CDC10-mScarlet-I-URA3MX* | This study^o^ |
| YEF10958 | As YEF473A *MYO1-mScarlet-I-KanMX* | This study^p^ |
| YEF10993 | As YEF473A except *bni5Δ::NatMX6 MYO1-mScarlet-I-KanMX* | This study^q^ |
| YEF10994 | As YEF473A except *bni5Δ::NatMX6 CDC3::CDC3-mCherry-LEU2* | This study^k^ |
| YEF11027 | As YEF473A except *bni5Δ::NatMX6 MYO1-mScarlet-I-KanMX* [CEN *URA3 GFP-bni5(41-448)*] | This study^r^ |
| YEF11029 | As YEF473A except *bni5Δ::NatMX6 MYO1-mScarlet-I-KanMX* [CEN *URA3 GFP-BNI5(FL)*] | This study^r^ |
| YEF11033 | As YEF473A except *bni5Δ::NatMX6 MYO1-mScarlet-I-KanMX* [CEN *URA3 GFP*] | This study^r^ |
| YEF11039 | As YEF473A except *bni5Δ::NatMX6 CDC3::CDC3-mCherry-LEU2* [CEN *URA3 GFP-bni5(306-448)*] | This study^r^ |
| YEF11040 | As YEF473A except *bni5Δ::NatMX6 CDC3::CDC3-mCherry-LEU2* [CEN *URA3 GFP-bni5(340-448)*] | This study^r^ |
| YEF11053 | As YEF473A *except bni5Δ::NatMX6 CDC3::CDC3-mCherry-LEU2* [CEN *URA3 GFP-BNI5(FL)*] | This study^r^ |
| YEF11071 | As YEF473A except *CDC3::CDC3-mCherry-LEU2* [CEN *URA3 GFP-bni5(1-40)*] | This study^r^ |
| YEF11087 | As YEF473A except *SHS1-GFP-HIS3 TUB1::HPH-proHIS3-mRuby2-TUB1* | This study^s^ |
| YEF11088 | As YEF473A except *CDC11-GFP-His3MX6 MYO1-mScarlet-I-KanMX* | This study^q^ |
| YEF11114 | As YEF473A except *myo1Δ::His3MX6 CDC3::CDC3-mCherry-LEU2* [CEN *URA3 GFP-bni5(1-40)*] | This study^t^ |
| YEF11181 | As YEF473A except *bni5Δ::NatMX6 CDC3::CDC3-mCherry-LEU2* [CEN *URA3 GFP-bni5(306-339)*] | This study^r^ |
| YEF11190 | As YEF473A except *bni5Δ::NatMX6 elm1Δ::KanMX6 CDC3::CDC3-mCherry-LEU2* [CEN *URA3 GFP-BNI5(FL)*] | This study^r^ |
| YEF11193 | As YEF473A except *bni5Δ::NatMX6 elm1Δ::KanMX6 CDC3::CDC3-mCherry-LEU2* [CEN *URA3 GFP-bni5(306-448)*] | This study^r^ |
| YEF11194 | As YEF473A except *bni5Δ::NatMX6 elm1Δ::KanMX6 CDC3::CDC3-mCherry-LEU2* [CEN *URA3 GFP-bni5(340-448)*] | This study^r^ |
| YEF11197 | As YEF473A except *bni5Δ::NatMX6 elm1Δ::KanMX6 CDC3::CDC3-mCherry-LEU2* [CEN *URA3 GFP-bni5(306-393)*] | This study^r^ |
| YEF11207 | As YEF473A except *CDC3::CDC3-mCherry-LEU2* [CEN *URA3 GFP-bni5(306-393)*] | This study^r^ |
| YEF11211 | As YEF473A except *CDC11-GFP-His3MX6 MYO1-mScarlet-I-KanMX bni5Δ::GBP-URA3MX* | This study^u^ |
| YEF11212 | As YEF473A except *CDC11-GFP-His3MX6 MYO1-mScarlet-I-KanMX bni5(1-40)-GBP-URA3MX* | This study^u^ |
| YEF11247 | *cdc12-6 bni5Δ::NatMX6* | This study^v^ |
| YEF11254 | As YEF473A except *bni5Δ::GBP-URA3MX* | This study^w^ |
| YEF11255 | As YEF473A except *bni5(1-40)-GBP-URA3MX* | This study^w^ |
| YEF11277 | As YEF473A except *bni5Δ::NatMX6 elm1Δ::KanMX6 CDC3::CDC3-mCherry-LEU2* | This study^x^ |
| YEF11312 | *cdc12-6 bni5Δ::NatMX6* [CEN URA3 GFP-BNI5(FL)] | This study^r^ |
| YEF11313 | *cdc12-6 bni5Δ::NatMX6* [CEN URA3 GFP] | This study^r^ |
| YEF11362 | *cdc12-6 bni5Δ::NatMX6* [CEN URA3 BNI5] | This study^r^ |
| YEF11363 | *cdc12-6 bni5Δ::NatMX6* [CEN URA3 BNI5-C-GFP] | This study^r^ |
| YEF11379 | As YEF473A except *myo1-mTD1Δ* | This study^y^ |
| YEF11385 | As YEF473A except *TUB1::HPH-proHIS3-mScarlet-I-TUB1 MYO1-GFPEnvy-URA3MX* | This study^z^ |
| YEF11408 | As YEF473A except *myo1-mTD1Δ CDC3::CDC3-mCherry-LEU2* | This study^k^ |
| YEF11417 | As YEF473A except *MYO1-GFPEnvy-URA3MX* | This study^z^ |
| YEF11418 | As YEF473A except *myo1-mTD1Δ-GFPEnvy-URA3MX* | This study^z^ |
| YEF11441 | As YEF473A except *MYO1-GFPEnvy-URA3MX TUB1::HPH-proHIS3-mScarlet-I-TUB1* | This study^m^ |
| YEF11442 | As YEF473A except *myo1-mTD1Δ-GFPEnvy-URA3MX TUB1::HPH-proHIS3-mScarlet-I-TUB1* | This study^m^ |
| YEF11443 | As YEF473A except *bni5Δ::NatMX6 TUB1::HPH-proHIS3-mScarlet-I-TUB1 MYO1-GFPEnvy-URA3MX* | This study^v^ |
| YEF11489 | As YEF473A except *myo1-mTD1Δ leu2::proACT1-GFP-ECM25-(536-588)-LEU2* | This study^j^ |
| YEF11501 | As YEF473A except *GFP-myo1(1-1797)-KanMX* | This study^aa^ |
| YEF11522 | As YEF473A except *bni5Δ::NatMX6 TUB1::HPH-proHIS3-mScarlet-I-TUB1* | This study^m^ |
| YEF11524 | As YEF473A except *GFP-myo1(1-1797)-KanMX TUB1::HPH-proHIS3-mScarlet-I-TUB1* | This study^m^ |
| YEF11529 | As YEF473A except *GFP-MYO1 TUB1::HPH-proHIS3-mScarlet-I-TUB1* | This study^m^ |
| YEF11530 | As YEF473A except *bni5Δ::NatMX6 GFP-myo1(1-1797)-KanMX TUB1::HPH-proHIS3-mScarlet-I-TUB1* | This study^v^ |
| YEF11546 | As YEF473A except *bni5Δ::NatMX6 TUB1::HPH-proHIS3-mScarlet-I-TUB1 leu2::proBNI5-GFP-BNI5(FL)-LEU2MX* | This study^ab^ |
| YEF11553 | As YEF473A except *bni5Δ::NatMX6 TUB1::HPH-proHIS3-mScarlet-I-TUB1 leu2::proBNI5-GFP-bni5(Δ41-305)-LEU2MX* | This study^ab^ |
| YEF11600 | As YEF473A except *bni5Δ::NatMX6 TUB1::HPH-proHIS3-mScarlet-I-TUB1 leu2::proBNI5-GFP-bni5(41-448)-LEU2MX* | This study^ab^ |
| YEF11601 | As YEF473A except *bni5Δ::NatMX6 GFP-MYO1 TUB1::HPH-proHIS3-mScarlet-I-TUB1* | This study^v^ |
| YEF11624 | As YEF473A except *TUB1::HPH-proHIS3-mScarlet-I-TUB1 BNI5-GFP-His3MX6* | This study^e^ |
| YEF11634 | As YEF473A except *CDC10-mScarlet-I-URA3MX bni5Δ::NatMX6* | This study^v^ |
| YEF11640 | As YEF473A except *CDC10-mScarlet-I-URA3MX bni5Δ::NatMX6 TUB1::HPH-proHIS3-Venus-TUB1* | This study^ac^ |
| YEF11750 | As YEF473A except *CDC3::CDC3-mCherry-LEU2 CDC10-GFP-His3MX6* | This study^ad^ |
| YEF11753 | As YEF473A except *TUB1::HPH-proHIS3-mScarlet-I-TUB1 CDC10-GFP-His3MX6* | This study^ad^ |
| YEF11754 | As YEF473A except T*UB1::HPH-proHIS3-ymScarlet-I-TUB1 CDC11-GFP-His3MX6* | This study^ae^ |
| YEF11755 | As YEF473A except T*UB1::HPH-proHIS3-ymScarlet-I-TUB1 CDC12-GFP-His3MX6* | This study^af^ |
| YEF11757 | As YEF473A except myo1-mTD1Δ CDC3::CDC3-mCherry-LEU2 [CEN URA3 GFP-bni5(1-40)] | This study^t^ |
| YEF11772 | As YEF473A except *CDC10-mScarlet-I-URA3MX bni5Δ::NatMX6 TUB1::HPH-proHIS3-Venus-TUB1 leu2::proBNI5-GFP-BNI5(FL)-LEU2MX* | This study^ag^ |
| YEF11773 | As YEF473A except *CDC10-mScarlet-I-URA3MX bni5Δ::NatMX6 TUB1::HPH-proHIS3-Venus-TUB1 leu2::proBNI5-GFP-bni5(Δ41-305)-LEU2MX* | This study^ag^ |
| YEF11811 | As YEF473A except *shs1Δ::KanMX6 CDC3::CDC3-mCherry-LEU2* [pUG36-BNI5(306-393)] | This study^ah^ |
| YEF11812 | As YEF473A except *cdc10Δ::KanMX6 CDC3::CDC3-mCherry-LEU2* [pUG36-BNI5(306-393)] | This study^ah^ |
| YEF11819 | As YEF473A except *cdc11Δ::KanMX6 CDC3::CDC3-mCherry-LEU2* [pUG36-BNI5(306-393)] | This study^ah^ |
| YEF11821 | As YEF473A except *bni5Δ::NatMX6 leu2::proBNI5-GFP-BNI5(FL)-LEU2MX* | This study^ai^ |
| YEF11823 | As YEF473A except *bni5Δ::NatMX6 leu2::proBNI5-GFP-bni5(306-393)-LEU2MX* | This study^ai^ |
| YEF11824 | As YEF473A except *bni5Δ::NatMX6 leu2::proBNI5-GFP-bni5(340-448)-LEU2MX* | This study^ai^ |
| YEF11825 | As YEF473A except *bni5Δ::NatMX6 leu2::proBNI5-GFP-bni5(306-448)-LEU2MX* | This study^ai^ |
| YEF12068 | As YEF473A except *cdc15-2-LEU2 TUB1::HPH-pHIS3-ymScarlet-TUB1* | This study^m^ |
| YEF12069 | As YEF473A except *bni5D::HIS3 cdc15-2-LEU2 TUB1::HPH-pHIS3-ymScarlet-TUB1* | This study^m^ |
| YEF12109 | As YEF473A except *bni5Δ::NatMX6 TUB1::HPH-pHIS3-ymScarlet-TUB1* *leu2::proBNI5-GFP-bni5(41-448)-S270A-T274A-S346A-S349A-S350A*-LEU2MX | This study^aj^ |
| YEF12112 | As YEF473A except *bni5Δ::NatMX6 TUB1::HPH-pHIS3-ymScarlet-TUB1* *leu2::proBNI5-GFP-bni5(41-448)-S270D-T274E-S346D-S349D-S350D-LEU2MX* | This study^aj^ |
| YEF12138 | As YEF473A except *cdc11Δ::KanMX6 CDC3::CDC3-mCherry-LEU2 bni5Δ::NatMX6* | This study^v^ |
| YEF12141 | As YEF473A except *TUB1::HPH-proHIS3-mRuby2-TUB1 SHS1-GFP-HIS3 bni5Δ::NatMX6* | This study^v^ |
| YEF12142 | As YEF473A except *TUB1::HPH-proHIS3-mScarlet-TUB1 CDC10-GFP-His3MX6 bni5Δ::NatMX6* | This study^v^ |
| YEF12143 | As YEF473A except *TUB1::HPH-proHIS3-ymScarlet-TUB1 CDC11-GFP-His3MX6* *bni5Δ::NatMX6* | This study^v^ |
| YEF12144 | As YEF473A except *TUB1::HPH-proHIS3-ymScarlet-TUB1 CDC12-GFP-His3MX6* *bni5Δ::NatMX6* | This study^v^ |
| YEF12194 | As YEF473A except *bni5Δ::NatMX6* [CEN *URA3 HOF1-GFP*] | This study^ak^ |
| YEF12227 | As YEF473A except *bni5Δ::His3MX cdc15-2-LEU2 TUB1::HPH-proHIS3-ymScarlet-TUB1 ura3::proBNI5-GFP-BNI5(FL)-URA3MX* | This study^al^ |
| YEF12237 | As YEF473A except *bni5Δ::NatMX* *hof1Δ*::*TRP1* [CEN *URA3 HOF1-GFP*] | This study^am^ |
| YEF12240 | As YEF473A except *bni5Δ::NatMX6 TUB1::HPH-pHIS3-ymScarlet-TUB1 leu2::proBNI5-GFP-bni5(41-448)-S198A-S225A-S289A-S327A-S354A-LEU2MX* | This study^aj^ |
| YEF12241 | As YEF473A except *bni5Δ::NatMX6 TUB1::HPH-pHIS3-ymScarlet-TUB1 leu2::proBNI5-GFP-bni5(41-448)-S198D-S225D-S289D-S327D-S354D-LEU2MX* | This study^aj^ |
| YEF12267 | As YEF473A except *bni5Δ::NatMX hof1Δ*::*TRP1 leu2::proBNI5-GFP-bni5(41-448)-LEU2MX* [CEN *URA3 HOF1-GFP*] | This study^an^ |
| YEF12280 | As YEF473A except *bni5Δ::NatMX hof1Δ::TRP1 leu2::proBNI5-GFP-BNI5(FL)-LEU2MX* [CEN *URA3 HOF1-GFP*] | This study^an^ |
| YEF12291 | As YEF473 except *myo1Δ*::*HIS3*/*GFP-myo1(1-1797)-KanMX* [CEN *URA3 MYO1*] | This study^ao^ |
| YEF12293 | As YEF473 except *myo1Δ*::*HIS3*/*GFP-myo1(1-1797)-KanMX* | This study^ao^ |
| YEF12297 | As YEF473 except *myo1Δ*::*HIS3*/*GFP-MYO1* | This study^ao^ |
| YEF12330 | As YEF473A except *bni5Δ::NatMX hof1Δ::TRP1 leu2::LEU2MX* [CEN *URA3 HOF1*] | This study^an^ |
| YEF12402 | As YEF473A except *cdc15-2-LEU2 TUB1::HPH-pHIS3-ymScarlet-TUB1 GIN4-GFP-HIS* | This study^ap^ |
| YEF12412 | As YEF473A except *cdc15-2-LEU2 TUB1::HPH-pHIS3-ymScarlet-TUB1 ELM1-GFP-HIS3* | This study^aq^ |
| YEF12427 | As YEF473A except *bni5Δ::NatMX6 leu2::proBNI5-GFP-BNI5(FL)-LEU2MX GIN4-mScarlet-KanMX6* | This study^ap^ |
| YEF12440 | As YEF473A except *bni5Δ::NatMX6 TUB1::HPH-pHIS3-ymScarlet-TUB1 leu2::proBNI5-GFP-bni5(FL)-S13A-LEU2MX* | This study^ar^ |
| YEF12441 | As YEF473A except *bni5Δ::NatMX6 TUB1::HPH-pHIS3-ymScarlet-TUB1 leu2::proBNI5-GFP-bni5(FL)-S13D-LEU2MX* | This study^ar^ |
| YEF12442 | As YEF473A except *bni5Δ::NatMX6 TUB1::HPH-pHIS3-ymScarlet-TUB1 leu2::proBNI5-GFP-bni5(FL)-S129A-S270A-S278A-LEU2MX* | This study^ar^ |
| YEF12443 | As YEF473A except *bni5Δ::NatMX6 TUB1::HPH-pHIS3-ymScarlet-TUB1 leu2::proBNI5-GFP-bni5(FL)-S129D-S270D-S278D-LEU2MX* | This study^ar^ |
| YEF12444 | As YEF473A except *bni5Δ::NatMX6 TUB1::HPH-pHIS3-ymScarlet-TUB1 leu2::proBNI5-GFP-bni5(FL)-S325A-S327A-S328A-S349A-T353A-S356A-LEU2MX* | This study^ar^ |
| YEF12445 | As YEF473A except *bni5Δ::NatMX6 TUB1::HPH-pHIS3-ymScarlet-TUB1 leu2::proBNI5-GFP-bni5(FL)-S325D-S327D-S328D-S349D-T353E-S356D-LEU2MX* | This study^ar^ |
| YEF12456 | As YEF473A except *CDC10-mScarlet-I-URA3MX bni5Δ::NatMX6 TUB1::HPH-proHIS3-Venus-TUB1 leu2::proBNI5-GFP-bni5(FL)-S325A-S327A-S328A-S349A-T353A-S356A-LEU2MX* | This study^ag^ |
| YEF12457 | As YEF473A except *CDC10-mScarlet-I-URA3MX bni5Δ::NatMX6 TUB1::HPH-proHIS3-Venus-TUB1 leu2::proBNI5-GFP-bni5(FL)-S325D-S327D-S328D-S349D-T353E-S356D-LEU2MX* | This study^ag^ |

**Table S1. Strains used in this study**

| ^a^ | A DNA fragment carrying *ELM1-GFP-His3MX6* was amplified by PCR using the chromosomal DNA from YEF8250 (Marquardt et al., 2020) with primers Elm1-Ftag-check and Elm1-R-check, and then transformed into YEF8390 to generate YEF8437. |
| --- | --- |
| ^b^ | A low-copy plasmid YCp50-MYO1 was transformed into YEF473A |
| ^c^ | A DNA fragment carrying *myo1Δ::NatMX* was amplified by PCR from pAG25 using primers MYO1-F1 and MYO1-R1 and then transformed into the YEF8937. |
| ^d^ | A DNA fragment carrying *bni5Δ::HIS3MX* was amplified by PCR using the chromosomal DNA from YEF6316 (lab stock) as the template and the pair of primers Bni5 244US ATG and Bni5 381bp DS TAG R and then transformed into the YEF9305, YEF8356, or YEF9180 to generate YEF9313, YEF9380, or YEF9618, respectively. |
| ^e^ | A DNA fragment carrying *BNI5-GFP-His3MX6* was amplified by PCR using the chromosomal DNA from YEF9290 (lab stock) as the template and the pair of primers P1168 and Bni5 381bp DS TAG R or of primers P1526 and P1167 and then transformed into the YEF5804 or YEF10667 to generate YEF9336 or YEF11624, respectively. |
| ^f^ | A DNA fragment carrying *elm1Δ::KanMX6* was amplified by PCR using the chromosomal DNA from YEF9246 (lab stock) or YEF8393 (Marquardt et al., 2020) as the template and the pair of primers P1139 and P1140 and then transformed into the YEF9336, YEF9590, or YEF10293 to generate YEF9369, YEF9695, or YEF10315, respectively. |
| ^g^ | pRS316 Myo1-N-GFP (Caviston et al., 2003) was digested with SalI and CalI and then transformed into Masa1243. The transformation mixture was plated on SC-His plate. After 5-FOA and G418 selection, cells were grew on YPD to remove the cover plasmid (pUG23-MYO1). |
| ^h^ | A DNA fragment carrying *arg4Δ::KanMX6* was amplified by PCR from pFA6a-TRP1 using primers Arg4 F1 and Arg4 R1 and then transformed into the YEF473A. |
| ^i^ | A DNA fragment carrying *bni5Δ::NatMX6* was amplified by PCR using the plasmid pAG25 as the template and the pair of primers P1067 and P1087 and then transformed into the YEF473A. |
| ^j^ | EcoRV-digested plasmid YIp128-proACT1-GFP-ECM25-(536-588AA)-tADH1 was integrated into *leu2* locus of YEF8950, YEF9654, YEF2232, or YEF11379 to generate YEF10170, YEF10173, YEF10201, or YEF11489, respectively. |
| ^k^ | BglII-digested plasmid Yip128-CDC3-mCherry was integrated into *CDC3* locus of YEF10276, YEF9654, or YEF11379 to generate YEF10293, YEF10994, or YEF11408, respectively. |
| ^l^ | A DNA fragment carrying *CDC11-GFP-His3MX6* was amplified by PCR using the chromosomal DNA from YEF4940 (lab stock) as the template and the pair of primers P1121 and P1122 and then transformed into YEF473A. |
| ^m^ | XbaI-digested plasmid proHIS3-ymScarlet-I-TUB1-tTUB1-HPH was integrated into *TUB1* locus of YEF473A, YEF11417, YEF11418, YEF9654, YEF11501, YEF9473, YEF8356, or YEF9380 to generate YEF10667, YEF11441, YEF11442, YEF11522, YEF11524, YEF11529, YEF12068, or YEF12069, respectively. |
| ^n^ | A DNA fragment carrying *gin4Δ::URA3-KanMX6* was amplified by PCR using the chromosomal DNA from YEF8122 (lab stock) as the template and the pair of primers P1141 and P1142 and then transformed into YEF9590. |
| ^o^ | A DNA fragment carrying *CDC10-mScarlet-I-URA3MX* was amplified by PCR using the plasmid pFA6a-link-ymScarlet-I-CaURA as the template and the pair of primers P121 and P410 and then transformed into the YEF473A. |
| ^p^ | A DNA fragment carrying *MYO1-mScarlet-I-KanMX* was amplified by PCR using the plasmid pFA6a-link-ymScarlet-I-Kan as the template and the pair of primers P226 and P517 and then transformed into the YEF473A. |
| ^q^ | A DNA fragment carrying *MYO1-mScarlet-I-KanMX* was amplified by PCR using the chromosomal DNA of YEF10958 as the template and the pair of primers Myo1-249-DS-TAA and Myo1-159-UP-TAA and then transformed into the YEF9654 or YEF10296 to generate YEF10993 or YEF11088, respectively. |
| ^r^ | A low-copy plasmid from pUG-BNI5* plasmid series (see genotype column and **Table S2**) was transformed into YEF10993, YEF10994, YEF5804, YEF11277, or YEF11247. |
| ^s^ | BsaBI-digested plasmid bWL715 was integrated into *TUB1* locus of YEF8101 (lab stock). |
| ^t^ | A low-copy plasmid pUG36-BNI5(1-40) was transformed into YEF6349 or YEF11408, to generate YEF11114 or YEF11757, respectively. |
| ^u^ | A DNA fragment carrying *bni5Δ::GBP-URA3MX* was amplified by PCR using the chromosomal DNA from YEF11254 as the template and the pair of primers P1527 and Bni5 381bp DS TAG R and then transformed into the YEF11088 to generate YEF11211. Similarly, A DNA fragment carrying *bni5(1-40)-GBP-URA3MX* was amplified by PCR using the chromosomal DNA from YEF11255 as the template and the pair of primers P1527 and Bni5 381bp DS TAG R and then transformed into the YEF11088 to generate YEF11212. |
| ^v^ | A DNA fragment carrying *bni5Δ::NatMX6* was amplified by PCR using the chromosomal DNA from YEF9654 as the template and the pair of primers P1628 and P1087 and then transformed into the YEF743, YEF11385, YEF11524, YEF11529, YEF10885, YEF7066, YEF11087, YEF11753, YEF11754, or YEF11755 to generate YEF11247, YEF11443, YEF11530, YEF11601, YEF11634, YEF12138, YEF12141, YEF12142, YEF12143, or YEF12144, respectively. |
| ^w^ | A DNA fragment carrying *bni5Δ::GBP-URA3MX*, or *bni5(1-40)-GBP-URA3MX* was amplified by PCR using the plasmid pFA6a-link-GBP-CaURA3 as the template and the pair of primers P1546 and P1167 or P1545 and P1167 and then transformed into the YEF473A. |
| ^x^ | A DNA fragment carrying *elm1Δ::KanMX6* was amplified by PCR using the chromosomal DNA from YEF7515 (lab stock) as the template and the pair of primers P1125 and P1140 and then transformed into the YEF10994. |
| ^y^ | Two DNA fragments carrying distinct halves of *MYO1* gene; 1) chromosomal region from ~250 bp upstream of *MYO1* start codon until residue 990 coding region followed by residues 1181-1193 coding region, and 2) residues 976-990 region followed by residue 1181 coding region to ~130 bp downstream region of *MYO1* were amplified by PCR using the chromosomal DNA from YEF10958 as the template DNA and the pair of primers P1518 and P1578 or P1579 and P1580, respectively. The resultant PCR products were mixed and then transformed into Masa1243. After 5-FOA and G418 selection, cells were grown on YPD to remove the cover plasmid (pUG23-MYO1). |
| ^z^ | A DNA fragment carrying *MYO1-GFP-URA3MX* was amplified by PCR using the chromosomal DNA from YEF9064 (lab stock) as the template DNA and the pair of primers Myo1-159-UP-TAA and Myo1-249-DS-TAA, and then transformed into YEF10667, YEF473A, and YEF11379 to generate YEF11385, YEF11417, and YEF11418, respectively. |
| ^aa^ | A DNA fragment carrying *myo1(1-1797)-KanMX* was amplified by PCR using the chromosomal DNA from YEF10459 (lab stock) as the template DNA and the pair of primers Myo1-4801F and P1580, and then transformed into YEF9473. |
| ^ab^ | AscI-digested plasmid of pRG205MX-proBNI5-yEGFP-BNI5 fusion constructs (see **Table S2**) was integrated into *leu2* locus of YEF11522 to generate YEF11546, YEF11553, or YEF11600, respectively. |
| ^ac^ | XbaI-digested plasmid bWL722 was integrated into *TUB1* locus of YEF11634. |
| ^ad^ | A DNA fragment carrying *CDC10-GFP-His3MX6* was amplified by PCR using the chromosomal DNA from YEF8831 (lab stock) as the template DNA and the pair of primers P812 and P813, and then transformed into YEF5804 and YEF10667 to generate YEF11750 and YEF11753, respectively. |
| ^ae^ | A DNA fragment carrying *CDC11-GFP-His3MX6* was amplified by PCR using the chromosomal DNA from YEF6652 (lab stock) as the template DNA and the pair of primers P814 and P815, and then transformed into YEF10667. |
| ^af^ | A DNA fragment carrying *CDC12-GFP-His3MX6* was amplified by PCR using the chromosomal DNA from YEF6685 (lab stock) as the template DNA and the pair of primers P1137 and P1138, and then transformed into YEF10667. |
| ^ag^ | AscI-digested plasmid pRG205MX-proBNI5-yEGFP-BNI5(FL), pRG205MX-proBNI5-yEGFP-BNI5(Δ41-305), pRG205MX-proBNI5-yEGFP-BNI5(FL)-6A, or pRG205MX-proBNI5-yEGFP-BNI5(FL)-6DE was integrated into *leu2* locus of YEF11640 to generate YEF11772, YEF11773, YEF12456, or YEF12457, respectively. |
| ^ah^ | A low-copy plasmid pUG36-BNI5(306-393) was transformed into YEF5799, YEF5797, or YEF7066 to generate YEF11811, YEF11812, or YEF11819, respectively. |
| ^ai^ | AscI-digested plasmid of pRG205MX-proBNI5-yEGFP-BNI5 fusion constructs (see **Table S2**) was integrated into *leu2* locus of YEF9654 to generate YEF11821, YEF11823, YEF11824, or YEF11825, respectively. |
| ^aj^ | AscI-digested plasmid pRG205MX-proBNI5-yEGFP-BNI5(41-448)-5A, -5DE, -5A', or -5D' was integrated into *leu2* locus of YEF11522 to generate YEF12109, YEF12112, YEF12240, or YEF12241, respectively. |
| ^ak^ | A low-copy plasmid pUG35-HOF1 was transformed into YEF9654. |
| ^al^ | AscI-digested plasmid pRG206MX-proBNI5-yEGFP-BNI5(FL) was integrated into *leu2* locus of YEF12069. |
| ^am^ | A DNA fragment carrying *hof1Δ::TRP1* was amplified by PCR using the chromosomal DNA from YEF5454 (lab stock) as the template DNA and the pair of primers P204 and P486, and then transformed into YEF12194. |
| ^an^ | AscI-digested plasmid pRG205MX-proBNI5-yEGFP-BNI5(41-448), pRG205MX-proBNI5-yEGFP-BNI5(FL), or pRG205MX was integrated into *leu2* locus of YEF12237 to generate YEF12267, YEF12280, or YEF12330, respectively. |
| ^ao^ | Crossed YEF2057 and YEF11501 or YEF9473 to generate YEF12291 or YEF12297, respectively. YEF12291 was grown on 5-FOA plate to generate YEF12293 by curing cover plasmid. |
| ^ap^ | A DNA fragment carrying *GIN4-GFP-His3MX6* or *GIN4-mScarlet-KanMX6* was amplified by PCR using the chromosomal DNA from YEF8298 (lab stock) or YEF10757 (lab stock) as the template DNA and the pair of primers P1120 and P1142, and then transformed into YEF12068 or YEF11821, respectively. |
| ^aq^ | A DNA fragment carrying *ELM1-GFP-His3MX6* was amplified by PCR using the chromosomal DNA from YEF8299 (lab stock) as the template DNA and the pair of primers P1119 and P1140, and then transformed into YEF12068. |
| ^ar^ | AscI-digested plasmid pRG205MX-proBNI5-yEGFP-BNI5(FL)-1A, -1D, -3A, 3D, -6A, or -6DE was integrated into *leu2* locus of YEF11522 to generate YEF12440, YEF12441, YEF12442, YEF12443, YEF12444, or YEF12445, respectively. |
