# Supplementary material for: Bni5 regulates and coordinates septin architecture and myosin-II functions at the cell division site
Source: J Cell Biol. 2025 Nov 6;224(12):e202311040. doi: 10.1083/jcb.202311040 (PMC12591035; doi:10.1083/jcb.202311040)
Supplement: Table S2 — shows plasmids used in this study. [file jcb_202311040_tables2.docx]

| **Plasmid** | **Source** | **Identifier** |
| --- | --- | --- |
| bWL715 (pHIS3p:mRuby2-Tub1+3'UTR::HPH) | (Markus et al., 2015) |  |
| bWL722 (pHIS3p:Venus-Tub1+3'UTR::HPH) | (Markus et al., 2015) |  |
| pAG25 | (Goldstein and McCusker, 1999) |  |
| pCOLA-Duet-[His less]-Shs1 | (Garcia et al., 2011) |  |
| pET His6 Sumo TEV LIC | Scott Gradia | Addgene #29659 |
| pET-His6-Sumo-Bni5 | (Marquardt et al., 2020) | BiLab collection#E2840 |
| pET-His6-Sumo-Elm1_FL_ | This study | BiLab collection#E2952 |
| pET-His6-Sumo-Elm1_1-420_ | This study | BiLab collection#E2951 |
| pET-His6-Sumo-Elm1_420-640_ | This study | BiLab collection#E2973 |
| pET-His6-Sumo-Gin4 | (Marquardt et al., 2024) | BiLab collection#E2568 |
| pFA6a-link-GBP-CaURA3 | This study | BiLab collection#E2506 |
| pFA6a-link-ymScarlet-I-CaURA | (Marquardt et al., 2020) | BiLab collection#E2500 |
| pFA6a-link-yoEGFP-SpHIS5 | (Lee et al., 2013) | Addgene #44836 |
| pFA6a-link-yomApple-GBP-CaURA | (Marquardt et al., 2020) | BiLab collection#E2498 |
| pFA6a-link-yoTagRFP-T-CaURA3 | (Lee et al., 2013) | Addgene #44877 |
| pFA6a-TRP1 | (Longtine et al., 1998b”) |  |
| pFA6a-URA-KanMX6 | (Onishi et al., 2013) |  |
| pGEX-4T1-Elm1^WT^ | (Marquardt et al., 2024) | BiLab collection#E2597 |
| pGEX-4T-1-BNI5(1-448) | This study | BiLab collection#E2946 |
| pGEX-4T-1-BNI5(1-40) | This study | BiLab collection#E2947 |
| pGEX-4T-1-BNI5(306-393) | This study | BiLab collection#E2948 |
| pGEX-4T-1-BNI5(306-393)-6A | This study | BiLab collection#E3160 |
| pGEX-4T-1-BNI5(306-393)-6DE | This study | BiLab collection#E3161 |
| pGEX-4T-1-BNI5(340-448) | This study | BiLab collection#E2949 |
| pGEX-4T-1-BNI5(306-448) | This study | BiLab collection#E2950 |
| pMAL-c2 | New England Biolabs |  |
| pMAL-MYO1-mTD1 | (Fang et al., 2010) | BiLab collection#E2593 |
| pMVB128 | (Versele et al., 2004) |  |
| pMVB133 | (Versele et al., 2004) |  |
| pRG205MX | (Gnugge et al., 2016) | Addgene #64535 |
| pRG206MX | (Gnugge et al., 2016) | Addgene #64536 |
| pRG205MX-proBNI5-yEGFP | This study | BiLab collection#E2773 |
| pRG205MX-proBNI5-yEGFP-BNI5(∆41-305) | This study | BiLab collection#E2793 |
| pRG205MX-proBNI5-yEGFP-BNI5(1-40) | This study | BiLab collection#E2781 |
| pRG205MX-proBNI5-yEGFP-BNI5(306-339) | This study | BiLab collection#E2784 |
| pRG205MX-proBNI5-yEGFP-BNI5(306-448) | This study | BiLab collection#E2792 |
| pRG205MX-proBNI5-yEGFP-BNI5(340-448) | This study | BiLab collection#E2791 |
| pRG205MX-proBNI5-yEGFP-BNI5(41-448) | This study | BiLab collection#E2813 |
| pRG205MX-proBNI5-yEGFP-BNI5(41-448)-5A | This study | BiLab collection#E3022 |
| pRG205MX-proBNI5-yEGFP-BNI5(41-448)-5DE | This study | BiLab collection#E3097 |
| pRG205MX-proBNI5-yEGFP-BNI5(41-448)-5A’ | This study | BiLab collection#E3053 |
| pRG205MX-proBNI5-yEGFP-BNI5(41-448)-5D | This study | BiLab collection#E3054 |
| pRG205MX-proBNI5-yEGFP-BNI5(FL) | This study | BiLab collection#E2780 |
| pRG205MX-proBNI5-yEGFP-BNI5(FL)-1A | This study | BiLab collection#E3044 |
| pRG205MX-proBNI5-yEGFP-BNI5(FL)-1D | This study | BiLab collection#E3055 |
| pRG205MX-proBNI5-yEGFP-BNI5(FL)-3A | This study | BiLab collection#E3112 |
| pRG205MX-proBNI5-yEGFP-BNI5(FL)-3D | This study | BiLab collection#E3113 |
| pRG205MX-proBNI5-yEGFP-BNI5(FL)-6A | This study | BiLab collection#E3116 |
| pRG205MX-proBNI5-yEGFP-BNI5(FL)-6DE | This study | BiLab collection#E3117 |
| pRG206MX-proBNI5-yEGFP-BNI5(FL) | This study | BiLab collection#E3048 |
| proHIS3-ymScarlet-I-TUB1-tTUB1-HPH | (Ghanegolmohammadi et al., 2021) | BiLab collection#E2614 |
| pRS316-N-MYO1-GFP | (Caviston et al., 2003) |  |
| pUG35-HOF1 | Lab Stock | BiLab collection#E1275 |
| pUG36 | J. H. Hegemann |  |
| pUG36-BNI5(∆306-339) | This study^a^ | BiLab collection#E2697 pUG36-BNI5* plasmid series |
| pUG36-BNI5(∆306-393) | This study^b^ | BiLab collection#E2704 pUG36-BNI5* plasmid series |
| pUG36-BNI5(∆340-393) | This study^c^ | BiLab collection#E2677 pUG36-BNI5* plasmid series |
| pUG36-BNI5(∆41-305) | This study^d^ | BiLab collection#E2696 pUG36-BNI5* plasmid series |
| pUG36-BNI5(∆41-339) | This study^d^ | BiLab collection#E2675 pUG36-BNI5* plasmid series |
| pUG36-BNI5(∆41-393) | This study^d^ | BiLab collection#E2680 pUG36-BNI5* plasmid series |
| pUG36-BNI5(1-23) | This study^e^ | BiLab collection#E2070 pUG36-BNI5* plasmid series |
| pUG36-BNI5(1-305) | This study^f^ | BiLab collection#E2069 pUG36-BNI5* plasmid series |
| pUG36-BNI5(1-339) | This study^g^ | BiLab collection#E2671 pUG36-BNI5* plasmid series |
| pUG36-BNI5(1-366fs) | This study^h^ | BiLab collection#E2068 pUG36-BNI5* plasmid series |
| pUG36-BNI5(1-393) | This study^i^ | BiLab collection#E2679 pUG36-BNI5* plasmid series |
| pUG36-BNI5(1-40 340-393) | This study^j^ | BiLab collection#E2678 pUG36-BNI5* plasmid series |
| pUG36-BNI5(1-40) | This study^k^ | BiLab collection#E2666 pUG36-BNI5* plasmid series |
| pUG36-BNI5(306-339 394-448) | This study^l^ | BiLab collection#E2698 pUG36-BNI5* plasmid series |
| pUG36-BNI5(306-339) | This study^m^ | BiLab collection#E2694 pUG36-BNI5* plasmid series |
| pUG36-BNI5(306-393) | This study^n^ | BiLab collection#E2695 pUG36-BNI5* plasmid series |
| pUG36-BNI5(306-448) | This study^o^ | BiLab collection#E2667 pUG36-BNI5* plasmid series |
| pUG36-BNI5(340-393) | This study^p^ | BiLab collection#E2669 pUG36-BNI5* plasmid series |
| pUG36-BNI5(340-448) | This study^q^ | BiLab collection#E2668 pUG36-BNI5* plasmid series |
| pUG36-BNI5(394-448) | This study^r^ | BiLab collection#E2670 pUG36-BNI5* plasmid series |
| pUG36-BNI5(41-305) | This study^s^ | BiLab collection#E2785 pUG36-BNI5* plasmid series |
| pUG36-BNI5(41-339 394-448) | This study^t^ | BiLab collection#E2710 pUG36-BNI5* plasmid series |
| pUG36-BNI5(41-339) | This study^u^ | BiLab collection#E2672 pUG36-BNI5* plasmid series |
| pUG36-BNI5(41-393) | This study^v^ | BiLab collection#E2673 pUG36-BNI5* plasmid series |
| pUG36-BNI5(41-448) | This study^w^ | BiLab collection#E2674 pUG36-BNI5* plasmid series |
| pUG36-BNI5(41-448)-w/oGFP | This study^x^ | BiLab collection#E2942 pUG36-BNI5* plasmid series |
| pUG36-BNI5(FL) | This study^y^ | BiLab collection#E2066 pUG36-BNI5* plasmid series |
| pUG36-BNI5(FL)-w/oGFP | This study^z^ | BiLab collection#E2709 pUG36-BNI5* plasmid series |
| pUG36-BNI5-C-GFP | This study^aa^ | BiLab collection#E2727 pUG36-BNI5* plasmid series |
| pUG36-w/oGFP | This study^ab^ | BiLab collection#E2943 pUG36-BNI5* plasmid series |
| pUG36-GFP-Ecm25-xACT(536–588aa) | (Duan et al., 2021) |  |
| YCp50-MYO1 (CEN *URA3* *MYO1*) | Susan Brown |  |
| YGPM28d13 | Yeast Genomic Tiling Collection |  |
| YIp128-CDC3-mCherry | (Gao et al., 2007) | BiLab collection#E1914 |
| YIp128-proACT1-GFP-ECM25-(536-588AA)-tADH1 | This study | BiLab collection#E2483 |

**Table S2. Plasmids used in this study**

| ^a^ | Constructed by recombination-mediated plasmid construction (Oldenburg et al., 1997) (referred to as gap repair cloning hereafter). Two DNA fragments containing *bni5(1-305)* or *bni5(340-448)* was amplified by PCR from the plasmid pUG36-BNI5(FL) as the template and the pair of primers P1530 and P1573 or P1574 and P680, respectively. Resultant PCR products were mixed and then assembled with EcoRI linearized pUG36 in yeast cells. |
| --- | --- |
| ^b^ | Constructed as ^a^ except primer P1575 was used instead of P1574 to amplify *bni5(394-448)* region instead of *bni5(340-448)* region. |
| ^c^ | Constructed by gap repair cloning. A DNA fragment containing *bni5(394-448)* was amplified by PCR from the plasmid pUG36-BNI5(394-448) as the template and the pair of primers P1542 and P680. Resultant PCR product was then assembled with AflII and HindIII linearized pUG36-BNI5(1-339). |
| ^d^ | Constructed by inverse PCR with gap repair cloning. A DNA fragment containing entire plasmid except *bni5(41-305)*, *bni5(41-339)*, or *bni5(41-393)* region was amplified by PCR from pUG36-BNI5(FL) as the template and the pair of primers P1539 and P1572, P1539 and P1540, or P1539 and P1569, respectively. Resultant PCR products were then self-assembled in yeast cells to generate pUG36-BNI5(∆41-305), pUG36-BNI5(∆41-339), and pUG36-BNI5(∆41-393), respectively. |
| ^e^ | This allele was identified by screening for synthetic lethality with *hof1Δ* (Nishihama et al., 2009). In this allele, there is an *amber* mutation (the codon CAG for Q24 was substituted to TAG, stop codon). A DNA fragment containing this allele was amplified from YEF6928 (lab stock) then assembled into pUG36 by gap repair cloning. |
| ^f^ | This allele was allele identified by screening for synthetic lethality with *hof1Δ* (Nishihama et al., 2009). In this allele, there is an *amber* mutation at the codon for Q306. A DNA fragment containing this allele was amplified from YEF6927 (lab stock) then assembled into pUG36 by gap repair cloning. |
| ^g^ | Constructed by gap repair cloning. A DNA fragment containing *bni5(1-339)* was amplified by PCR from the plasmid pUG36-BNI5(FL) as the template and the pair of primers P1530 and P1538. Resultant PCR product was then assembled with EcoRI linearized pUG36 in yeast cells. |
| ^h^ | This allele was identified by screening for synthetic lethality with *hof1Δ* (Nishihama et al., 2009). In this allele, deletion of G from the codon AGA for R367 caused frameshift, encoding for six extra residues NMIIYP followed by stop codon. A DNA fragment containing this allele was amplified from YEF6926 (lab stock) then assembled into pUG36 by gap repair cloning. |
| ^i^ | Constructed as ^g^ except *bni5(1-393)* region was amplified by the pair of primers P1530 and P1535. |
| ^j^ | Constructed as ^g^ except *bni5(1-40 340-393)* region was amplified from plasmid pUG36-BNI5(∆41-339) by the pair of primers P1530 and P1535. |
| ^k^ | Constructed as ^g^ except *bni5(1-40)* region was amplified by the pair of primers P1530 and P1531. |
| ^l^ | Constructed by gap repair cloning. Two DNA fragments containing *bni5(306-339)* or *bni5(394-448)* was amplified by PCR from the plasmid pUG36-BNI5(306-448) as the template and the pair of primers P1530 and P1541 or P1542 and P680, respectively. Resultant PCR products were mixed and then assembled with EcoRI linearized pUG36 in yeast cells. |
| ^m^ | Constructed as ^g^ except *bni5(306-339)* region was amplified from plasmid pUG36-BNI5(306-448) by the pair of primers P1530 and P1538. |
| ^n^ | Constructed as ^g^ except *bni5(306-393)* region was amplified from plasmid pUG36-BNI5(306-448) by the pair of primers P1530 and P1535. |
| ^o^ | Constructed as ^g^ except *bni5(306-448)* region was amplified from plasmid YGPM28d13 by the pair of primers P1532 and P1533. |
| ^p^ | Constructed as ^g^ except *bni5(340-393)* region was amplified from plasmid YGPM28d13 by the pair of primers P1534 and P1535. |
| ^q^ | Constructed as ^g^ except *bni5(340-448)* region was amplified from plasmid YGPM28d13 by the pair of primers P1534 and P1533. |
| ^r^ | Constructed as ^g^ except *bni5(394-448)* region was amplified from plasmid YGPM28d13 by the pair of primers P1536 and P1533. |
| ^s^ | A ~0.8 kb DNA fragment containing *bni5(288-305-amber)* was acquired by EcoRI and EagI digestion of pUG36-BNI5(1-305) and ligated into EcoRI and EagI digested pUG36-BNI5(41-339). |
| ^t^ | A ~0.4 kb DNA fragment containing *bni5(288-339 394-448)* was acquired by EcoRI and XhoI digestion of pUG36-BNI5(∆340-393) and ligated into EcoRI and XhoI digested pUG36-BNI5(41-339). |
| ^u^ | Constructed as ^g^ except *bni5(41-339)* region was amplified from plasmid YGPM28d13 by the pair of primers P1537 and P1538. |
| ^v^ | Constructed as ^g^ except *bni5(41-393)* region was amplified from plasmid YGPM28d13 by the pair of primers P1537 and P1535. |
| ^w^ | Constructed as ^g^ except *bni5(41-448)* region was amplified from plasmid YGPM28d13 by the pair of primers P1537 and P1533. |
| ^x^ | A ~1.3 kb DNA fragment containing *BNI5(41-448)* was acquired by BamHI and XhoI digestion of pRG205MX-proBNI5-yEGFP-BNI5(41-448) and ligated into ~5.4kb vector backbone of pUG36-BNI5(FL)-w/oGFP acquired by BamHI and XhoI digestion. |
| ^y^ | Constructed as WT control for research of *bni5* alleles identified by screening for synthetic lethality with *hof1Δ* (Nishihama et al., 2009). A DNA fragment containing *BNI5* was assembled into pUG36 by gap repair cloning. |
| ^z^ | To remove the region encoding GFP from pUG36-BNI5(FL), a ~1.4 kb DNA fragment containing *BNI5(FL)* was acquired by SpeI and XhoI digestion of pUG36-BNI5(FL) and ligated into ~5.4kb vector backbone of the same plasmid digested with XbaI and XhoI. SpeI and XbaI generated compatible cohesive ends. |
| ^aa^ | Constructed by gap repair cloning. A DNA fragment containing *BNI5-C-GFP* was amplified by PCR from the chromosomal DNA of YEF9290 as the template and the pair of primers P1166 and P1587. Resultant PCR product was then assembled with ClaI linearized pUG36-BNI5(FL)-w/oGFP in yeast cells. |
| ^ab^ | The GFP region was removed from pUG36 as described in ^z^. SpeI and XbaI digested pUG36 was self-assembled by ligation. |
