# Supplementary material for: Bni5 regulates and coordinates septin architecture and myosin-II functions at the cell division site
Source: J Cell Biol. 2025 Nov 6;224(12):e202311040. doi: 10.1083/jcb.202311040 (PMC12591035; doi:10.1083/jcb.202311040)
Supplement: Table S3 — shows oligonucleotides used in this study. [file jcb_202311040_tables3.docx]

| **Name** | **Sequence** | **Identifier** |
| --- | --- | --- |
| Arg4-F1 | GCTCAAAAGCAGGTAACTATATAACAAGACTAAGGCAAACCGGATCCCCGGGTTAATTAA | P1186 |
| Arg4-R1 | CCAGACCTGATGAAATTCTTGCGCATAACGTCGCCATCTGGAATTCGAGCTCGTTTAAAC | P1187 |
| Bni5 244US ATG | CGGATCTTTGGCAAATGTATG | N/A |
| Bni5 381bp DS TAG R | ACAAAGTTAGCAGGGTTATCGC | N/A |
| Bni5-F tag Check | GTCTGAGCTGGGCAGTATTG | P1168 |
| Bni5-F1 | TGGTGATGCTATGTTAGTGTGAAATAGAACAACAGAAACGCGGATCCCCGGGTTAATTAA | P1067 |
| Bni5-F5 | TGATCGTGGCGAAAATGGCCAATTTTGGATTGGAACTAAAGGTGACGGTGCTGGTTTA | P1166 |
| Bni5-R1(Nat) | TAATTTATAAATATTTATAACAACCCTTGGCGTAATGTAATTCGAGCTCGTTTTCGACAC | P1087 |
| Bni5-R3 | TAATTTATAAATATTTATAACAACCCTTGGCGTAATGTAATCGATGAATTCGAGCTCG | P1167 |
| CDC10+304-3' | GGCACAATCCCTAACCAAAC | P813 |
| CDC10-nt(668-687)-5' | ACAGAAGTGTTAGATCTATC | P812 |
| Cdc11-F-tag check | GTCATCGTCCACCACAACAAG | P1121 |
| Cdc11-R-check | CGATAATGACGATCCACACAAG | P1122 |
| Elm1-C-F | TACTTCCAATCCAATATTATGTCATCGTCCAGTGTG | N/A |
| Elm1-C-R | GCTCGAATTCGGATCCCTATATTTGACCATTATCTGCAAAG | N/A |
| Elm1-F1 | TTTTTTGAACGCCAGGTTAACAATAATTACTTAGCATGAACGGATCCCCGGGTTAATTAA | P1125 |
| Elm1-FL-F | TACTTCCAATCCAATATTATGTCACCTCGACAGCTTATACCG | N/A |
| Elm1-FL-R | GCTCGAATTCGGATCCCTATATTTGACCATTATCTGCAAAG | N/A |
| Elm1-F-tag check | CCTAAAGAGAACGGGAACAGAAC | P1119 |
| Elm1-N-F | TACTTCCAATCCAATATTATGTCACCTCGACAGCTT | N/A |
| Elm1-N-R | GCTCGAATTCGGATCCTTAAATTTGACTGTGATTCCTAGAA | N/A |
| Elm1-R-check | GATTTCGCGACACAGTGG | P1140 |
| F045-INN1-Seq-2 | GATAAAAACATAGAAAGG | P45 |
| F206-HOF1-350up-from-Start | CCCTTTCGGTAGTTTGCGATT | P204 |
| F228-MYO1-tag-F5 | AAAAATATTGATAGTAACAATGCACAGAGTAAAATTTTCAGTGGTGACGGTGCTGGTTTA | P226 |
| F260-yeGFP-XbaI | TCTAGAATGTCTAAAGGTGAAG | P254 |
| F261-ECM25(aa536-588)-tADH1 | GAACGTTTACAAGGTTAAGGCGCGCCACTTCTAAATAAGCGAATTTCTTATG | P255 |
| Felm1-check | GAGGAACTTACTTGATCCTTCTTGAAG | P1139 |
| Fgin4-check | CACTTCACTGGAAGAACTGGG | P1141 |
| GBP-F | GACGGTGCTGGTTTAATGGCACAAGTTCAATTGGTTGAA | N/A |
| GBP-R | TTTAGAAGTGGCGCGTTAATGGTGGTGATGGTGATGAGA | N/A |
| Gin4-F-tag check | GGAATTGTATGCCAAGATTTCTG | P1120 |
| M13(-20)Forward | GTAAAACGACGGCCAGTGAA | P680 |
| Myo1-159-UP-TAA | CTAGCGAATAAAAATAGAAGCGA | N/A |
| Myo1-249-DS-TAA | GATACGGGGTGAAAGAGTT | N/A |
| Myo1-4801F | AAAGCTGAAACAAACTTAAA | N/A |
| Myo1-F1 | GAAGATCATAACAAAGTTAGACAGGACAACAACAGCAATACGGATCCCCGGGTTAATTAA | P1227 |
| Myo1-R1 | AAAGGATATAAAGTCTTCCAAATTTTTAAAAAAAAGTTCGGAATTCGAGCTCGTTTAAAC | P1228 |
| P1518-F-Myo1-250up-Start | AACGCTGAGATAGCTTTTCTTAC | P1518 |
| P1526-F-Bni5-(220) | AGAACAAGTTAGACGCTGAACTTG | P1526 |
| P1527-F-Bni5-250up-ATG | TGGCAAATGTATGTAGCACTTCTC | P1527 |
| P1530-F-GFP(aa150)-in-pUG36 | TCACAATGTTTACATCATGGCTGAC | P1530 |
| P1531-R-pUG36-link-Bni5(40)-Stop | ATGACTCGAGGTCGACGGTATCGATAAGCTTGATATCTTAAGCTTCGTCCTCCGCAGGTT | P1531 |
| P1532-F-pUG36-link-ATG-Bni5(306) | GTACAAATCTAGAACTAGTGGATCCCCCGGGCTGCAGATGCAGAAAAAAATGGCGAATTT | P1532 |
| P1533-R-pUG36-link-Bni5(448)-Stop | ATGACTCGAGGTCGACGGTATCGATAAGCTTGATATCTTATTTAGTTCCAATCCAAAATT | P1533 |
| P1534-F-pUG36-link-ATG-Bni5(340) | GTACAAATCTAGAACTAGTGGATCCCCCGGGCTGCAGATGTCACTAAATAAGTATGATTC | P1534 |
| P1535-R-pUG36-link-Bni5(393)-Stop | ATGACTCGAGGTCGACGGTATCGATAAGCTTGATATCTTAAGAACCCTGTGCATTCATCT | P1535 |
| P1536-F-pUG36-link-ATG-Bni5(394) | GTACAAATCTAGAACTAGTGGATCCCCCGGGCTGCAGATGTTATCTATGGAAGACGGAAA | P1536 |
| P1537-F-pUG36-link-ATG-Bni5(41) | GTACAAATCTAGAACTAGTGGATCCCCCGGGCTGCAGATGGTCGAAGATAATGTCAAGGA | P1537 |
| P1538-R-pUG36-link-Bni5(339)-Stop | ATGACTCGAGGTCGACGGTATCGATAAGCTTGATATCTTAAGATTTACGACTTCCGTTTC | P1538 |
| P1539-R-Bni5(40) | AGCTTCGTCCTCCGCAGGTTCAACTAATTGTAAATTTTCG | P1539 |
| P1540-F-Bni5(40)-Bni5(340) | AACCTGCGGAGGACGAAGCTTCACTAAATAAGTATGATTCGCCAGTCTCCTCTCCTATCA | P1540 |
| P1541-R-Bni5(339) | AGATTTACGACTTCCGTTTCTTGAATTGGAATTGCTGCTC | P1541 |
| P1542-F-Bni5(339)-Bni5(394) | GAAACGGAAGTCGTAAATCTTTATCTATGGAAGACGGAAAGAGACTACATAGAGCCGTAG | P1542 |
| P1545-F-Bni5(40)-F5 | CGAAAATTTACAATTAGTTGAACCTGCGGAGGACGAAGCTGGTGACGGTGCTGGTTTA | P1545 |
| P1546-F-Bni5(1)-F5 | TGATGCTATGTTAGTGTGAAATAGAACAACAGAAACGATGGGTGACGGTGCTGGTTTA | P1546 |
| P1569-F-Bni5(40)-Bni5(394) | AACCTGCGGAGGACGAAGCTTTATCTATGGAAGACGGAAAGAGACTACATAGAGCCGTAG | P1569 |
| P1572-F-Bni5(40)-Bni5(306) | AACCTGCGGAGGACGAAGCTCAGAAAAAAATGGCGAATTTCGAGACACGACGCCCTACAA | P1572 |
| P1573-R-Bni5(305) | TCCATCACCATCTGAATTAAAGTGATTTAT | P1573 |
| P1574-F-Bni5(305)-Bni5(340) | TTAATTCAGATGGTGATGGATCACTAAATAAGTATGATTCGCCAGTCTCCTCTCCTATCA | P1574 |
| P1575-F-Bni5(305)-Bni5(394) | TTAATTCAGATGGTGATGGATTATCTATGGAAGACGGAAAGAGACTACATAGAGCCGTAG | P1575 |
| P1578-R-MYO1-delta-mTD1 | TTCCAATTCTTTAATTGATATTTGCTTTATTAGTTCGTTATTTTTTGAGTGTAATTTCTC | P1578 |
| P1579-F-MYO1-delta-mTD1 | AACTAATAAAGCAAATATCAATTAAAGAATTGGAAGCTCGGTTGTCACAGGAAATATCC | P1579 |
| P1580-R-MYO1-130down-stop | ACTTAGTATATAACGCTCGTGTCGTC | P1580 |
| P1581-F-BNI5pro-SacI-pRG205MX | TATATTTCTTTTCGCGAGCTCGCATTGGGAGTCTATCATAACGTATTTATATATCCTCAT | P1581 |
| P1582-R-EGFP-link-pRG205MX | CAGCCCGGGGGATCCACTAGTTCTAGACTTGTATAATTCATCCATGCCCAACGTTATACC | P1582 |
| P1628-F-Bni5-802up-from-ATG | TCGCATTGGGAGTCTATCATAACG | P1628 |
| P1753-BNI5-S270D-T274E-F | GATGGAGGAAGCGAACCCTTAGATTCTCAAAC | P1753 |
| P1754-BNI5-S270D-T274E-R | TTCGCTTCCTCCATCGCGATTGGGACAATTCTC | P1754 |
| P1755-BNI5-S270A-S274A-F | GCTGGAGGAAGCGCTCCCTTAGATTCTCAAAC | P1755 |
| P1756-BNI5-S270A-S274A-R | AGCGCTTCCTCCAGCGCGATTGGGACAATTCTC | P1756 |
| P1793-BNI5-S346D-S349D-S350D-F | GATCCAGTCGATGATCCTATCACATCAGCGTCTGAGCTG | P1793 |
| P1794-BNI5-S346D-S349D-S350D-R | AGGATCATCGACTGGATCATCATACTTATTTAGTGAAGATTTAC | P1794 |
| P1795-BNI5-S346A-S349A-S350A-F | GCTCCAGTCGCTGCTCCTATCACATCAGCGTCTGAGCTG | P1795 |
| P1796-BNI5-S346A-S349A-S350A-R | AGGAGCAGCGACTGGAGCATCATACTTATTTAGTGAAGATTTAC | P1796 |
| P1869-BNI5-S13A-F | GCTTGCGCAAATAGAGATAGATA | P1869 |
| P1870-BNI5-S13A-R | TTGCGCAAGCCTCTTCTTTATC | P1870 |
| P1904-BNI5-S13D-F | GCTTGATCAAATAGAGATAGATATC | P1904 |
| P1905-BNI5-S13D-R | TTGATCAAGCCTCTTCTTTATC | P1905 |
| P1974-S129A-F | TATAATGCGTTTGTCGCAAATTCCGCTGG | P1974 |
| P1975-S270A-R | TAAGGGAGTGCTTCCTCCCGCGCGATTGGGACAATTCTCT | P1975 |
| P1976-S129A-R | GACAAACGCATTATAGGAGTCACCAATAGC | P1976 |
| P1977-S278A-F | GGAAGCACTCCCTTAGATGCGCAAACTAAAATTTTTATCCC | P1977 |
| P1978-S129D-F | TATAATGATTTTGTCGCAAATTCCGCTGG | P1978 |
| P1979-S270D-R | TAAGGGAGTGCTTCCTCCATCGCGATTGGGACAATTCTCT | P1979 |
| P1980-S129D-R | GACAAAATCATTATAGGAGTCACCAATAGC | P1980 |
| P1981-S278D-F | GGAAGCACTCCCTTAGATGATCAAACTAAAATTTTTATCCC | P1981 |
| P1982-S325A-S327A-S328A-F | ATCGCGGTGGCGGCGAATTCCAATTCAAGAAACGG | P1982 |
| P1983-S349A-T353A-S356A-R | CGCCGCTGACGCGATAGGAGACGCGACTGGCGAATCATAC | P1983 |
| P1984-S325A-S327A-S328A-R | CGCCGCCACCGCGATAACTCTGAACGGATTTGTAG | P1984 |
| P1984-S325A-S327A-S328A-R | CGCCGCCACCGCGATAACTCTGAACGGATTTGTAG | P1984 |
| P1985-S349A-T353A-S356A-F | ATCGCGTCAGCGGCGGAGCTGGGCAGTATTGCCAAG | P1985 |
| P1985-S349A-T353A-S356A-F | ATCGCGTCAGCGGCGGAGCTGGGCAGTATTGCCAAG | P1985 |
| P1986-S325D-S327D-S328D-F | ATCGATGTGGATGATAATTCCAATTCAAGAAACGG | P1986 |
| P1987-S349D-T353E-S356D-R | ATCCGCTGACTCGATAGGAGAATCGACTGGCGAATCATAC | P1987 |
| P1988-S325D-S327D-S328D-R | ATCATCCACATCGATAACTCTGAACGGATTTGTAG | P1988 |
| P1989-S349D-T353E-S356D-F | ATCGAGTCAGCGGATGAGCTGGGCAGTATTGCCAAG | P1989 |
| R108-GBP-with-AscI-term | GTCATGGCGCGCCTTAATGGTGGTGATGGTG | P425 |
| R169-HOF1-350down-from-Stop | TTCGTAACAAGTGACTCTAATGATA | P486 |
| R200-MYO1-tag-R3 | TAATGCATATTCTCATTCTGTATATACAAAACATCTCATCATTCGATGAATTCGAGCTCG | P517 |
| R238-ECM25(aa536-588)-TAA | TTAACCTTGTAAACGTTCTTCGTAC | P549 |
| R239-GFP-proACT1 | CCTTTAGACATTCTAGATGTTAATTCAGTAAATTTTCGATCTTGGGAAG | P550 |
| Rgin4-check | GCTCTTACTTTAATCCCAAAGAGG | P1142 |
| Shs1-AMP-520 | ACCACCTTTTTCCATACGA | Y178 |
| Shs1-R-check | GCTTTACTTTCTGACCTTCG | P1118 |

**Table S3. Oligonucleotides used in this study**
