# Supplementary material for: Bni5 regulates and coordinates septin architecture and myosin-II functions at the cell division site
Source: J Cell Biol. 2025 Nov 6;224(12):e202311040. doi: 10.1083/jcb.202311040 (PMC12591035; doi:10.1083/jcb.202311040)
Supplement: SourceData F3 — is the source file for Fig. 3. [file jcb_202311040_sourcedataf3.pdf]

# Figure 3C

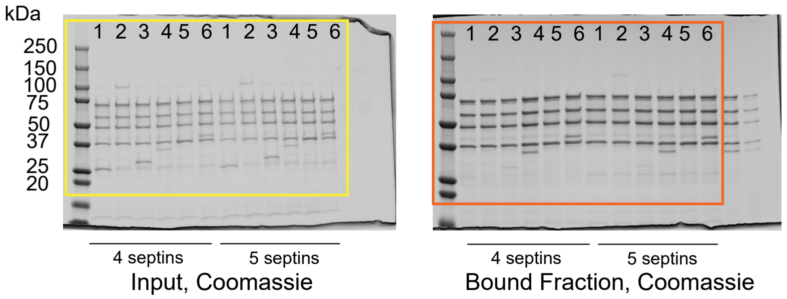


1: GST
2: GST-Bni5-FL (aa1-448)
3: GST-Bni5-HR1 (aa1-40)
4: GST-Bni5-Ext-HR2 (aa306-393)
5: GST-Bni5-HR2-HR3 (aa340-448)
6: GST-Bni5-Ext-HR2-HR3 (aa306-448)

Yellow box indicates cropped region used in Figure 3C (left)
Orange box indicates cropped region used in Figure 3C (right)

# Figure 3F

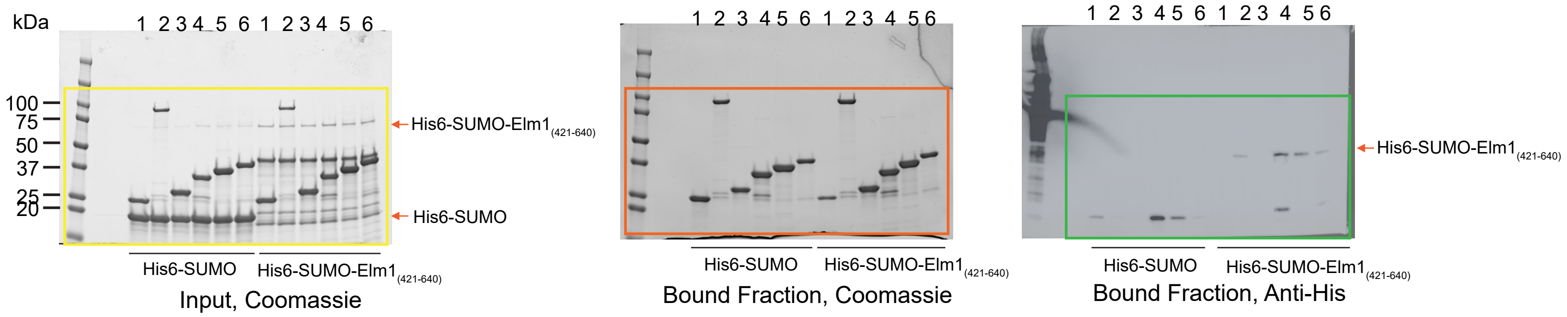


Yellow box indicates cropped region used in Figure 3F (top)
Orange box indicates cropped region used in Figure 3F (bottom left)
Green box indicates cropped region used in Figure 3F (bottom right)

1: GST
2: GST-Bni5-FL (aa1-448)
3: GST-Bni5-HR1 (aa1-40)
4: GST-Bni5-Ext-HR2 (aa306-393)
5: GST-Bni5-HR2-HR3 (aa340-448)
6: GST-Bni5-Ext-HR2-HR3 (aa306-448)
