# Supplementary material for: Bni5 regulates and coordinates septin architecture and myosin-II functions at the cell division site
Source: J Cell Biol. 2025 Nov 6;224(12):e202311040. doi: 10.1083/jcb.202311040 (PMC12591035; doi:10.1083/jcb.202311040)
Supplement: SourceData F5 — is the source file for Fig. 5. [file jcb_202311040_sourcedataf5.pdf]

Figure 5D

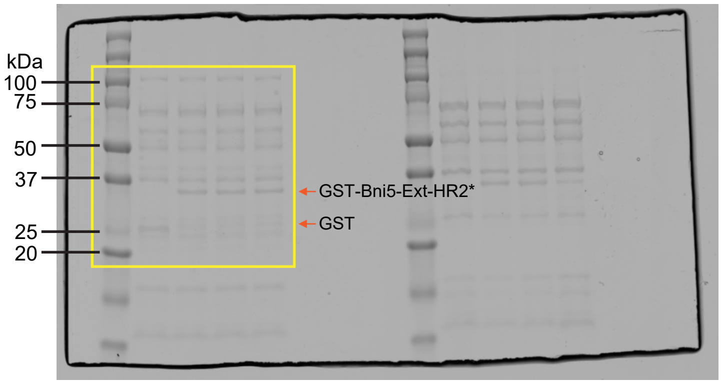

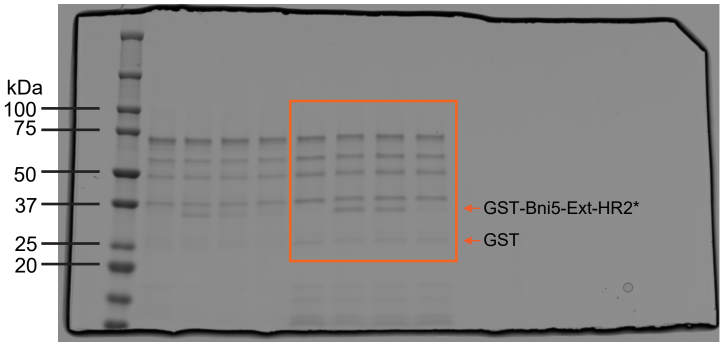

Yellow box indicates cropped region used in Figure 5D (left)
Orange box indicates cropped region used in Figure 5D (right)

1: GST
2: GST-Bni5-Ext-HR2 (aa306-393)
3: GST-Bni5-Ext-HR2-6A (aa306-393)
4: GST-Bni5-Ext-HR2-6DE (aa306-393)
