# Supplementary material for: Bni5 regulates and coordinates septin architecture and myosin-II functions at the cell division site
Source: J Cell Biol. 2025 Nov 6;224(12):e202311040. doi: 10.1083/jcb.202311040 (PMC12591035; doi:10.1083/jcb.202311040)
Supplement: SourceData F6 — is the source file for Fig. 6. [file jcb_202311040_sourcedataf6.pdf]

Figure 6F

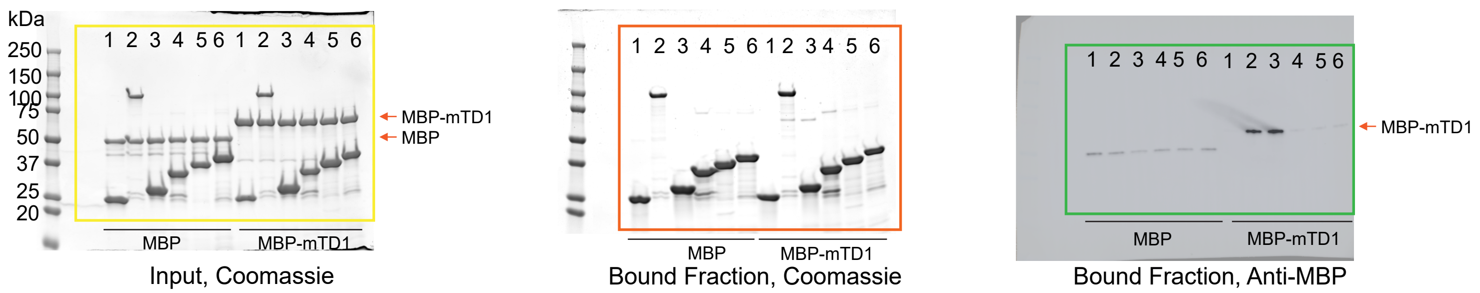

1: GST
2: GST-Bni5-FL (aa1-448)
3: GST-Bni5-HR1 (aa1-40)
4: GST-Bni5-Ext-HR2 (aa306-393)
5: GST-Bni5-HR2-HR3 (aa340-448)
6: GST-Bni5-Ext-HR2-HR3 (aa306-448)

Yellow box indicates cropped region used in Figure 6F (top)
Orange box indicates cropped region used in Figure 6F (bottom left)
Green box indicates cropped region used in Figure 6F (bottom right)
