# Supplementary material for: Bni5 regulates and coordinates septin architecture and myosin-II functions at the cell division site
Source: J Cell Biol. 2025 Nov 6;224(12):e202311040. doi: 10.1083/jcb.202311040 (PMC12591035; doi:10.1083/jcb.202311040)
Supplement: SourceData FS2 — is the source file for Fig. S2. [file jcb_202311040_sourcedatafs2.pdf]

Figure S2B

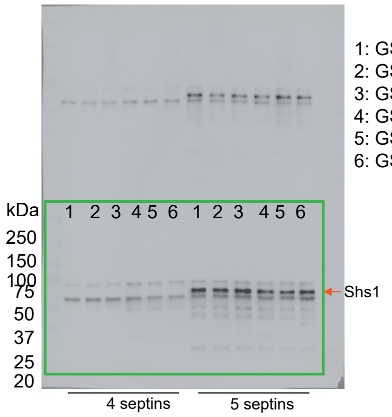

Bound Fraction, Anti-shs1

1: GST
2: GST-Bni5-FL (aa1-448)
3: GST-Bni5-HR1 (aa1-40)
4: GST-Bni5-Ext-HR2 (aa306-393)
5: GST-Bni5-HR2-HR3 (aa340-448)
6: GST-Bni5-Ext-HR2-HR3 (aa306-448)

Green box indicates cropped region used in Figure S2B

Figure S2C

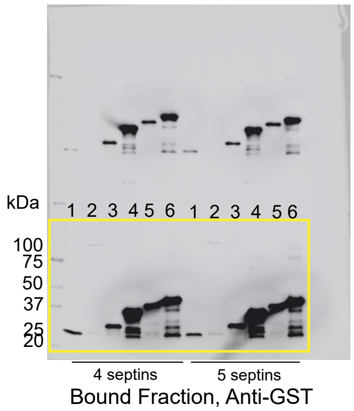

Bound Fraction, Anti-GST
Original samples

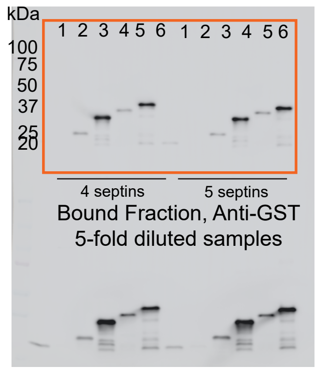

Bound Fraction, Anti-GST
5-fold diluted samples

1: GST
2: GST-Bni5-FL (aa1-448)
3: GST-Bni5-HR1 (aa1-40)
4: GST-Bni5-Ext-HR2 (aa306-393)
5: GST-Bni5-HR2-HR3 (aa340-448)
6: GST-Bni5-Ext-HR2-HR3 (aa306-448)

Yellow box indicates cropped region used in Figure S2C (left)
Orange box indicates cropped region used in Figure S2C (right)
